# VGLL4 enhances PDK4 transcription and promotes metabolic dysfunction-associated fatty liver disease

**DOI:** 10.1038/s44319-026-00934-x

**Published:** 2026-09-22

**Authors:** Yixing Chen, Xiaodong Wang, Wentao Yu, Meixi Gong, Yi Lu, Jinjin Xu, Hao Zhang, Ruizeng Yang, Haoen Zhang, Haoran Gao, Zhuoqun Gao, Jiayan Yan, Zhifeng Jiang, Shiyu Zhang, Xueyan Ma, Jing Yu, Fang Wei, Qiang Fu, Yi Zhou, Jian Zhou, Jian Wan, Yunyun Jin, Lei Zhang

**Affiliations:** 1 Key Laboratory of Multi-Cell Systems, Shanghai Institute of Biochemistry and Cell Biology, Center for Excellence in Molecular Cell Science, Chinese Academy of Sciences, University of Chinese Academy of Sciences, Shanghai, China; 2 Department of Urology, Shanghai Sixth People’s Hospital Affiliated to Shanghai Jiao Tong University School of Medicine, Shanghai Eastern Institute of Urologic Reconstruction, Shanghai Jiao Tong University, Shanghai, China; 3 Key Laboratory of Systems Health Science of Zhejiang Province, School of Life Science, Hangzhou Institute for Advanced Study, University of Chinese Academy of Sciences, Hangzhou, China; 4 Sheng Yushou Center of Cell Biology and Immunology, School of Life Sciences and Biotechnology, Shanghai Jiao Tong University, Shanghai, China; 5 The Academy for Cell and Life Health, Medical School and Faculty of Life Science and Technology, Kunming University of Science and Technology, Kunming, China; 6 Department of Hepatobiliary Surgery and Liver Transplantation, Liver Cancer Institute, Zhongshan Hospital, Fudan University; Key Laboratory of Carcinogenesis and Cancer Invasion (Fudan University), Ministry of Education, Shanghai, China; 7 School of Basic Medical Sciences, Dali University, Yunnan, China; 8 Institute of Neuroscience, State Key Laboratory of Neuroscience, CAS Center for Excellence in Brain Science and Intelligence Technology, Chinese Academy of Sciences, University of Chinese Academy of Sciences, Shanghai, China; 9 Department of Emergency and Critical Care Medicine, Shanghai Pudong New Area People’s Hospital, Shanghai, China

**Keywords:** Chromatin, Transcription & Genomics, Molecular Biology of Disease, Signal Transduction

## Abstract

MAFLD is a global epidemic with a limited number of approved targeted therapies, underscoring the urgent need to define its underlying regulatory mechanisms. Here, we identify VGLL4 as a positive regulator of MAFLD progression. Hepatocyte-specific overexpression of *Vgll4* induces hepatic steatosis, insulin resistance, and inflammation, whereas its knockout protects mice from HFD-induced metabolic dysfunction. Mechanistically, VGLL4 strengthens the interaction between TEAD4 and CEBPA and promotes the cooperative activation of *Pdk4* expression. Functional studies identify PDK4 as a downstream effector, as its knockdown alleviates VGLL4-exacerbated hepatosteatosis in vivo. YAP attenuates this regulatory mechanism and suppresses *Pdk4* transcription during hepatic steatosis. Notably, administration of Super-TDU, a peptide that disrupts the VGLL4–TEADs interaction, significantly alleviates hepatic steatosis in both HFD-fed mice and *Vgll4*-overexpressing mice. Our findings unveil the VGLL4–TEAD4/CEBPA–PDK4 axis as a key driver of MAFLD and demonstrate that its pharmacological blockade alleviates hepatic steatosis in model mice.

## Synopsis

Hepatic VGLL4 promotes MAFLD by enhancing steatosis and impairing insulin signaling. Mechanistically, VGLL4 promotes TEAD4/CEBPA-dependent PDK4 transcription, while disrupting VGLL4/TEAD4 binding alleviates fatty liver phenotypes.

- VGLL4 acts as a hepatocyte-specific driver of MAFLD progression.
- VGLL4 enhances TEAD4-CEBPA cooperation to activate *Pdk4* transcription and promote hepatic lipid accumulation.
- Targeting the VGLL4/TEADs complex with Super-TDU represents a viable strategy to halt MAFLD progression.

## Abstract (web summary)

Hepatic VGLL4 promotes MAFLD by enhancing steatosis and impairing insulin signaling. Mechanistically, VGLL4 promotes TEAD4/CEBPA-dependent PDK4 transcription, while disrupting VGLL4/TEAD4 binding alleviates fatty liver phenotypes.

## Graphical abstract

**Figure:**
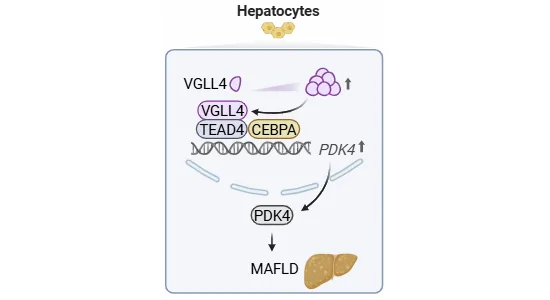

## Introduction

Defined as the presence of at least 5% hepatic fat accumulation in the liver, metabolic dysfunction-associated fatty liver disease (MAFLD) has emerged as a global epidemic, paralleling the growing prevalence of diabetes and metabolic syndrome (Friedman et al, 2018; Eslam et al, 2020). Lipotoxicity triggered by excessive lipid accumulation can cause endoplasmic reticulum stress and mitochondrial dysfunction, leading to MASH, which is characterized by inflammation and hepatocyte ballooning degeneration (Ferguson and Finck, 2021). Notably, previous studies have delineated transcriptional cascades that orchestrate the complex temporal dynamics of hepatosteatosis pathogenesis and progression (Govaere et al, 2020; Fang et al, 2020). However, regulatory mechanisms underlying MAFLD progression remain unclear, impeding the development of disease-specific therapies.

Converging evidence places the Hippo signaling pathway at the nexus of hepatic growth control and metabolic homeostasis in MAFLD. Core kinases such as large tumor suppressor kinase 2 (LATS2) and mammalian sterile 20-like kinase 1 (MST1) safeguard hepatic lipid equilibrium by constraining de novo lipogenesis and free-cholesterol accumulation through the sterol regulatory element-binding protein (SREBP) and adenosine 5’-monophosphate-activated protein kinase (AMPK)/SREBP2 axis (Aylon et al, 2016; Wang et al, 2025). Turning to the downstream effectors of Hippo signaling, Yes-associated protein (YAP) and transcriptional coactivator with PDZ-binding motif (TAZ) have been implicated in the transition from steatosis to steatohepatitis, promoting fibrogenesis in a cell type-dependent manner (Wang et al, 2016; Song et al, 2020; Driskill and Pan, 2021). Collectively, these findings underscore an important role of the Hippo cascade in liver metabolism, where balanced signaling maintains lipid homeostasis while its dysregulation contributes to disease progression. Within this regulatory framework, the transcriptional cofactor vestigial-like protein 4 (VGLL4) emerges as a critical yet understudied node. VGLL4 is a member of the vestigial-like (VGLL) family characterized by two Tondu (TDU) domains (Chen et al, 2004). Its Drosophila ortholog, Tgi/SdBP, antagonizes the Scalloped–Yorkie complex, whereas mammalian VGLL4 similarly competes with YAP for binding to TEA domain transcription factors (TEADs), thereby modulating TEAD-dependent transcription (Guo et al, 2013; Koontz et al, 2013; Zhang et al, 2014b). Beyond its canonical tumor-suppressive role, VGLL4 exhibits a context-dependent activity through TEADs by engaging tissue-specific transcriptional partners, such as runt-related transcription factor 2 (RUNX2) in osteoblast differentiation, myogenic differentiation 1 (MyoD) in myogenesis, and C-terminal binding protein 2 (CTBP2) during early adipogenesis (Zhang et al, 2018b; Feng et al, 2019; Suo et al, 2020). However, the hepatic function of VGLL4, particularly its involvement in lipid and glucose metabolic remodeling during MAFLD, remains unexplored. This study therefore investigates whether VGLL4, acting through TEADs and hepatocyte-enriched transcription factors, reprograms metabolic transcription to drive hepatic steatosis and disease progression.

Pyruvate dehydrogenase kinase isozyme 4 (PDK4) is a key regulator of hepatic glucose metabolism and has been implicated in hepatic steatosis (Zhang et al, 2014a, 2018a). By phosphorylating and inactivating the pyruvate dehydrogenase complex (PDC), PDK4 limits the oxidative conversion of pyruvate to acetyl-CoA, thereby suppressing glucose oxidation and favoring gluconeogenic and fatty acid-dependent metabolic programs (Zhang et al, 2014a; Jeoung, 2015). Elevated hepatic PDK4 expression is associated with increased glucose production, insulin resistance, and lipid accumulation. Consistent with this, genetic deletion of PDK4 or pharmacological inhibition of hepatic PDKs attenuates hepatic steatosis, accompanied by AMPK activation, reduced nuclear accumulation of carbohydrate-responsive element-binding protein (ChREBP), and downregulation of lipogenic genes, including ACC1, FASN, and SCD1 (Wu et al, 2018; Zhang et al, 2018a). However, the upstream mechanisms governing PDK4 dysregulation during MAFLD progression remain poorly understood.

Here, we provide compelling evidence that VGLL4 exerts a pro-steatotic effect during hepatic steatosis progression via its interaction with TEADs. We first observed that VGLL4 is enriched in MAFLD patients’ hepatocytes and upregulated as MAFLD progresses. Harnessing mouse models with either *Vgll4* overexpression or conditional *Vgll4* knockout, we further demonstrate that VGLL4 acts as a positive regulator in the progression of hepatosteatosis, insulin resistance, and inflammation in vivo. VGLL4’s pro-steatotic function is also conserved in a human hepatic steatosis model in vitro. Mechanistically, we further identify that VGLL4 induces hepatic lipid accumulation by enhancing the interaction between TEAD4 and CCAAT/enhancer-binding protein alpha (CEBPA). Moreover, we show that VGLL4 enhances TEAD4/CEBPA-dependent transcription of *Pdk4*, which functions as an important downstream effector of VGLL4 in MAFLD regulation. YAP antagonizes this regulatory mechanism during hepatic steatosis. Importantly, our findings indicate that Super-TDU administration mitigates hepatosteatosis induced by *Vgll4* overexpression and high-fat diet (HFD) feeding. These results provide key mechanistic insights and suggest that pharmacological inhibition of this pathway using Super-TDU could represent an effective therapeutic strategy for MAFLD.

## Results

### Hepatocyte-specific *Vgll4* overexpression induces hepatic steatosis

**Figure EV1:**
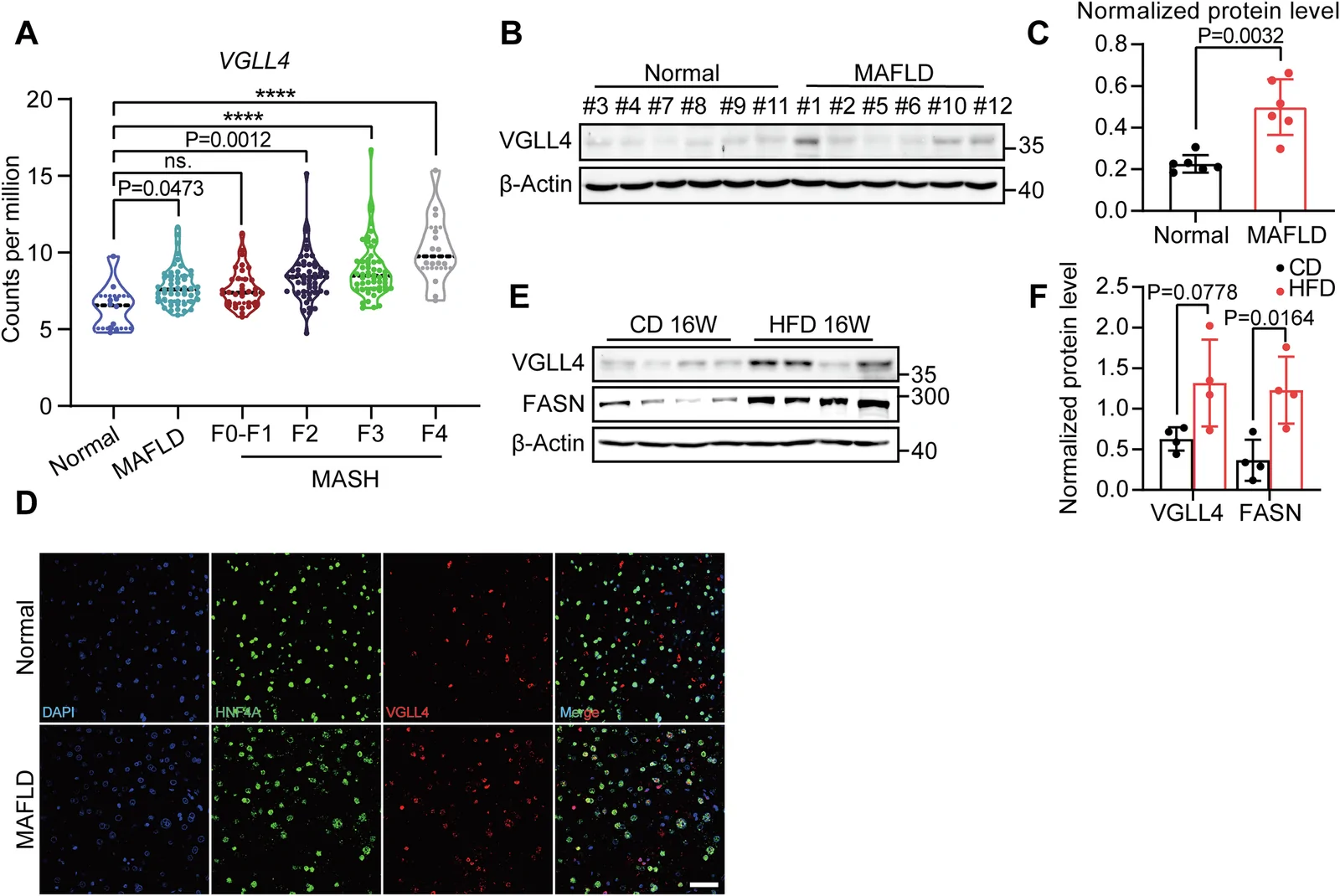
VGLL4 protein expression is elevated in a subset of MAFLD liver samples. (**A**) Violin plot depicting *VGLL4* expression in normal liver tissues, MAFLD liver tissues, and MASH liver tissues stratified by fibrosis stage based on the GSE135251 dataset (*n* = 10–54). (**B**) Western blot analysis of VGLL4 protein levels in liver tissues from controls and patients with MAFLD (*n* = 6). Samples were derived from the same experiment and blots were processed in parallel. (**C**) Quantification of VGLL4 protein levels normalized to β-actin (*n* = 6). (**D**) Immunofluorescence staining for VGLL4 and HNF4A in liver tissues from the indicated groups (scale bar, 20 µm). (**E**) Western blot analysis of protein levels of VGLL4 and FASN in liver tissues from CD and HFD mice (*n* = 4). Samples were derived from the same experiment and blots were processed in parallel. (**F**) Quantification of VGLL4 and FASN protein levels normalized to β-actin (*n* = 4). Data information: Data are presented as mean ± SD. Biological replicates are shown in (**A**). For (**C**, **F**), significance was determined by Welch’s *t* test. For (**A**), significance was determined using one-way ANOVA. ****P < 0.0001, ns. indicates no significant difference. MAFLD metabolic dysfunction-associated fatty liver disease, CD chow diet, HFD high-fat diet. Source data are available online for this figure

Analysis of the public transcriptomic dataset GSE135251 showed that *VGLL4* mRNA was elevated in livers from MAFLD patients versus controls (Fig. EV1A) (Govaere et al, 2020). This transcriptional upregulation was subsequently corroborated at the protein level in patient-derived liver samples, with Western blot analysis demonstrating increased VGLL4 in a subset of MAFLD cases (Fig. EV1B,C). To determine whether VGLL4 upregulation occurred in hepatocytes, we performed co-immunofluorescence staining for VGLL4 and the hepatocyte marker HNF4α. Compared with control liver tissues, MAFLD liver sections showed increased VGLL4 staining that was predominantly detected in HNF4α-positive hepatocytes (Fig. EV1D). To enhance the pathological relevance of the result, we collected liver samples from mice fed a standard chow diet (CD) or an HFD for 16 weeks and performed western blot analysis to assess VGLL4 protein levels. Compared with CD-fed controls, HFD-fed mice showed an apparent increase in hepatic VGLL4 protein in three of the four samples; however, the difference did not reach statistical significance (Fig. EV1E,F). These data suggest a tendency toward VGLL4 induction during HFD-induced hepatic steatosis, while also indicating inter-individual variability, consistent with the heterogeneous VGLL4 upregulation observed in human MAFLD liver samples (Fig. EV1B).

**Figure 1:**
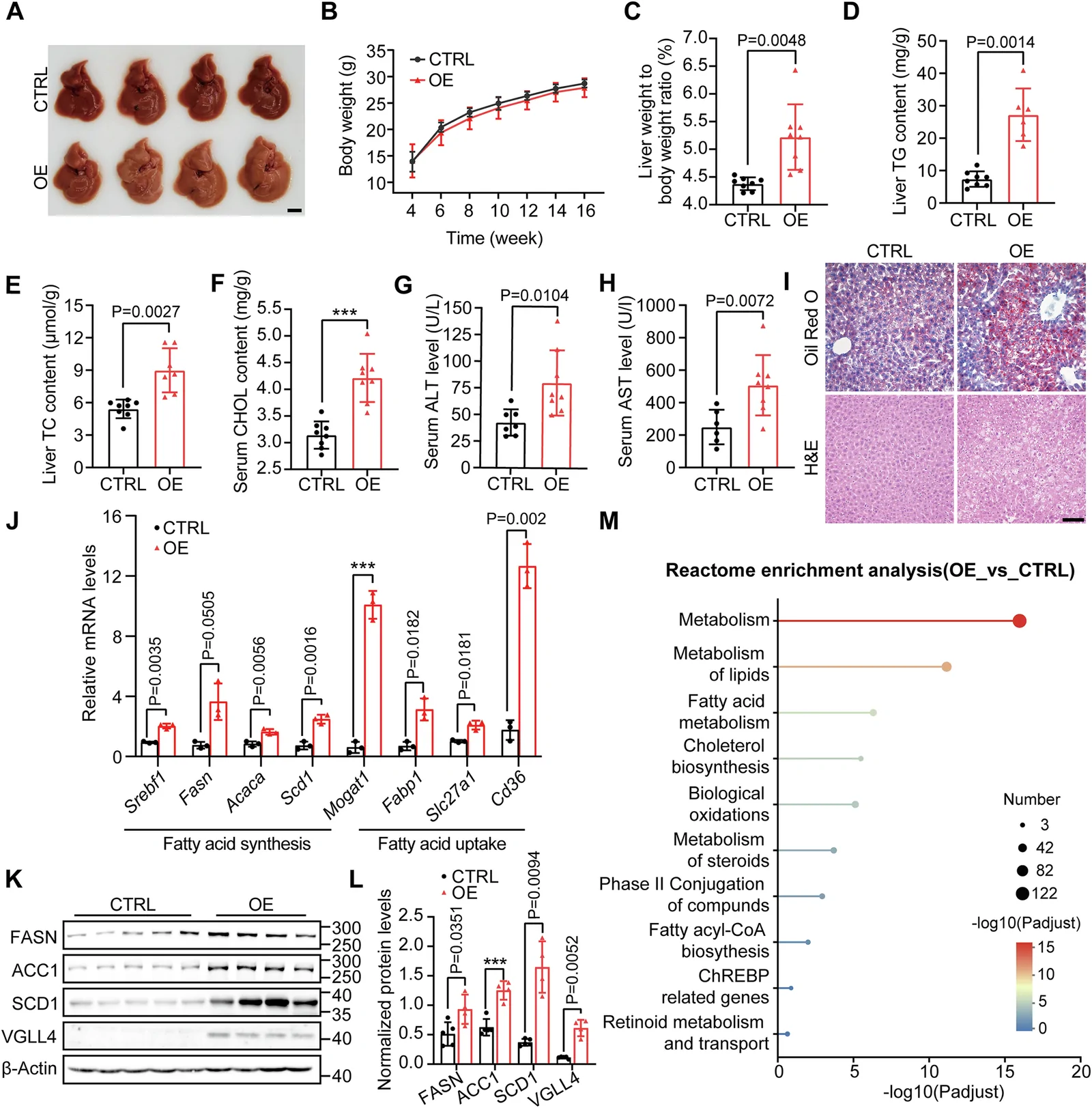
Hepatocyte-specific *Vgll4* overexpression induces hepatic steatosis. (**A**) Representative images of the livers (*n* = 4). Scale bar, 1 mm. (**B**) Body weight gain curves of mice in the indicated groups (*n* = 6–8). (**C**) Liver-to-body weight ratio of mice from the indicated groups (*n* = 8). (**D**–**F**) Liver TG (**D**), TC (**E**) and serum CHOL levels (**F**) from the indicated groups (*n* = 6–8). (**G**, **H**) Serum ALT (**G**) and AST (**H**) levels from the indicated groups (*n* = 6–8). (**I**) Representative images of Oil Red O-stained (upper panel) and H&E-stained (lower panel) liver sections (*n* = 5). Scale bar, 100 µm. (**J**) Relative mRNA levels of genes involved in fatty acid synthesis and uptake, with expression levels normalized to those of β-actin (*n* = 3). (**K**, **L**) Western blot analysis of lipogenic proteins (**K**) and normalized protein levels (**L**) from the indicated groups (*n* = 4–5). Protein levels were normalized to those of β-actin. Samples were derived from the same experiment and blots were processed in parallel. (**M**) Reactome enrichment analysis of differentially expressed genes between the indicated groups (*n* = 4). Data information: Data are presented as mean ± SD. Biological replicates are shown in (**B**–**H**, **J**). For (**B**), significance was determined by two-way ANOVA. For (**C**–**H**, **J**, **L**), significance was determined by Welch’s *t* test. For (**M**), enrichment significance was assessed using a hypergeometric test. ***P < 0.001, ns. indicates no significant difference. OE hepatocyte-specific *Vgll4* overexpression, CHOL cholesterol, TG triglyceride, TC total cholesterol, AST aspartate aminotransferase, ALT alanine aminotransferase, H&E hematoxylin and eosin, Srebf1 sterol regulatory element binding transcription factor 1. Source data are available online for this figure.

Given VGLL4 upregulation in hepatocytes from MAFLD patients, we next sought to elucidate VGLL4’s in vivo functions in hepatocytes. We generated a hepatocyte-specific *Vgll4*-overexpressing (OE) mouse line by crossbreeding *LAP-tTA* mice with *TetO-Vgll4* mice (Appendix Fig. S1A) (Wang et al, 2022). The efficiency of *Vgll4* overexpression was verified by immunofluorescence staining (Appendix Fig. S1B). Gross examination revealed that untreated, *Vgll4*-overexpressing mice had paler livers compared to the reddish livers of *TetO-Vgll4* (CTRL) mice (Fig. 1A). While liver and body weights remained similar between groups (Appendix Fig. S1C; Fig. 1B), OE mice exhibited higher liver-to-body weight ratios (Fig. 1C), and more severe hepatic steatosis, with higher liver triglyceride (TG) and total cholesterol (TC) (Fig. 1D,E). Despite similar serum TG levels between groups (Appendix Fig. S1D), the exacerbation of lipid accumulation coincided with elevated serum cholesterol (CHOL) levels in *Vgll4*-overexpressing mice (Fig. 1F). Further, serum levels of alanine transaminase (ALT) and aspartate aminotransferase (AST), indicators of liver injury, were increased in OE mice relative to controls (Fig. 1G,H). Consistent with these biochemical observations, Oil Red O and hematoxylin-eosin (H&E) staining visually confirmed that hepatic tissues from OE mice contained both larger and more numerous lipid droplets compared to controls (Fig. 1I). *Vgll4* overexpression significantly elevated the expression of key lipogenic and lipid uptake genes, as confirmed by reverse transcription quantitative real-time polymerase chain reaction (RT-qPCR) (Fig. 1J). Meanwhile, Western blot analysis (WB) showed de novo lipogenesis proteins, fatty acid synthase (FASN), acetyl-CoA carboxylase 1 (ACC1), and stearoyl-CoA desaturase 1 (SCD1), upregulated in OE mice liver (Fig. 1K,L). However, the overexpression of *Vgll4* also led to increased fatty acid β-oxidation gene expression versus controls, which may be attributed to a compensatory metabolic adaptation (Appendix Fig. S1E) (Chakravarthy et al, 2005). Additionally, Reactome enrichment analysis comparing OE and CTRL mice indicated that *Vgll4* overexpression in hepatocytes broadly influenced metabolic pathways especially those related to lipids, fatty acid, and cholesterol metabolism, which matches the hepatic steatosis phenotype seen in these mice (Fig. 1M). Together, these findings suggest that hepatic *Vgll4* overexpression is sufficient to drive spontaneous hepatosteatosis.

### Hepatocyte-specific *Vgll4* overexpression induces glucose intolerance, insulin resistance and inflammation

**Figure 2:**
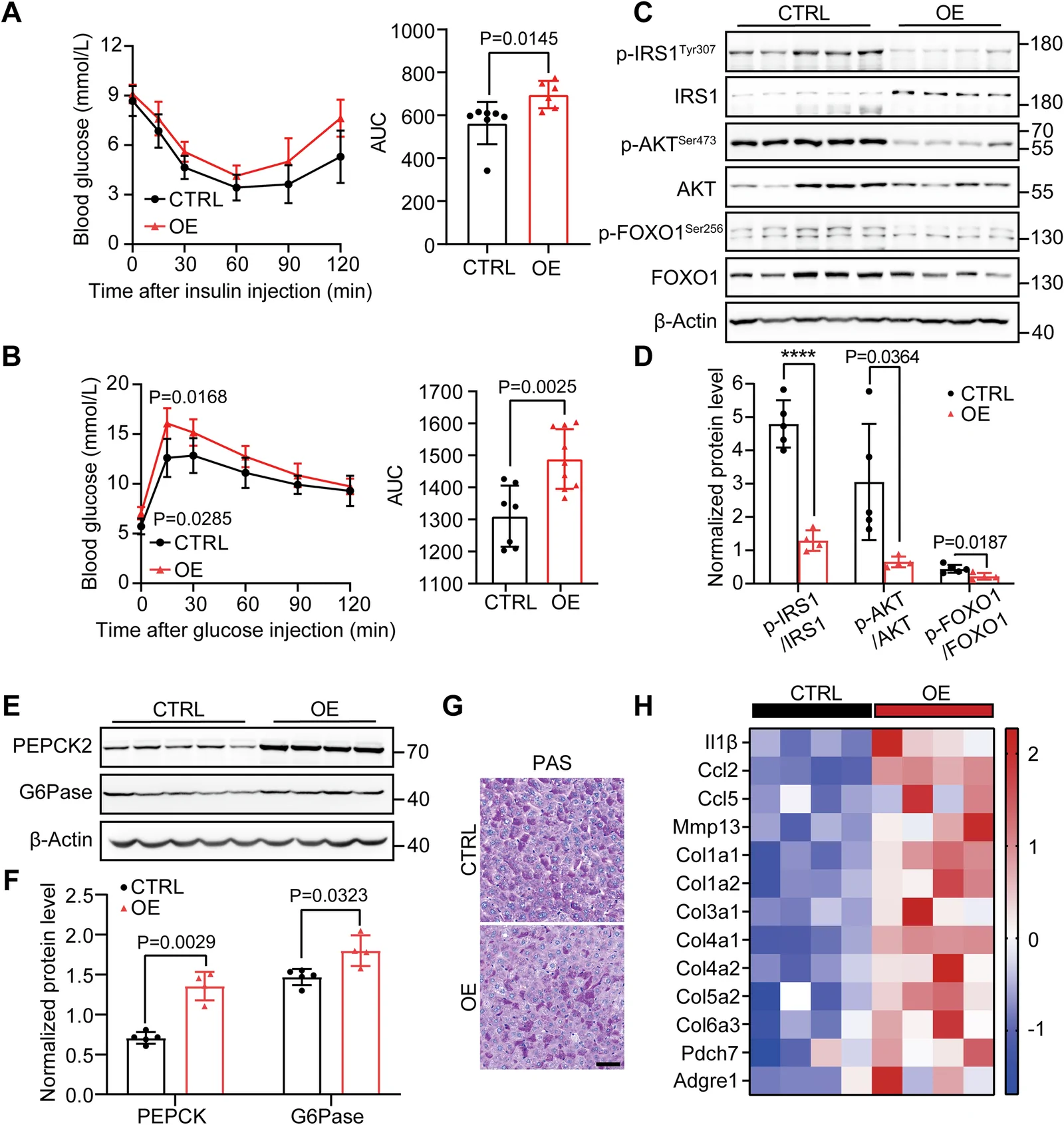
Hepatocyte-specific *Vgll4* overexpression impairs glucose homeostasis and induces insulin resistance and inflammation. (**A**, **B**) Insulin tolerance (ITT) and glucose tolerance tests (GTT) were performed in mice of the indicated groups and the AUC was calculated (*n* = 6–9). (**C**, **D**) Mice were intraperitoneally injected with insulin following 4 h fasting. Activation of the insulin signaling pathway in the livers of mice from the indicated groups was detected by western blotting (**C**); Protein levels of insulin signaling-related molecules were normalized to β-actin (**D**) (*n* = 4–5). Samples were derived from the same experiment and blots were processed in parallel. (**E**, **F**) Western blot analysis of protein levels of glucose 6-phosphatase and phosphoenolpyruvate carboxykinase in the livers of mice from the indicated groups (**E**), and the protein levels were normalized to β-actin (**F**) (*n* = 4–5). Samples were derived from the same experiment and blots were processed in parallel. (**G**) Representative images of periodic acid-Schiff-stained liver sections from mice of the indicated groups (*n* = 4). Scale bar, 50 µm. (**H**) Heatmap illustrates the expression profiles of genes associated with hepatic fibrosis and inflammation in the CTRL and OE mice, as determined by RT-qPCR (*n* = 4). Data information: Data are presented as mean ± SD. Biological replicates are shown in (**A**, **B**, **H**). For (**A**, **B**), significance was determined by two-way ANOVA. For (**D**, **F**) and AUCs in (**A**, **B**), significance was determined by Welch’s *t* test. ****P < 0.0001 as indicated. IR insulin resistance, IRS1 insulin receptor substrate 1, AUC area under the curve, G6Pase glucose 6-phosphatase, PEPCK2 phosphoenolpyruvate carboxykinase 2, PAS periodic acid-Schiff. Source data are available online for this figure.

Hepatic steatosis-associated lipotoxicity is well known to impair insulin signaling and reduce glucose tolerance, which may, in turn, further promote hepatic lipid accumulation and disease progression (Loomba et al, 2021). The insulin signaling pathway, which orchestrates glucose metabolism via sequential activation of key molecules including insulin receptor substrate 1 (IRS1), protein kinase B (AKT), and forkhead box protein O1 (FOXO1) downstream of insulin receptor binding, is critical for maintaining glucose homeostasis. To determine whether VGLL4 contributes to this metabolic dysregulation, we next evaluated its impact on systemic glucose homeostasis in vivo. *Vgll4*-overexpressing mice manifested more pronounced glucose intolerance and insulin resistance, as evidenced by higher area under the curve (AUC) values in both the insulin tolerance test (Fig. 2A) and glucose tolerance test (Fig. 2B). Consistently, *Vgll4*-overexpressing livers exhibited lower phosphorylation levels of key components in the insulin signaling pathway (Fig. 2C,D). Moreover, livers from OE mice with irregular glucose metabolism displayed elevated protein levels of phosphoenolpyruvate carboxykinase 2 (PEPCK2) and glucose-6-phosphatase (G6Pase), key enzymes in gluconeogenesis (Fig. 2E,F), along with reduced glycogen content (Fig. 2G). The progression of metabolic fatty liver disease is commonly accompanied by disrupted hepatic immune homeostasis and increased immune cell infiltration. In *Vgll4*-overexpressing mice, RT-qPCR revealed elevated hepatic expression of inflammation- and fibrogenesis-related genes, and immunofluorescence staining demonstrated enhanced myeloid cell infiltration (Fig. 2H; Appendix Fig. S2A–C). In conclusion, hepatocyte-specific *Vgll4* overexpression induces glucose intolerance, insulin resistance, and impaired immune homeostasis.

### Adenovirus-mediated *Vgll4* overexpression in adult mice recapitulates hepatic steatosis and impairs insulin signaling

To address the possibility that early-life *Vgll4* overexpression in the *LAP-tTA; TetO-Vgll4* model may influence liver development and thereby contribute to hepatosteatosis, we further performed adenovirus (ADV)-mediated *Vgll4* overexpression in adult WT mice. Eight-week-old WT mice were intravenously injected with ADV-Vgll4 or the control virus ADV-NC every four weeks, and liver tissues were collected 2 months after the first injection (Appendix Fig. S3A). ADV-Vgll4 administration induced an obvious steatotic liver phenotype, as evidenced by paler liver appearance, increased Oil Red O staining, and histological lipid accumulation (Appendix Fig. S3B,C). Moreover, ADV-Vgll4 increased the expression of lipogenic and fatty acid uptake-related genes (Appendix Fig. S3D) and was accompanied by impaired hepatic insulin signaling (Appendix Fig. S3E,F). These results indicate that *Vgll4* overexpression initiated in adult mice recapitulates the key metabolic features observed in the constitutive *Vgll4* overexpression model, including hepatic lipid accumulation and impaired insulin signaling.

### Hepatic *Vgll4* deficiency reduces HFD-induced hepatic steatosis and impairs glucose metabolism

**Figure 3:**
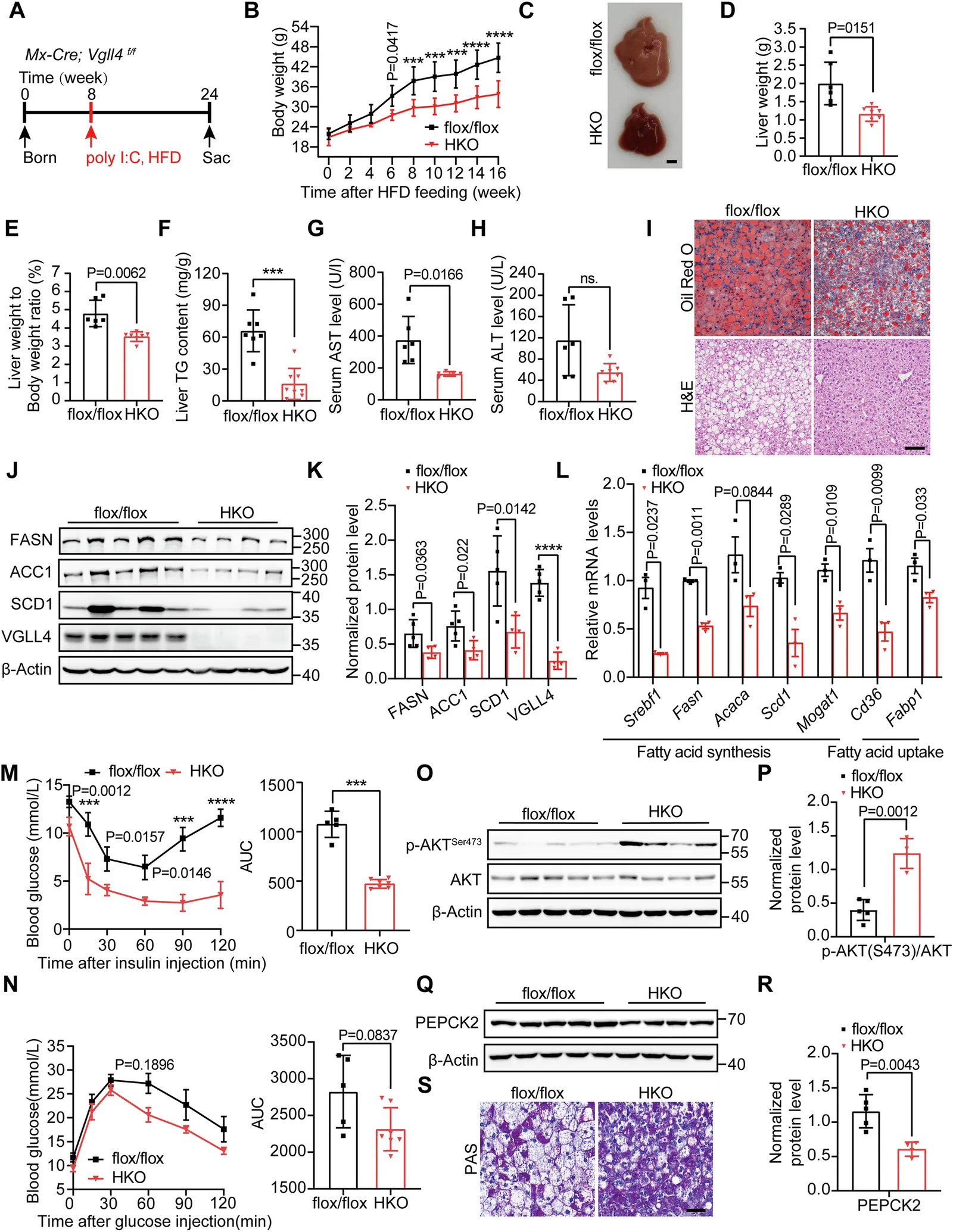
Hepatic *Vgll4* deletion attenuates HFD-induced hepatic steatosis. (**A**) Schematic diagram of animal experiments. The indicated mice were given an intraperitoneal injection of Poly I:C at 8 weeks of age, and all mice were fed a high-fat diet for 16 weeks. (**B**) Body weight gain curves of mice in the indicated groups (*n* = 5). (**C**) Representative images of the livers (*n* = 3). Scale bar, 5 mm. (**D**, **E**) Liver weight (**D**) and liver-to-body weight ratio (**E**) from the indicated groups (*n* = 6–7). (**F**) Liver TG content from the indicated groups (*n* = 7–8). (**G**, **H**) Serum AST (**G**) and ALT levels (**H**) from the indicated groups (*n* = 5–7). (**I**) Representative images of Oil Red O-stained (upper panel) and H&E-stained (lower panel) liver sections (*n* = 4). Scale bar, 100 µm. (**J**, **K**) Western blot analysis of lipogenic proteins (**J**) and the normalized protein levels (**K**). The protein levels were normalized to those of β-actin (*n* = 4–5). Samples were derived from the same experiment and blots were processed in parallel. (**L**) Relative mRNA levels of genes involved in fatty acid synthesis and uptake from the indicated groups, with expression levels normalized to those of β-actin (*n* = 3). (**M**, **N**) ITT and GTT were performed in mice of the indicated groups and the AUC was calculated (*n* = 5–7). (**O**, **P**) Western blot analysis of insulin signaling pathway activation in the livers of mice from the indicated groups (**O**). Protein levels were normalized to β-actin (**P**) (*n* = 4–5). Samples were derived from the same experiment and blots were processed in parallel. (**Q**, **R**) Western blot analysis of protein levels of phosphoenolpyruvate carboxykinase in the livers of mice from the indicated groups (**Q**), and the protein levels were normalized to β-actin (**R**) (*n* = 4–5). Samples were derived from the same experiment and blots were processed in parallel. (**S**) Representative images of periodic acid-Schiff-stained liver sections from mice of the indicated groups (*n* = 3). Scale bar, 50 µm. Data information: Data are presented as mean ± SD. Biological replicates are shown in (**B**, **D**–**H**, **L**–**N**). For (**B**, **M**, **N**), significance was determined by two-way ANOVA. For (**D**–**H**, **K**, **L**, **P**, **R**) and the AUCs in (**M**, **N**), significance was determined by Welch’s *t* test. ***P < 0.001, ****P < 0.0001, ns. indicates no significant difference. HKO hepatic *Vgll4* knockout, Poly I:C polyinosinic-polycytidylic acid sodium, HFD high-fat diet, TG triglyceride, IRS1 insulin receptor substrate 1, AUC area under the curve, PEPCK2 phosphoenolpyruvate carboxykinase 2, PAS periodic acid-Schiff. Source data are available online for this figure.

Since our study showed that *Vgll4* overexpression promotes hepatosteatosis, we next sought to determine whether hepatic *Vgll4* knockout (HKO) alleviates HFD-induced metabolic abnormalities. Eight-week-old *Vgll4*^*Mx1-cre*^ (HKO) and *Vgll4*^*f/f*^ (flox/flox) mice received intraperitoneal injections of polyinosinic-polycytidylic acid sodium (poly I:C), a synthetic double-stranded RNA agonist that triggers Cre recombinase-mediated *Vgll4* deletion, and were then fed an HFD for 16 weeks (Fig. 3A; Appendix Fig. S4A). Compared with control mice, HKO mice exhibited blunted weight gain, with a detectable difference emerging after 6 weeks of HFD feeding and progressively widening over the subsequent 10-week observation period (Fig. 3B). Consistent with this difference, gross examination revealed evident alterations in liver color and size between the two groups (Fig. 3C). Serum biochemical analysis further showed that *Vgll4*^*Mx1-cre*^ mice had markedly lower TG and CHOL levels than *Vgll4*^*f/f*^ mice (Appendix Fig. S4B,C). Similarly, HKO mice displayed reduced liver weight, liver-to-body weight ratio and hepatic TG content (Fig. 3D–F). In addition, *Vgll4* knockout attenuated HFD-induced liver injury, as indicated by lower serum AST levels, although serum ALT levels remained comparable between the two groups (Fig. 3G,H). Histological analyses further confirmed reduced hepatic lipid accumulation in HKO mice, as evidenced by Oil Red O and H&E staining (Fig. 3I). At the molecular level, Western blotting and RT-qPCR revealed decreased expression of molecules involved in lipogenesis and lipid uptake in HKO mice compared with controls under HFD feeding (Fig. 3J–L). Finally, knockout of *Vgll4* attenuated hepatic inflammation induced by hepatosteatosis (Appendix Fig. S4D). Collectively, hepatic *Vgll4* deficiency alleviates HFD-induced hepatic steatosis and inflammation.

Turning to glucose metabolism, *Vgll4* knockout significantly improved HFD-induced insulin resistance, as evidenced by a reduced AUC in the insulin tolerance test (Fig. 3M). HKO mice also showed a tendency toward improved glucose tolerance, although the GTT AUC did not reach statistical significance (Fig. 3N). Notably, the insulin-stimulated activation of downstream signaling cascades was restored in HKO mice under HFD feeding, as demonstrated by increased phosphorylation of AKT at the Ser473 residue compared to controls (Fig. 3O,P). Concomitantly, *Vgll4* deficiency resulted in suppressed gluconeogenesis and increased glycogen content under HFD feeding (Fig. 3Q–S). Together, these results suggest that hepatic *Vgll4* deletion alleviates HFD-induced insulin resistance and partially improves glucose metabolic dysfunction.

Given that Mx1-Cre-mediated recombination is not restricted to hepatocytes, we next used AAV2/8-TBG-Cre to examine the effect of hepatocyte-targeted *Vgll4* deletion under HFD feeding. Eight-week-old *Vgll4*^*f/f*^ mice were intravenously injected with AAV2/8-TBG-Cre (AAV-Cre) or AAV2/8-TBG-NC (AAV-NC), followed by HFD feeding for 16 weeks (Appendix Fig. S5A). AAV-Cre markedly reduced hepatic VGLL4 expression and produced protective effects consistent with those observed in the Mx1-Cre model, including attenuated hepatic lipid accumulation, decreased expression of lipogenic and fatty acid uptake-related genes, and increased AKT Ser473 phosphorylation (Appendix Fig. S5B–F). These findings from both the Mx1-Cre-based hepatic deletion model and the AAV2/8-TBG-Cre-mediated hepatocyte-targeted deletion model support a protective effect of *Vgll4* loss against HFD-induced hepatic steatosis and insulin signaling impairment.

### VGLL4 positively regulates PA/OA-induced hepatosteatosis in vitro

To determine the role of VGLL4 in human hepatocytes and provide key clues for elucidating its potential mechanisms in hepatic steatosis, we established a stable *VGLL4*-overexpressing L02 cell line (VGLL4-OE) by infecting cells with VGLL4-encoding lentivirus. In contrast, a *VGLL4*-knockdown L02 cell line (VGLL4-KD) was constructed using shRNA targeting *VGLL4*. L02 cells infected with empty-vector lentivirus (CON-OE) or shLuc-encoding lentivirus (CON-KD) served as corresponding controls. The robust *VGLL4* overexpression in the VGLL4-OE cell line and knockdown efficiency in the VGLL4-KD cell line were verified by Western blot (Appendix Fig. S6B–E). To mimic fatty acid-induced hepatic steatosis while limiting excessive palmitate-associated cytotoxicity, cells were treated with oleic acid (OA) and palmitic acid (PA) at a 2:1 molar ratio (Ricchi et al, 2009). Consistent with the results in mice, *VGLL4* overexpression enhanced PA/OA-induced lipid droplet accumulation, whereas *VGLL4* knockdown attenuated this accumulation, as evidenced by Oil Red O staining (Appendix Fig. S6A). Furthermore, BODIPY 558/568 C_12_ is a fluorescently labeled saturated fatty acid, which functions as a probe to assess fatty acid uptake and accumulation. Following a 20-h incubation with PA/OA, L02 cells were subsequently exposed to BODIPY 558/568 C_12_ for 4 h. Consequently, BODIPY 558/568 C_12_ internalization and fluorescence imaging demonstrated increased fatty acid uptake in VGLL4-OE L02 cells, while a marked reduction in uptake was observed in VGLL4-KD cells (Appendix Fig. S6A). At the protein level, the key lipogenic enzymes FASN, ACC1, and SCD1 were increased in VGLL4-OE cells, whereas their expression was reduced following VGLL4 knockdown (Appendix Fig. S6B–E). Consistent with these protein changes, transcripts associated with fatty acid synthesis and inflammatory responses were more abundant in VGLL4-OE cells, whereas the corresponding genes were suppressed in VGLL4-KD cells (Appendix Fig. S6F,G). Nonetheless, insulin-stimulated phosphorylation of AKT showed no differences between VGLL4-OE, VGLL4-KD, and their respective control groups, suggesting that VGLL4 may not directly cause insulin resistance (Appendix Fig. S6H,I). These findings collectively demonstrate that VGLL4 functions as a positive regulator of hepatosteatosis and inflammation within human hepatocytes.

### VGLL4 enhances TEAD/CEBPA cooperation to promote hepatosteatosis

**Figure EV2:**
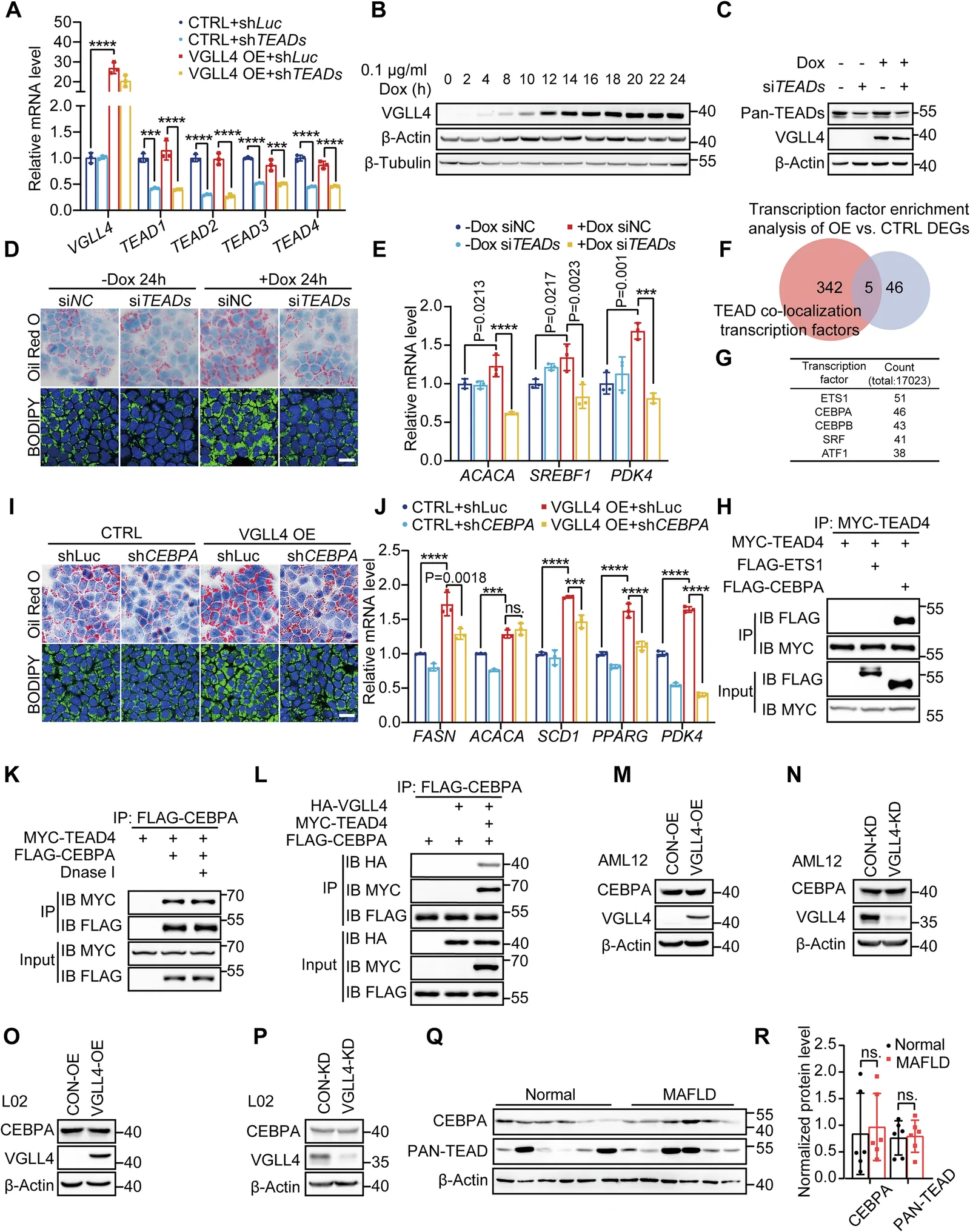
VGLL4 strengthens the TEAD4–CEBPA interaction in hepatosteatosis. (**A**) RT-qPCR analysis of *VGLL4* and *TEAD1–4* mRNA levels in control or *VGLL4*-overexpressing L02 cells transduced with sh*Luc* or sh*TEADs*, with expression levels normalized to those of β-actin (*n* = 3). (**B**) Western blot analysis showing Dox-induced VGLL4 expression in the inducible *VGLL4*-overexpressing L02 cell line. Cells were treated with 0.1 μg/mL Dox for the indicated times. Samples were derived from the same experiment and blots were processed in parallel. (**C**) Western blot validation of siRNA-mediated *TEADs* knockdown and Dox-induced VGLL4 expression in inducible *VGLL4*-overexpressing L02 cells treated with or without Dox. Samples were derived from the same experiment and blots were processed in parallel. (**D**) Representative images of Oil Red O-stained cells and BODIPY 558/568 C_12_-stained cells from the indicated groups after PA/OA treatment in the presence or absence of Dox (scale bar, 10 µm). (**E**) Relative mRNA levels of genes related to fatty acid metabolism and regulation in L02 cells from the indicated groups, with expression levels normalized to those of β-actin (*n* = 3). (**F**) Venn diagram of TEAD co-localizing transcription factors from ChIP-ATLAS and transcription factors enriched in *Vgll4*-overexpressing hepatocytes. (**G**) List of the intersection transcription factors from (**F**) and their regulating gene count in VGLL4-OE mice DEGs. (**H**) Co-immunoprecipitation analysis of the interactions between TEAD4 and ETS1 or CEBPA. (**I**) Representative images of Oil Red O-stained and BODIPY 558/568 C12-stained L02 cells from the indicated groups (scale bar, 10 µm). (**J**) Relative mRNA levels of genes related to fatty acid metabolism and regulation in L02 cells from the indicated groups, with expression levels normalized to those of β-actin (*n* = 3). (**K**) Co-immunoprecipitation assay to evaluate the interaction between CEBPA and TEAD4 in HEK293T cells, with or without DNase I treatment (to exclude DNA-mediated indirect binding). (**L**) Co-immunoprecipitation assay to examine the interaction between CEBPA and VGLL4 to determine whether the CEBPA–VGLL4 association depends on TEAD4 in HEK293T cells. (**M**–**P**) Western blot analysis of CEBPA protein levels in AML12 and L02 cells after *VGLL4/Vgll4* overexpression or knockdown. Samples were derived from the same experiment and blots were processed in parallel. (**Q**, **R**) Western blot analysis of CEBPA and TEAD protein levels in liver tissues from controls and patients with MAFLD (**Q**), with corresponding quantification normalized to β-actin (**R**) (*n* = 6). Samples were derived from the same experiment and blots were processed in parallel. Data information: Data are presented as mean ± SD. Representative technical replicates are shown in (**A**, **E**, **J**). For (**R**), significance was determined by Welch’s *t* test. For (**A**, **E**, **J**), significance was determined using one-way ANOVA. ***P < 0.001, ****P < 0.0001, ns. indicates no significant difference. Source data are available online for this figure

**Figure 4:**
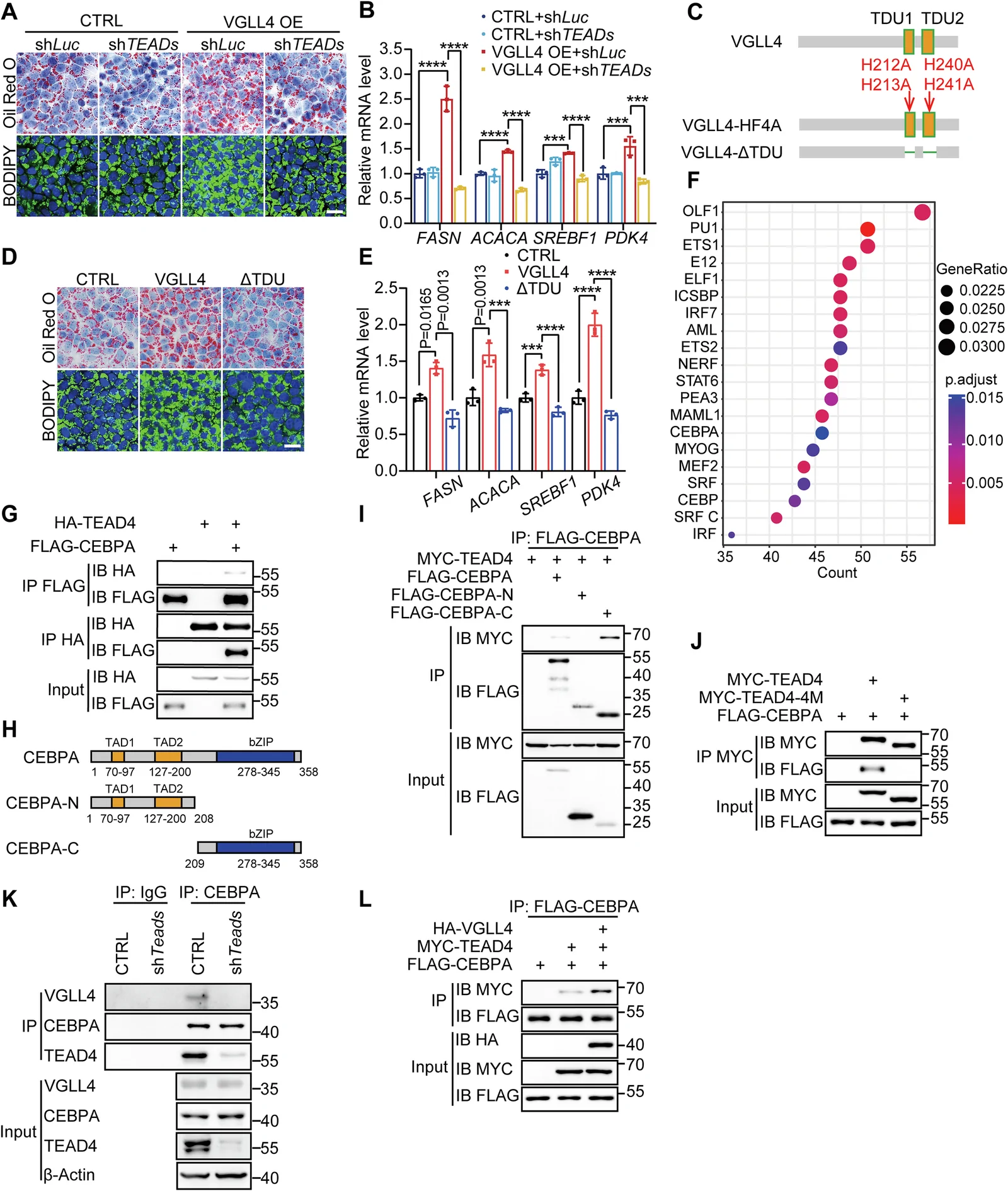
VGLL4 strengthens the TEAD–CEBPA interaction in hepatosteatosis. (**A**–**E**) Representative images of Oil Red O-stained and BODIPY 558/568 C12-stained L02 cells from the indicated groups (scale bar, 10 µm). Images in (**A**, **D**) are representative of at least three independent experiments. Relative mRNA levels of genes related to fatty acid metabolism and regulation in L02 cells from the indicated groups, with expression levels normalized to those of β-actin (**B**, **E**) (*n* = 3). Schematic illustration of the domain organization of human VGLL4, VGLL4-HF4A and VGLL4-ΔTDU (**C**). (**F**) Bubble plot illustrating the enrichment analysis of transcription factors (TFs) based on RNA-seq data (*n* = 4). (**G**) Co-immunoprecipitation analysis of the interaction between TEAD4 and CEBPA in HEK293T cells. (**H**) Schematic representation of full-length CEBPA and its truncation mutants. CEBPA-N (aa 1–208) retains the transactivation domains (TAD1 and TAD2), whereas CEBPA-C (aa 209–358) contains the bZIP DNA-binding domain. (**I**) Co-immunoprecipitation assays were conducted to investigate the interaction between TEAD4 and full-length CEBPA or its N- and C-terminal fragments. (**J**) Co-immunoprecipitation analysis of the interaction between CEBPA and TEAD4 or its mutant (TEAD4-4M) in HEK293T cells. (**K**) Endogenous CEBPA Co-IP assay in control and sh*TEADs* L02 cells. Samples were derived from the same experiment and blots were processed in parallel. (**L**) Co-immunoprecipitation assay to assess the impact of VGLL4 on the interaction between CEBPA and TEAD4 in HEK293T cells. Data information: Data are presented as mean ± SD. Representative technical replicates are shown in (**B**, **E**). For (**B**, **E**), significance was determined using one-way ANOVA. For (**F**), enrichment significance was assessed using a hypergeometric test. ***P < 0.001, ****P < 0.0001 as indicated. IP immunoprecipitation, IB immunoblotting, DNase I deoxyribonuclease I. Source data are available online for this figure.

Having observed that VGLL4 expression tightly regulates hepatosteatosis both in vivo and in vitro, we next investigated the underlying mechanism. Since VGLL4 lacks a DNA-binding domain, we first examined the requirement for TEAD family members, which are well-established transcription partners of VGLL4, in this process. A *TEADs*-knockdown L02 cell line overexpressing *VGLL4* was established (Fig. EV2A), and Oil Red O staining, BODIPY 558/568 C_12_ uptake, and RT-qPCR analyses revealed that *TEADs* knockdown markedly attenuated PA/OA-induced lipid accumulation in *VGLL4*-overexpressing cells (Fig. 4A,B). To minimize potential metabolic adaptation caused by long-term stable *VGLL4* overexpression, we generated a Dox-inducible *VGLL4*-overexpressing L02 cell line (Fig. EV2B). Consistent with the stable overexpression system, short-term Dox-induced VGLL4 expression promoted PA/OA-induced lipid droplet accumulation, fatty acid uptake, and lipid metabolism-related gene expression, whereas *TEADs* knockdown largely attenuated these effects (Fig. EV2C–E). These results further demonstrate that the pro-steatotic function of VGLL4 depends on TEADs. Having confirmed the necessity of TEADs for VGLL4-induced lipid accumulation, we next examined whether their physical interaction is indispensable for this process. Previous studies have shown that VGLL4 interacts with TEADs via its two TDU domains, and the deletion of TDU domains disrupts the interaction (Fig. 4C) (Feng et al, 2019). To further validate this requirement, we established an L02 cell line stably expressing a VGLL4 variant lacking the TDU domains (VGLL4-ΔTDU). In contrast to full-length VGLL4, VGLL4-ΔTDU failed to promote lipid uptake or induce lipid droplet accumulation (Fig. 4D,E). Collectively, these results demonstrate that the pro-steatotic activity of VGLL4 in hepatocytes critically depends on its TDU domain-mediated interaction with TEADs.

Having established that TEADs are required for VGLL4-induced lipid accumulation, we next asked whether hepatocyte-enriched transcription factors may cooperate with VGLL4–TEADs to direct this metabolic program. We therefore sought to identify candidate factors that were both enriched in VGLL4-regulated transcriptional programs during hepatic steatosis and associated with TEADs in transcriptional regulatory complexes. Transcriptome profiling of the *LAP-tTA; TetO-Vgll4* and *TetO-Vgll4* mice hepatocytes was subjected to transcription factor enrichment analysis. The enriched transcription factors were grouped into immune (e.g., PU1, IRF7, STAT6), development (e.g., MYOG, MEF2) and metabolism (e.g., ETS1, CEBPA) categories (Fig. 4F). Meanwhile, we analyzed the potential transcription factor partners that bind to the TEAD family using the ChIP-ATLAS database (https://chip-atlas.org/) (Dataset EV1). The intersection of enriched factors from *Vgll4*-overexpressing hepatocytes and TEADs ChIP-seq partners yielded five transcription factors as common candidates (Fig. EV2F,G). Among them, ETS1 showed no interaction with TEAD4 (Fig. EV2H), whereas CEBPA ranked highly and showed a robust interaction with TEAD4 (Figs. 4G and  EV2H). To validate its functional relevance, we established a *CEBPA*-knockdown L02 cell line overexpressing *VGLL4*. Oil Red O staining and BODIPY 558/568 C_12_ uptake assays revealed that *CEBPA* depletion markedly reduced PA/OA-induced lipid accumulation in *VGLL4*-overexpressing cells (Fig. EV2I), a finding corroborated by molecular analyses (Fig. EV2J).

Having identified both TEADs and CEBPA as critical mediators of VGLL4-induced hepatic steatosis, we next mapped their binding domains. CEBPA contains two N-terminal transactivation domains (TAD1 and TAD2) and a C-terminal basic leucine zipper (bZIP) domain (Fig. 4H) (Behdad et al, 2015). Based on this domain architecture, we generated N-terminal and C-terminal truncation mutants of CEBPA (Fig. 4H). Truncation analysis indicated that the C-terminal region of CEBPA is required for this interaction (Fig. 4I), whereas mutation of TEAD4 residues R95, K96, S100, and Q103 (TEAD4-4M) abolished binding (Fig. 4J). Although TEAD4 residue Q103 is known to be involved in DNA binding, the TEAD4–CEBPA interaction was DNA-independent, as it was unaffected by DNase I treatment (Fig. EV2K). Importantly, VGLL4 did not bind CEBPA directly; instead, TEAD4 functioned as a scaffold bridging VGLL4 and CEBPA, as confirmed by endogenous and exogenous pull-down experiments (Figs. 4K and  EV2L). Furthermore, Co-IP results demonstrated that the CEBPA–TEAD4 interaction was enhanced in the presence of VGLL4 (Fig. 4L). This enhancement was not attributable to increased CEBPA abundance, as *Vgll4/VGLL4* overexpression or knockdown did not noticeably alter CEBPA protein levels in AML12 or L02 hepatocytes (Fig. EV2M–P). Moreover, TEADs and CEBPA protein levels were not significantly altered in MAFLD patient liver tissues compared with normal controls (Fig. EV2Q,R), supporting an interaction-based rather than expression-based mechanism. Collectively, these findings support a model in which VGLL4 associates with TEADs and strengthens their interaction with CEBPA, thereby promoting hepatic steatosis.

### VGLL4 promotes *Pdk4* transcription through TEAD4 and CEBPA

**Figure EV3:**
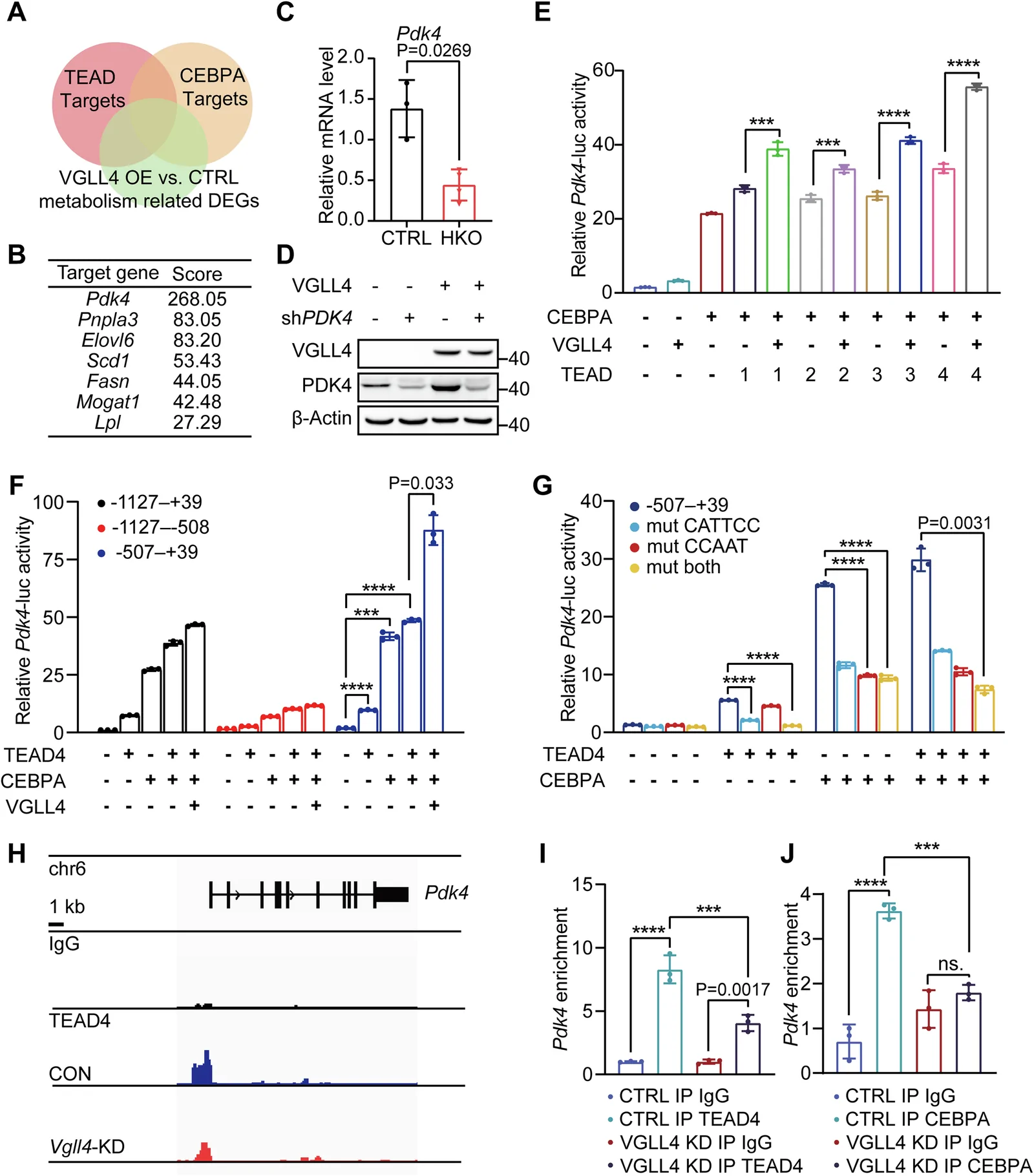
VGLL4 promotes *Pdk4* transcription through TEAD4 and CEBPA. (**A**) Venn diagram illustrating the strategy for selecting genes downstream of VGLL4-dependent TEAD/CEBPA regulation. (**B**) ChIP-ATLAS binding scores for candidate metabolic genes identified among DEGs in VGLL4-OE mice. Among the selected candidates, *Pdk4* showed the highest score, supporting its prioritization as a candidate gene with strong TEAD-binding evidence. (**C**) Relative mRNA levels of *Pdk4* in *Vgll4*-knockout and the corresponding control mice, with expression levels normalized to those of β-actin (*n* = 3–4). (**D**) Validation of the constructed L02 stable cell line. Samples were derived from the same experiment and blots were processed in parallel. (**E**) Luciferase reporter assay analyzing the transcriptional activity of the *Pdk4* promoter in L02 cells co-transfected with CEBPA, VGLL4, and different isoforms of the TEAD family (*n* = 3). (**F**) Luciferase reporter assays using serially truncated *Pdk4* promoter constructs in cells co-transfected with the indicated combinations of TEAD4, CEBPA, and VGLL4 (*n *= 3). (**G**) Luciferase reporter assays using the −507 to +39 *Pdk4* promoter reporter carrying point mutations in the predicted TEAD4-binding motif CATTCC, the predicted CEBPA-binding motif CCAAT, or both motifs (*n *= 3). (**H**) TEAD4 CUT&Tag tracks at the *Pdk4* locus in control and *Vgll4*-knockdown cells. (**I**, **J**) ChIP-qPCR validation of TEAD4 (**I**) and CEBPA (**J**) enrichment at the *Pdk4* promoter after *Vgll4* knockdown (*n* = 3). Data information: Data are presented as mean ± SD, representative technical replicates are shown in (**I**, **J**). Biological replicates are shown in (**C**, **E**–**G**). For (**C**), significance was determined by Welch’s *t* test. For (**E**, **I**, **J**), significance was determined using one-way ANOVA. For (**F**, **G**), significance was determined using two-way ANOVA. ***P < 0.001, ****P < 0.0001, ns. indicates no significant difference. Source data are available online for this figure

**Figure 5:**
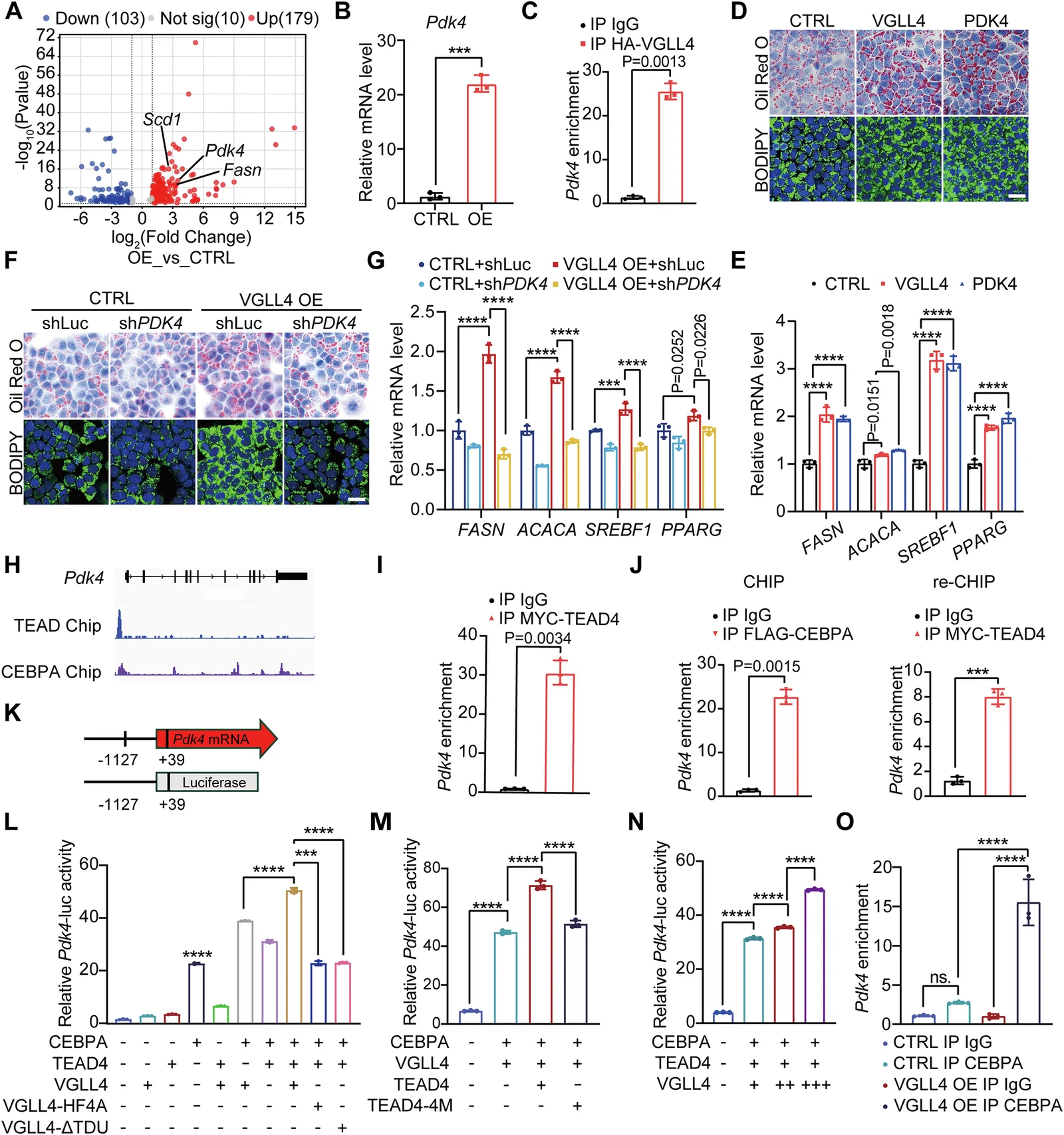
VGLL4 promotes *Pdk4* transcription through TEAD4 and CEBPA. (**A**) Volcano plot illustrating differentially expressed genes in metabolic pathways in the comparison between *Vgll4* overexpression (OE) and control (CTRL) groups (*n* = 4). (**B**) Relative *Pdk4* mRNA levels in CTRL and VGLL4 OE mice. Relative mRNA levels were normalized to β-actin (*n* = 3). (**C**) ChIP-qPCR analysis of VGLL4 enrichment at the *Pdk4* promoter in AML12 cells (*n* = 3). (**D**–**G**) Representative images of Oil Red O-stained and BODIPY 558/568 C12-stained L02 cells from the indicated groups (scale bar, 10 µm). Data shown are representative of at least three independent experiments (**D**, **F**). Relative mRNA levels of genes related to fatty acid metabolism and regulation in L02 cells from the indicated groups, with expression levels normalized to those of β-actin (**E**, **G**) (*n* = 3). (**H**) ChIP-seq tracks showing TEAD4 (GSM3019324) and CEBPA (GSM4066438) binding peaks at the *Pdk4* promoter. (**I**) ChIP-qPCR analysis of TEAD4 enrichment at the *Pdk4* promoter in AML12 cells (*n* = 3). (**J**) Sequential ChIP (re-ChIP)-qPCR analysis of CEBPA and TEAD4 co-occupancy at the *Pdk4* promoter in AML12 cells (*n* = 3). (**K**) Schematic of the Pdk4 promoter-driven luciferase reporter construct. (**L**, **M**) Luciferase reporter activity driven by *Pdk4* promoter in L02 cells transfected with indicated plasmids (*n* = 3). (**N**) Luciferase reporter assay analyzing the transcriptional activity of the *Pdk4* promoter in L02 cells co-transfected with CEBPA, TEAD4, and graded levels of VGLL4 (*n* = 3). (**O**) ChIP-qPCR analysis of CEBPA occupancy at the *Pdk4* promoter in control and Vgll4-overexpressing AML12 cells (*n* = 3). Data information: Data are presented as mean ± SD. Biological replicates are shown in (**B**, **L**–**N**). Representative technical replicates are shown in (**C**, **E**, **G**, **I**, **J**, **O**). For (**B**, **C**, **I**, **J**), significance was determined by Welch’s *t* test. For (**E**, **G**, **L**–**O**), significance was determined using one-way ANOVA. For (**A**), differential expression was assessed using the Wald test implemented in DESeq2, with *P* values adjusted using the Benjamini–Hochberg method. ***P < 0.001, ****P < 0.0001, ns. indicates no significant difference. ChIP chromatin immunoprecipitation, IP immunoprecipitation, Luc luciferase. Source data are available online for this figure.

Having established that TEADs and CEBPA mediate the pro-steatotic effects of VGLL4, we next sought to identify a functionally relevant downstream effector. As shown previously, transcriptomic analysis of *Vgll4*-overexpressing versus control mouse livers revealed a distinct set of differentially expressed genes (DEGs), with Reactome enrichment highlighting a strong association with metabolic pathways (Fig. 1M). To pinpoint metabolically relevant genes downstream of this regulatory pathway, we prioritized genes by integrating multiple datasets (Fig. EV3A): (1) DEGs enriched in metabolic pathways from VGLL4-OE RNA-seq (Fig. 5A), (2) TEADs binding scores from ChIP-ATLAS (https://chip-atlas.org/) (Fig. EV3B), and (3) known CEBP family member target genes reported in the literature (Attia et al, 2011). This intersection analysis highlighted PDK4 as a strong candidate. RT-qPCR validation confirmed that *Pdk4* expression was consistently increased by *Vgll4* overexpression and decreased by *Vgll4* knockout in mice (Figs. 5B and  EV3C). Additionally, in vitro, knockdown of either *TEADs* or *CEBPA* in the *VGLL4*-overexpressing cell line led to a significant reduction in *PDK4* mRNA levels, implying that PDK4 expression is regulated by VGLL4, TEADs, and CEBPA (Figs. 4B and  EV2J). Chromatin immunoprecipitation (ChIP) assays consistently demonstrated the occupancy of VGLL4 at the *Pdk4* promoter, suggesting its participation in the transcriptional regulation of *Pdk4* during hepatic steatosis (Fig. 5C). Functionally, lentivirus-mediated *PDK4* overexpression in L02 cells enhanced lipid droplet accumulation, increased lipid uptake under PA/OA treatment, and upregulated lipogenic genes (Fig. 5D,E). The lipid accumulation caused by *VGLL4* overexpression was markedly attenuated after *PDK4* knockdown (Figs. 5F,G and  EV3D), further confirming that PDK4 acts as a downstream effector of VGLL4 in hepatosteatosis.

We next investigated how VGLL4 influences TEAD4- and CEBPA-dependent *Pdk4* expression to promote lipid accumulation in hepatocytes. ChIP-seq data suggested that TEAD family members and CEBPA showed binding peaks within the *Pdk4* promoter region (Fig. 5H). Subsequently, ChIP and re-ChIP experiments supported the co-occupancy of TEAD and CEBPA at the *Pdk4* promoter in hepatocytes (Fig. 5I,J). To determine whether VGLL4 regulates *Pdk4* transcription by modulating the cooperative activity of TEAD4 and CEBPA at the *Pdk4* promoter, we cloned the CEBPA/TEAD binding site from the *Pdk4* promoter and fused it to the luciferase reporter vector (Fig. 5K). Since VGLL4 interacts with multiple TEAD family members in various contexts, we first used the *Pdk4* promoter luciferase reporter to compare the contribution of different TEAD isoforms. In the presence of CEBPA and VGLL4, TEAD4 produced the strongest activation of the *Pdk4* promoter among TEAD1–4 (Fig. EV3E), supporting TEAD4 as the predominant TEAD isoform in this regulatory context.

Given that TEAD4 emerged as the predominant isoform, we focused subsequent mechanistic studies on TEAD4. Individual or pairwise overexpression of VGLL4, TEAD4, and CEBPA only modestly increased reporter activity, whereas co-expression of all three factors markedly enhanced *Pdk4* promoter activity (Fig. 5L). A VGLL4 mutant bearing mutations at residues H212A, H213A, H240A, and H241A (VGLL4-HF4A), another TEAD-binding-defective mutant analogous to VGLL4-ΔTDU, was used to further test the requirement of the VGLL4–TEAD4 interaction (Fig. 4C). Both VGLL4-HF4A and VGLL4-ΔTDU failed to further activate the reporter in the presence of TEAD4 and CEBPA, underscoring the importance of the VGLL4-TEAD4 interaction (Fig. 5L). Likewise, TEAD4-4M, which abrogates CEBPA binding, resulted in the loss of its capacity to further activate the *Pdk4* luciferase reporter (Fig. 5M).

To further define the promoter region underlying coordinated activation of *Pdk4*, we generated a series of *Pdk4* promoter truncation luciferase reporters. These assays narrowed the major responsive region to the -507 to +39 fragment of the *Pdk4* promoter (Fig. EV3F). JASPAR analysis identified putative TEAD4 and CEBPA motifs within this region, CATTCC at -507 to -502 and CCAAT at -241 to -237, respectively. Mutation of either motif reduced the corresponding TEAD4- or CEBPA-mediated promoter activation, while mutation of both motifs further impaired reporter activity (Fig. EV3G). These results indicate that the predicted TEAD4- and CEBPA-responsive motifs functionally contribute to *Pdk4* promoter activation, although the residual promoter activity after mutation suggests that additional TEAD4- or CEBPA-responsive elements may also exist within this region.

Having functionally validated the predicted TEAD4- and CEBPA-responsive motifs, we next examined whether VGLL4 affects TEAD4 and CEBPA occupancy at the endogenous *Pdk4* locus. Genome-wide peak calling of the TEAD4 CUT&Tag data identified 11,394 peaks in control AML12 cells and 9,068 peaks following *Vgll4* knockdown. A *Pdk4*-associated peak was detected in both conditions. Spike-in-normalized TEAD4 CUT&Tag analysis showed that TEAD4 enrichment at the *Pdk4* regulatory region was clearly above the IgG background, whereas a lower normalized TEAD4 signal was observed following *Vgll4* knockdown (Fig. EV3H). Consistently, ChIP-qPCR further confirmed that VGLL4 depletion decreased the enrichment of both TEAD4 and CEBPA at the *Pdk4* promoter (Fig. EV3I,J).

Complementing these loss-of-function data, *VGLL4* overexpression enhanced *Pdk4* promoter activity in a TEAD4- and CEBPA-dependent manner and increased CEBPA occupancy at the *Pdk4* promoter, as shown by luciferase reporter assays and ChIP-qPCR (Fig. 5N,O). Together with the Co-IP results showing that VGLL4 strengthens the CEBPA-TEAD4 interaction (Fig. 4L), these data support a model in which VGLL4 facilitates TEAD4 and CEBPA occupancy at the *Pdk4* promoter, thereby enhancing *Pdk4* transcription. Taken together, these findings support a model in which VGLL4 promotes hepatocellular lipid accumulation by enhancing TEAD4–CEBPA co-occupancy and cooperative activation of the *Pdk4* promoter.

### YAP antagonizes VGLL4-dependent *Pdk4* transcription

**Figure EV4:**
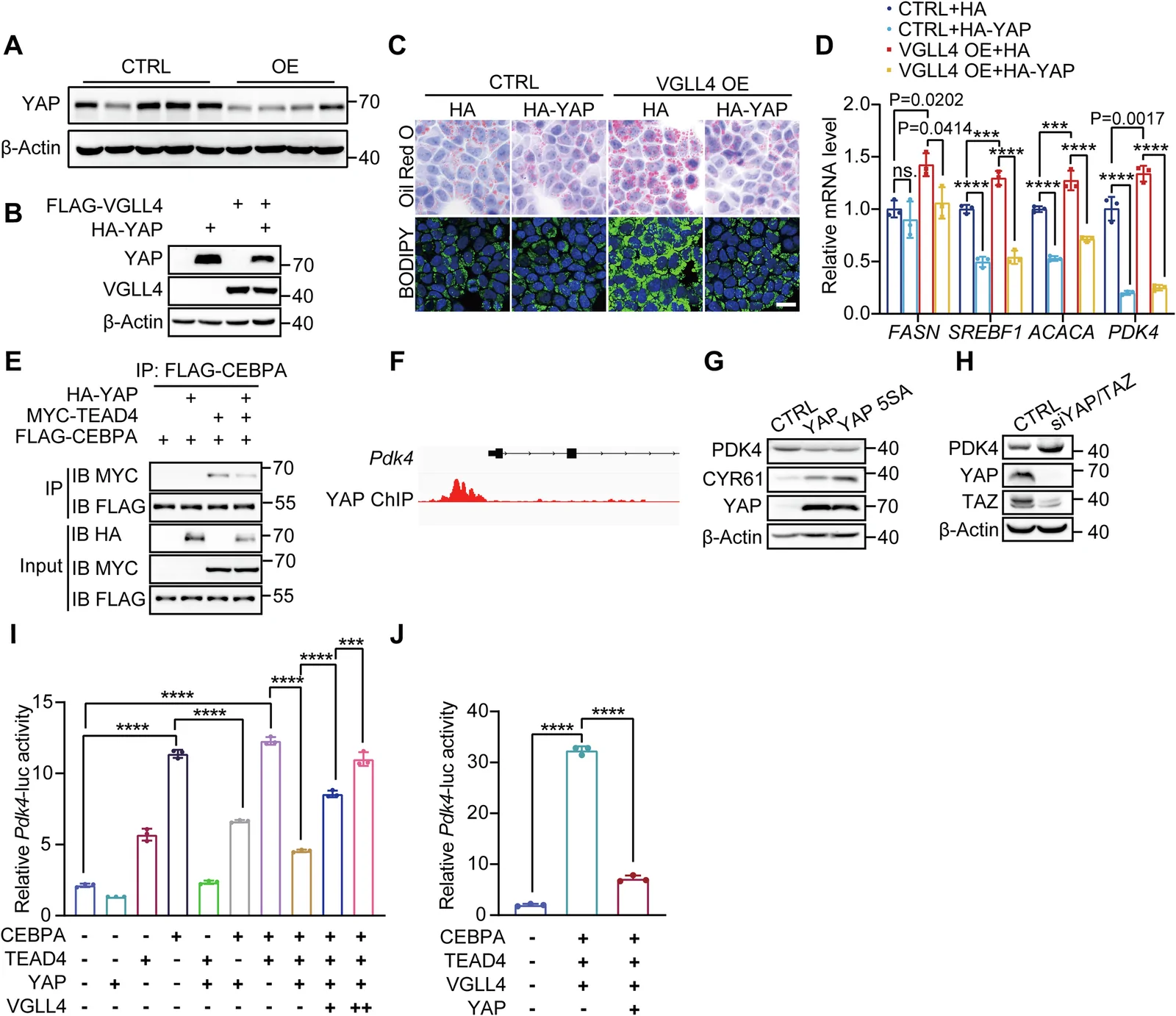
VGLL4-dependent regulation of *Pdk4* by TEAD and CEBPA is antagonized by YAP. (**A**) Western blot detection of YAP in CTRL and VGLL4-OE mice (*n* = 4–5). Samples were derived from the same experiment and blots were processed in parallel. (**B**) Validation of the constructed L02 stable cell line. Samples were derived from the same experiment and blots were processed in parallel. (**C**) Representative images of Oil Red O-stained and BODIPY 558/568 C12-stained L02 cells from the indicated groups (scale bar, 10 µm). (**D**) Relative mRNA levels of genes related to fatty acid metabolism and regulation in L02 cells from the indicated groups, with expression levels normalized to those of β-actin (*n* = 3). (**E**) Co-IP assay to assess the impact of YAP on the interaction between CEBPA and TEAD4 in HEK293T cells. (**F**) ChIP-seq data showing a YAP binding peak (GSM1331248) at the *Pdk4* promoter. (**G**, **H**) Western blot analysis of PDK4 and known YAP target proteins in YAP- or YAP-5SA-overexpressing and YAP/TAZ-knockdown L02 cells. Samples were derived from the same experiment and blots were processed in parallel. (**I**, **J**) Luciferase reporter assays were performed to analyze the transcriptional activity of the *Pdk4* promoter in L02 cells co-transfected with CEBPA, TEAD4, YAP and VGLL4 (*n* = 3). Data information: Data are presented as mean ± SD. Biological replicates are shown in (**I**, **J**). Representative technical replicates are shown in (**D**). For (**D**, **I**, **J**), significance was determined using one-way ANOVA. ***P < 0.001, ****P < 0.0001, ns. indicates no significant difference. Source data are available online for this figure

Given the known competition between VGLL4 and YAP for TEAD4 binding, we next examined whether YAP modulates this transcriptional mechanism during hepatic steatosis. To address this, we first examined YAP protein levels in VGLL4-OE mouse livers and observed a significant reduction compared with controls (Fig. EV4A). Given that *Vgll4* overexpression promotes hepatic lipid accumulation, this decrease prompted us to test whether YAP counteracts VGLL4-driven lipid metabolic remodeling. To directly examine the competitive dynamics between YAP and VGLL4 for TEAD4 binding in relation to lipid metabolism, we established stable L02 cell lines overexpressing *VGLL4* alone, *YAP* alone, or both (Fig. EV4B). Oil Red O staining and BODIPY 558/568 C_12_ uptake assays revealed that YAP co-overexpression significantly attenuated lipid accumulation induced by *VGLL4* overexpression (Fig. EV4C). RT-qPCR analysis further supported these observations, showing that YAP co-overexpression mitigated the VGLL4-induced upregulation of hepatosteatosis-associated genes (Fig. EV4D). With evidence that YAP could partially counteract VGLL4’s pro-steatotic effects, we further investigated whether YAP influences this downstream transcriptional pathway. Co-IP experiments revealed that YAP weakened the interaction between CEBPA and TEAD4 (Fig. EV4E), consistent with disruption of the VGLL4-mediated enhancement of TEAD4–CEBPA association. Then, we investigated the regulatory role of YAP in *Pdk4* activity. Public ChIP-seq data (GSM1331248) showed YAP binding peaks within the *Pdk4* promoter region, indicating that YAP may regulate *Pdk4* at the transcriptional level (Fig. EV4F). Western blotting revealed that overexpression of YAP, including its constitutively active form (YAP-5SA), significantly downregulated PDK4 expression. Conversely, knockdown of YAP/TAZ (which exhibit functional redundancy) promoted PDK4 expression (Fig. EV4G,H). This suggests that YAP negatively regulates PDK4 expression, potentially counteracting the pro-steatotic effects of the VGLL4/TEAD4/CEBPA axis. Luciferase assays with the *Pdk4* promoter further demonstrated that YAP repressed CEBPA–TEAD4-mediated activation of the *Pdk4* promoter, while co-expression of VGLL4 reversed this repression (Fig. EV4I). Moreover, YAP attenuated *Pdk4* promoter activation induced by the combined expression of VGLL4, TEAD4, and CEBPA (Fig. EV4J). Collectively, these results suggest that YAP antagonizes VGLL4-dependent activation of *Pdk4* during hepatic steatosis, consistent with the well-established competition between YAP and VGLL4 for TEAD4 binding.

### *Pdk4* knockdown partially alleviates hepatosteatosis in *Vgll4*-overexpressing mice

**Figure 6:**
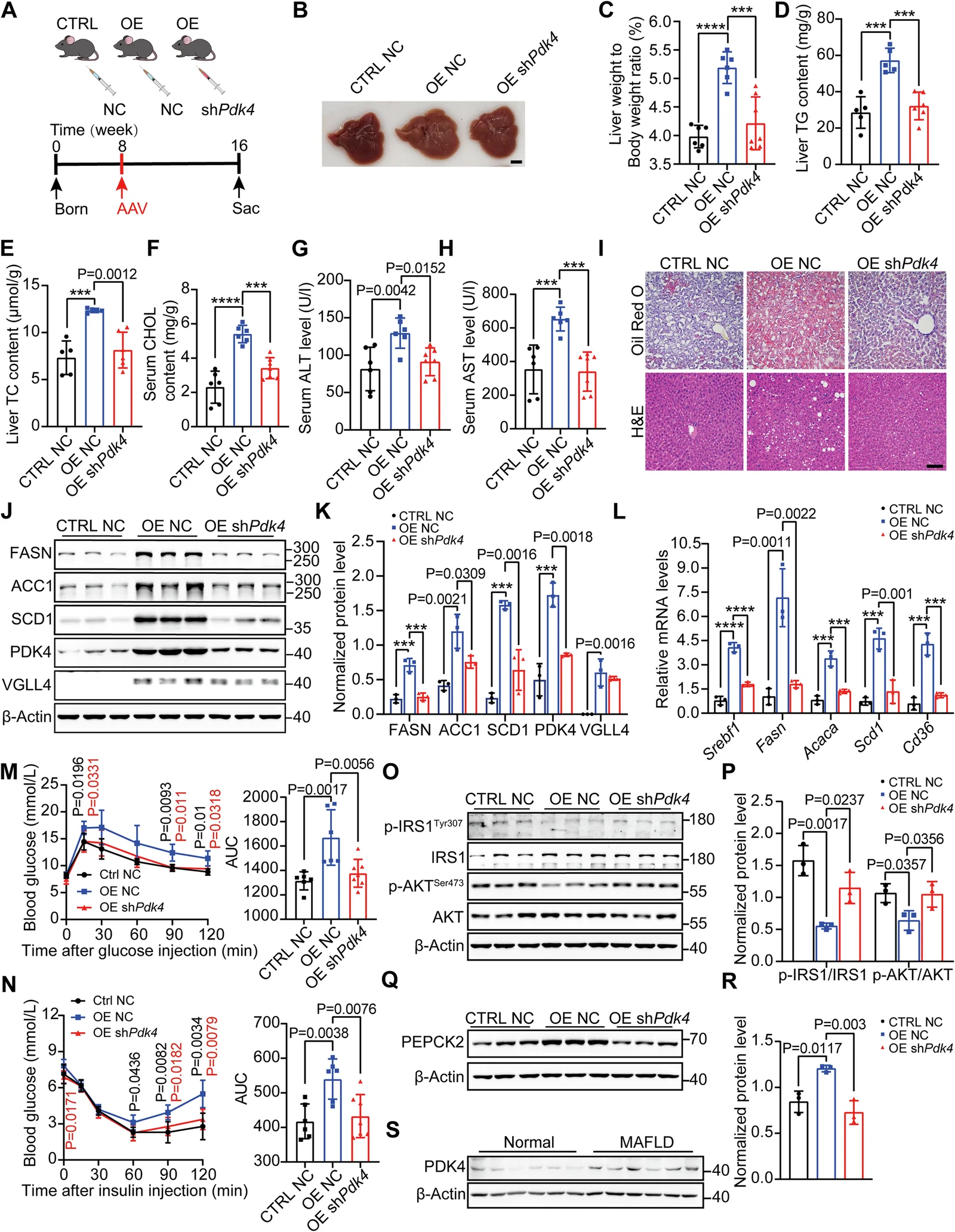
*Pdk4* knockdown partially alleviates hepatosteatosis in *Vgll4*-overexpressing mice. (**A**) Schematic diagram of animal experiments. OE mice were injected via the tail vein with AAV-sh*Pdk4* (OE-sh*Pdk4*) or the corresponding negative-control vector (OE-NC). CTRL mice received the negative-control vector, and metabolic phenotypes were subsequently assessed. (**B**) Representative images of the livers (*n* = 3). Scale bar, 5 mm. (**C**–**E**) Liver-to-body weight ratio (**C**), liver TG (**D**) and TC (**E**) from the indicated groups (*n* = 5–7). (**F**–**H**) Serum CHOL (**F**), ALT (**G**) and AST (**H**) from the indicated groups (*n* = 6–7). (**I**) Representative images of Oil Red O-stained (upper panel) and H&E-stained (lower panel) liver sections (*n* = 4). Scale bar, 100 µm. (**J**, **K**) Western blot analysis of lipogenic proteins (**J**) and normalized protein levels (**K**). The protein levels were normalized to those of β-actin (*n* = 3). Samples were derived from the same experiment and blots were processed in parallel. (**L**) Relative mRNA levels of genes involved in fatty acid synthesis and uptake from the indicated groups, with expression levels normalized to those of β-actin (*n* = 3). (**M**, **N**) ITT and GTT were performed in mice of the indicated groups and the AUC was calculated (*n* = 6–7). (**O**, **P**) Western blot analysis of insulin signaling pathway activation in the livers of mice from the indicated groups (**O**). Protein levels were normalized to β-actin (**P**) (*n* = 3). Samples were derived from the same experiment and blots were processed in parallel. (**Q**, **R**) Western blot analysis of protein levels of phosphoenolpyruvate carboxykinase in the livers of mice from the indicated groups (**Q**), and the protein levels were normalized to β-actin (**R**) (*n* = 3). Samples were derived from the same experiment and blots were processed in parallel. (**S**) Western blot analysis of protein levels of PDK4 in liver tissues from normal and MAFLD patients (*n* = 6). Samples were derived from the same experiment and blots were processed in parallel. Data information: Data are presented as mean ± SD. Biological replicates are shown in (**C**–**H**, **L**, **M**, **N**). For (**M**, **N**), significance was determined using two-way ANOVA. For (**C**–**H**, **K**–**L**, **P**, **R**) and the AUCs in (**M**, **N**), significance was determined using one-way ANOVA. ***P < 0.001, ****P < 0.0001 as indicated. TG triglyceride, CHOL cholesterol, IRS1 insulin receptor substrate 1, AUC area under the curve, PEPCK2 phosphoenolpyruvate carboxykinase 2. Source data are available online for this figure.

To confirm the lipid-regulatory role of PDK4 in *Vgll4*-overexpressing mice, we employed AAV2/8-mediated knockdown to suppress hepatic PDK4 expression (Fig. 6A). Suppression of *Pdk4* markedly decreased the liver-to-body weight ratio (Fig. 6B,C), hepatic triglyceride and total cholesterol contents (Fig. 6D,E), as well as serum cholesterol levels in VGLL4-OE mice (Fig. 6F). Furthermore, *Pdk4* knockdown alleviated hepatic injury, as evidenced by lower serum ALT and AST levels compared with the OE-NC group (Fig. 6G,H). Consistent with these findings, Oil Red O and H&E staining revealed fewer lipid droplets in hepatic tissues of mice with *Vgll4* overexpression plus *Pdk4* knockdown than those with *Vgll4* overexpression alone (Fig. 6I). In line with these findings, both protein and mRNA levels of lipogenic and lipid-uptake-related genes were markedly downregulated in *Pdk4*-deficient livers (Fig. 6J–L). With respect to glucose metabolism, *Pdk4* knockdown ameliorated insulin resistance and improved glucose intolerance (Fig. 6M,N), accompanied by restored phosphorylation of key insulin-signaling proteins and reduced rate-limiting enzymes involved in gluconeogenesis (Fig. 6O–R). Finally, in human MAFLD liver samples, Western blot analysis revealed an increase in PDK4 expression along with disease progression, consistent with the expression pattern of VGLL4 (Fig. 6S). Collectively, these findings establish PDK4 as a functionally important downstream effector that contributes to VGLL4-driven hepatic lipid accumulation and metabolic dysfunction.

### Super-TDU relieves hepatosteatosis induced by *Vgll4* overexpression and HFD feeding

**Figure EV5:**
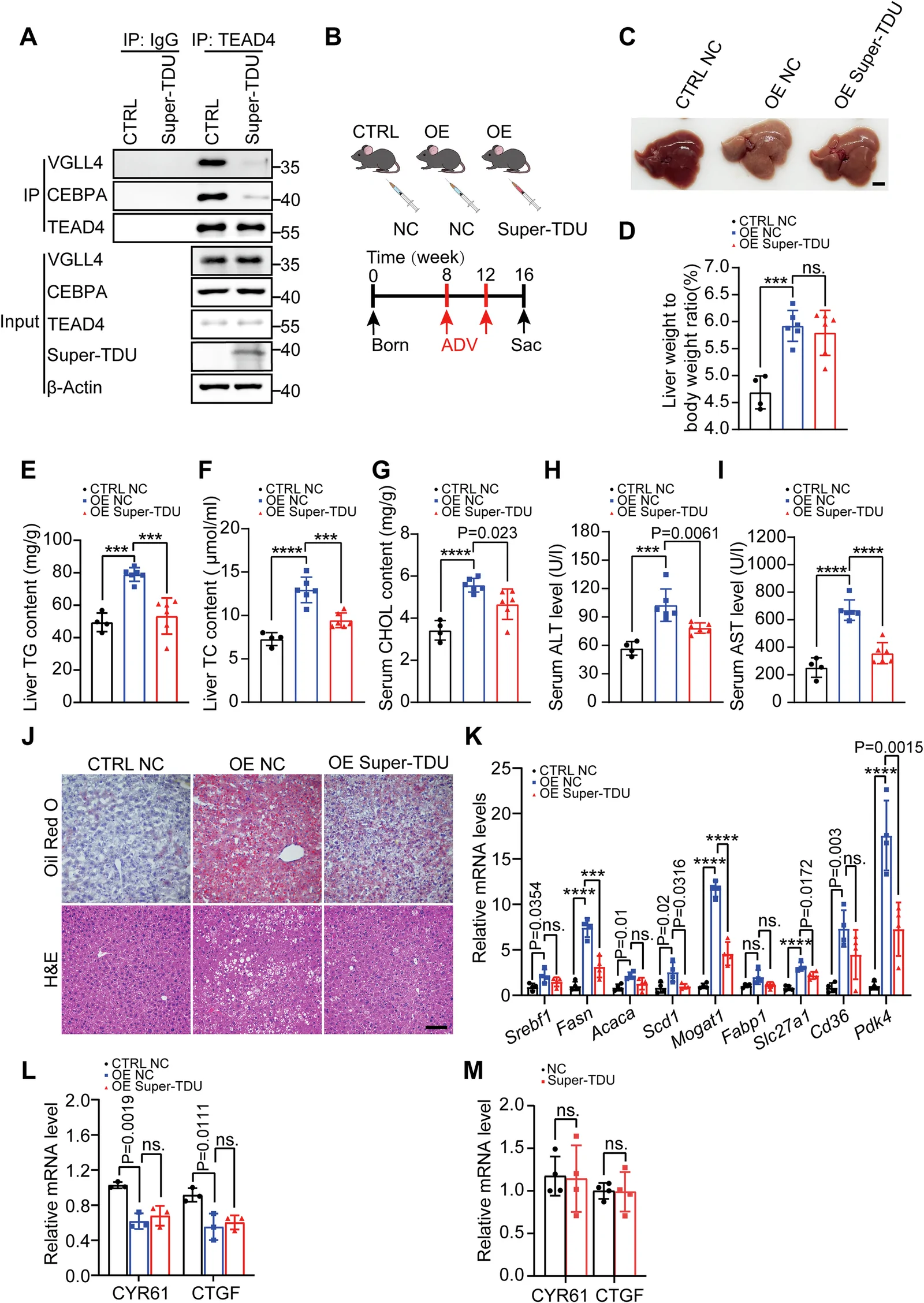
Administration of Super-TDU relieves hepatosteatosis induced by *Vgll4* overexpression. (**A**) Endogenous TEAD4 Co-IP assay in L02 cells. Cellular extracts were subjected to IP with an anti-TEAD4 antibody or IgG control. IP samples were probed for coprecipitating endogenous proteins by immunoblot. Samples were derived from the same experiment and blots were processed in parallel. (**B**) Schematic diagram of animal experiments. OE mice were injected with ADV-Super-TDU (OE Super-TDU) or the corresponding ADV negative control vector (OE NC). CTRL mice received the ADV negative-control vector (CTRL NC), and metabolic phenotypes were subsequently assessed. (**C**) Representative images of the livers (*n* = 3). Scale bar, 5 mm. (**D**–**F**) Liver-to-body weight ratio (**D**), liver TG (**E**) and TC (**F**) from the indicated groups (*n* = 4–6). (**G**–**I**) Serum CHOL (**G**), ALT (**H**) and AST (**I**) from the indicated groups (*n* = 4–6). (**J**) Representative images of Oil Red O-stained (upper panel) and H&E-stained (lower panel) liver sections (*n* = 3). Scale bar, 100 µm. (**K**–**M**) Relative mRNA levels of genes from the indicated groups, with expression levels normalized to those of β-actin (*n* = 3–4). Data information: Data are presented as mean ± SD. Biological replicates are shown in (**D**–**I**, **K**–**M**). For (**M**), significance was determined by Welch’s *t* test. For (**D**–**I**, **K**, **L**), significance was determined using one-way ANOVA. ***P < 0.001, ****P < 0.0001, ns. indicates no significant difference. TG triglyceride, CHOL cholesterol. Source data are available online for this figure

Super-TDU, a peptide mimicking VGLL4’s tandem Tondu domains, interacts with TEAD4 and has shown antitumor efficacy in cancer models (Jiao et al, 2014). Here, we employed Super-TDU to disrupt the VGLL4-TEAD4 interaction and assessed its impact on PDK4 expression and the progression of MAFLD. Co-IP assays at endogenous levels showed that Super-TDU treatment significantly reduced the association of VGLL4 and CEBPA with TEAD4 compared to controls (Fig. EV5A), confirming its inhibition of VGLL4/TEAD4/CEBPA formation by blocking the VGLL4–TEAD4 interaction and subsequent TEAD4–CEBPA enhancement.

We injected ADV-encoding Super-TDU peptides into *Vgll4*-overexpressing mice and included corresponding control groups to assess the phenotypic changes (Fig. EV5B). Macroscopic examination of the liver revealed that Super-TDU injection significantly reversed the pale liver phenotype caused by *Vgll4* overexpression (Fig. EV5C). However, Super-TDU did not reduce the liver-to-body weight ratio increase induced by *Vgll4* overexpression, likely due to the high level of VGLL4 expression, which may have prevented Super-TDU from fully inhibiting the VGLL4-TEAD4 interaction (Fig. EV5D). Despite this, Super-TDU administration reduced hepatic TG and TC levels, lowered serum CHOL, and significantly alleviated liver injury (Fig. EV5E–I). Histological analysis using Oil Red O and H&E staining revealed a marked reduction in hepatic lipid accumulation in Super-TDU-treated mice, along with a downregulation of mRNA levels of key genes involved in lipid synthesis and absorption (Fig. EV5J,K). Notably, *Pdk4* levels in the liver of Super-TDU-treated mice were significantly lower compared to those in *Vgll4*-overexpressing mice (Fig. EV5K). Together, these findings suggest that Super-TDU effectively blocks the VGLL4–TEAD4 interaction in *Vgll4*-overexpressing mice, leading to reduced PDK4 expression and ultimately alleviating the development of hepatosteatosis.

**Figure 7:**
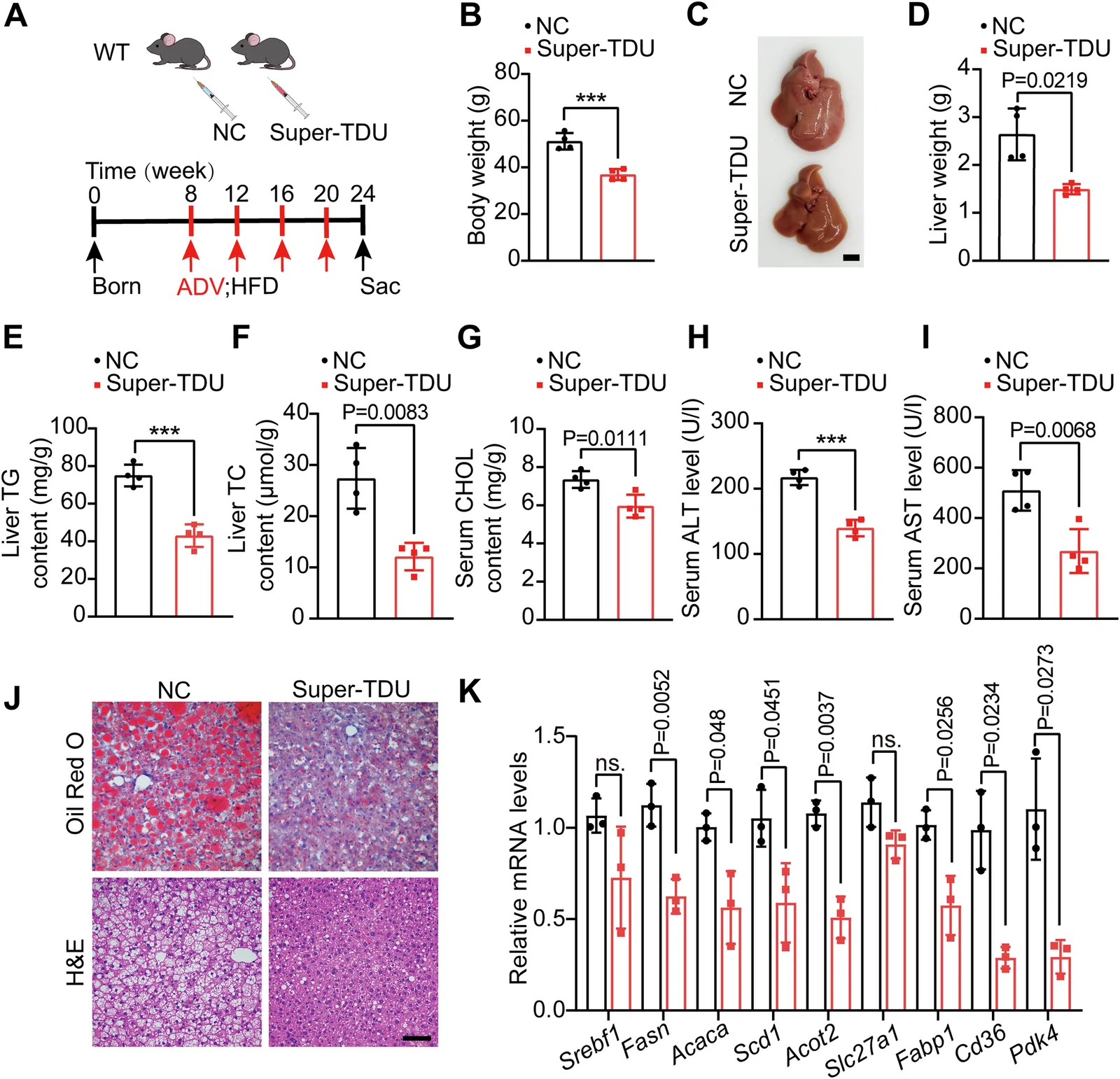
Administration of Super-TDU relieves hepatosteatosis induced by HFD. (**A**) Schematic diagram of animal experiments. Eight-week-old mice were injected via the tail vein with either ADV-Super-TDU (Super-TDU) or ADV negative control vector (NC) at 8, 12, 16, and 20 weeks of age. All mice were fed an HFD for 4 months starting at week 8. (**B**) Body weight from the indicated groups (*n* = 4). (**C**) Representative images of the livers (*n* = 3). Scale bar, 5 mm. (**D**–**F**) Liver weight (**D**), liver TG (**E**) and TC (**F**) from the indicated groups (*n* = 4). (**G**–**I**) Serum CHOL (**G**), ALT (**H**) and AST (**I**) from the indicated groups (*n* = 4). (**J**) Representative images of Oil Red O-stained (upper panel) and H&E-stained (lower panel) liver sections (*n* = 4). Scale bar, 100 µm. (**K**) Relative mRNA levels of genes involved in fatty acid synthesis and uptake from the indicated groups, with expression levels normalized to those of β-actin (*n* = 3). Data information: Data are presented as mean ± SD. Biological replicates are shown in (**B**, **D**–**I**, **K**). For (**B**, **D**–**I**, **K**), significance was determined by Welch’s *t* test. ***P < 0.001, ns. indicates no significant difference. TG triglyceride, CHOL cholesterol, HFD high-fat diet. Source data are available online for this figure.

After confirming that Super-TDU effectively alleviated hepatosteatosis in *Vgll4*-overexpressing mice, we further evaluated its therapeutic potential in HFD-fed mice, given the trend of VGLL4 upregulation observed under HFD conditions in our models (Fig. EV1E). Wild-type (WT) mice received monthly injections of ADV-Super-TDU and were initiated on HFD feeding at eight weeks of age (Fig. 7A). Super-TDU administration significantly reduced body weight (Fig. 7B), liver weight (Fig. 7C,D), hepatic triglyceride and total cholesterol levels (Fig. 7E,F), as well as serum cholesterol content, ALT and AST levels (Fig. 7G–I). Consistently, Oil Red O and H&E staining demonstrated that Super-TDU markedly reduced hepatic lipid accumulation in HFD-fed mice (Fig. 7J). The expression of genes involved in fatty acid synthesis and uptake was downregulated in Super-TDU-treated mice (Fig. 7K). Additionally, we evaluated the impacts on canonical Hippo signaling by examining the classic YAP/TAZ–TEAD target genes, *Cyr61* and *Ctgf*. In OE-NC livers, the expression of *Cyr61* and *Ctgf* was significantly decreased compared with CTRL NC controls, consistent with the role of elevated VGLL4 in antagonizing the YAP–TEAD4 interaction (Fig. EV5L). Notably, although ADV-Super-TDU treatment successfully alleviated hepatic steatosis, it failed to markedly alter or restore *Cyr61* or *Ctgf* expression under *Vgll4*-overexpressing or basal conditions (Fig. EV5L,M). Collectively, these data provide compelling evidence that Super-TDU effectively mitigates hepatosteatosis by uncoupling the VGLL4–TEAD4 transcriptional complex, thereby underscoring its therapeutic potential as a targeted metabolic intervention.

## Discussion

The present study was initially inspired by the observation that VGLL4 is highly enriched in hepatocytes of MAFLD patients, with its expression upregulated compared to normal individuals. This research elucidates VGLL4’s role in hepatic steatosis and MAFLD progression, demonstrating that VGLL4 promotes hepatosteatosis through TEAD-dependent transcriptional mechanisms. Hepatocyte-specific *Vgll4* overexpression drives hepatic lipid accumulation, insulin resistance, and inflammation, whereas *Vgll4* deficiency attenuates HFD-induced steatosis and associated metabolic disruptions. Mechanistically, VGLL4 enhances TEAD4–CEBPA cooperation at the *Pdk4* promoter, a pivotal effector linking glucose and lipid metabolism. Notably, YAP competes with VGLL4 for TEAD4 binding, thereby suppressing this pro-steatotic transcriptional pathway, reducing PDK4 expression, and limiting lipid accumulation. These findings suggest that the antagonistic relationship between YAP and VGLL4 also plays a role in hepatic metabolism. Furthermore, Super-TDU offers a promising therapeutic tool for alleviating MAFLD symptoms. These results define a new regulatory pathway in hepatocyte metabolism, with implications for targeted therapies in metabolic liver diseases.

Hepatocyte-specific *Vgll4* overexpression induced pronounced steatosis. Extending beyond lipids, VGLL4 impaired insulin signaling while elevating gluconeogenic enzymes, thereby diminishing glycogen stores and promoting glucose intolerance. This aligns with our hypothesis that VGLL4 disrupts hepatic glucose homeostasis, fueling a feed-forward loop where insulin resistance exacerbates steatosis. Building on this, VGLL4 appears to provoke a robust insulin resistance phenotype, yet exerts no discernible impact on insulin signaling in homeostatic conditions (Appendix Fig. S6H, I). This context-dependent effect implies that enhanced lipogenesis is unlikely to be a consequence of altered insulin signaling and that VGLL4 may not directly regulate the insulin pathway.

Although VGLL4 was upregulated in hepatocytes from patients with MAFLD (Fig. EV1B,C), the mechanisms underlying this increase remain unknown. Existing literature identifies several regulators of VGLL4, including the E3 ubiquitin ligase UBR5, which promotes its degradation by mediating its polyubiquitination at Lys61 (Wei et al, 2025), and microRNAs like miR-222 and miR-130a, which destabilize *VGLL4* mRNA to enhance YAP activity and tumor progression (Li et al, 2015; Shen et al, 2015). Additionally, ACSL4 has also been reported to reduce VGLL4 expression in microglia under inflammatory conditions (Zhou et al, 2023). Whether these mechanisms are conserved in hepatocytes has not been established. Thus, the elevation of VGLL4 observed in MAFLD may reflect hepatocyte-specific regulation at the transcriptional, post-transcriptional, or post-translational level. Defining these regulatory mechanisms will be important for understanding how this VGLL4-dependent transcriptional pathway becomes activated during hepatic steatosis.

Previous studies have reported divergent roles of YAP in metabolic liver disease. Hepatic YAP promotes lipogenesis and steatosis through its interaction with SREBPs in diabetic mice (Shu et al, 2019), whereas fatty acid-induced YAP activation has been shown to stimulate profibrogenic signaling in MAFLD (Salloum et al, 2021). More recently, hepatocyte-specific *Yap* deletion was found to reduce steatosis but exacerbate hepatic inflammation and fibrosis in dietary MASH models (Sun et al, 2026). In our study, YAP attenuated VGLL4-induced *Pdk4* expression and lipid accumulation by weakening VGLL4-dependent TEAD4–CEBPA association. Together, these findings suggest that the effects of YAP on hepatic lipid accumulation may be distinct from its roles in metabolism dysfunction.

While PDK4 has been implicated in promoting hepatic lipid accumulation and sustaining lipogenic transcriptional programs (Zhang et al, 2018a; Deng et al, 2022), the disease-relevant transcriptional mechanisms remain incompletely defined. Here, we identify a promoter-level mechanism in which VGLL4 facilitates the coordinated action of TEAD4 and CEBPA to promote *Pdk4* transcription under metabolic stress. Our data support PDK4 as an important functional effector of VGLL4-driven steatosis; however, VGLL4 likely regulates hepatic lipid metabolism through a broader transcriptional and metabolic network (Figs. 1M and 5A). Indeed, *Pdk4* knockdown substantially alleviated, but did not fully abolish, VGLL4-induced hepatic lipid accumulation, suggesting that additional VGLL4-regulated downstream pathways may also contribute to this phenotype (Fig. 6). Furthermore, although direct evidence linking PDK4 to canonical lipogenic enzymes remains limited, its potential to orchestrate metabolic homeostasis via kinase-dependent remodeling of mitochondria-associated ER membranes (MAMs) in alcohol-associated liver disease highlights a highly promising, non-canonical mechanism (Thoudam et al, 2023). Thus, while we established PDK4 as a critical target of VGLL4, its exact downstream action warrants further exploration.

Super-TDU-mediated disruption of the VGLL4–TEAD4 interaction alleviated hepatic steatosis in both HFD-fed and *Vgll4*-overexpressing mice. Super-TDU was originally described as a YAP–TEAD antagonist in cancer models (Jiao et al, 2014). Super-TDU treatment did not markedly increase the canonical YAP/TAZ target genes *Cyr61* and *Ctgf* in either HFD-fed or *Vgll4*-overexpressing mice (Fig. EV5L,M). Because Super-TDU itself occupies TEADs, displacement of VGLL4 does not necessarily increase the pool of TEAD4 available for YAP/TAZ recruitment. Thus, Super-TDU may suppress VGLL4-dependent *Pdk4* transcription without restoring canonical YAP/TAZ–TEAD output (Figs. EV5A,K–M and  7K). Given the limited pharmacological options for MAFLD, these findings nominate the VGLL4–TEAD4 interface as a potential therapeutic target.

The present study has several limitations. First, our reliance on mouse models restricts direct extrapolation to humans, though validation with patient samples partially mitigated this limitation. Second, while our study demonstrated VGLL4’s lipogenic role in the liver, it did not fully explore its effects on inflammation. Finally, VGLL4 protein levels are upregulated in the livers of a subset of MAFLD patients, but the internal regulatory mechanism remains largely unknown.

In conclusion, our data demonstrate that hepatic VGLL4 modulates lipid homeostasis by promoting the interaction between TEAD4 and CEBPA, which in turn promotes PDK4 expression. Furthermore, pharmacological disruption of VGLL4–TEAD4 binding using Super-TDU effectively alleviates hepatic steatosis, highlighting the therapeutic potential of this regulatory pathway.

## Methods

**Table:** Reagents and tools table

Reagent/resource	Reference or source		Identifier or catalog number
**Experimental models**			
HEK293T	ATCC		ACS-4500
AML12	NCACC		SCSP-550
L02	NCACC		N/S
C57BL/6J mice	Animal Core Facility, SIBCB		N/S
*Vgll4*^*fl/fl*^ mice	Laboratory of Zhang Lei		N/S
*LAP-tTA* mice	Laboratory of Lijian Hui		N/S
*TetO-Vgll4* mice	Laboratory of Zhang Lei		N/S
*Mx1*-cre mice	Laboratory of Lijian Hui		N/S
Escherichia coli TOP10	ToloBio		CC96105
**Recombinant DNA**			
pcDNA3.1-FLAG		Addgene	Cat #208051
pGL3-basic		Addgene	Cat #212936
**shRNA targeting sequence**			
*Luc*		–	TTCCTGGAACAATTGCTTTTA
*VGLL4*		Human	GTTCTGTGCTATGAAGGTGAA
*TEAD1/3/4*		Human	ATGATCAACTTCATCCACAAG
*TEAD2*		Human	GAAGAGACGTGTGTGCAGGAA
*PDK4*		Human	CTTTGTCTTCTGAGTCTATAG
*Pdk4*		Mouse	AGACGCTATCATCTACTTAAA
**Primers for real-Time PCR**			
Mouse *Vgll4*		F: ATGAACAACAATATCGGCGTTCT R: GGGCTCCATGCTGAATTTCC	
Mouse *Srebf1*		F: CACTTCTGGAGACATCGCAAAC R: ATGGTAGACAACAGCCGCATC	
Mouse *Fasn*		F: CTGCGGAAACTTCAGGAAATG R: GGTTCGGAATGCTATCCAGG	
Mouse *Acaca*		F: GGCCAGTGCTATGCTGAGAT R: AGGGTCAAGTGCTGCTCCA	
Mouse *Scd1*		F: GCTGAACACCCATCCCGAG R: TGTAAGAACTGGAGATCTCTTGGA	
Mouse *Acot2*		F: CAGCCACCCCGAGGTAAAAG R: AGGGACACAGGGGGTATAGT	
Mouse *Cd36*		F: TGGGTTTTGCACATCAAAGA R: GATGGACCTGCAAATGTCAGA	
Mouse *Pdk4*		F: CAGGTTATGGGACAGACGCT R: CCCTGCTTGGGATACACCAG	
Mouse *Acot2*		F: CAGCCACCCCGAGGTAAAAG R: AGGGACACAGGGGGTATAGT	
Mouse *Mogat1*		F: CAAAGGCTCCTGGCTACGAA R: GGCAAGGCTACTCCCATTGA	
Mouse *Fabp1*		F: AGGAGTGCGAACTGGAGACCAT R: GTCTCCATTGAGTTCAGTCACGG	
Mouse *Slc27a1*		F: CTGGGACTTCCGTGGACCT R: TCTTGCAGACGATACGCAGAA	
Mouse *Ppara*		F: AGAACCTGAGGAAGCCGTTC R: TTAAGCACGTGCACAATCCC	
Mouse *Cpt1a*		F: TGACGGCTATGGTGTTTCCT R: TTTTGGAATTGGCGGTGAGG	
Mouse *Acox1*		F: GGCCGCTATGATGGCAATGT R: GGGCTTCAAGTGCTTGTAGTAAG	
Mouse *Acadm*		F: CTGACGCCGTGCAGATTTTC R: CACGAGCTATGATCAGCCTCT	
Mouse *Il1β*		F: GCAAGGGCTTCAGGCAGGCCGCG R: GGTCATTCTCCTGGAAGGTCTGTGGGC	
Mouse *Ccl2*		F: CACTCACCTGCTGCTACTCA R: GCTTGGTGACAAAAACTACAGC	
Mouse *Ccl5*		F: GCTGCTTTGCCTACCTCTCC R: TCGAGTGACAAACACGACTGC	
Mouse *Mmp13*		F: GACAAGCAGTTCCAAAGGCTAC R: ATGGGAAACATCAGGGCTCC	
Mouse *Col1a1*		F: GCTCCTCTTAGGGGCCACT R: CCACGTCTCACCATTGGGG	
Mouse *Col1a2*		F: GTAACTTCGTGCCTAGCAACA R: CCTTTGTCAGAATACTGAGCAGC	
Mouse *Col3a1*		F: CTGTAACATGGAAACTGGGGAAA R: CCATAGCTGAACTGAAAACCACC	
Mouse *Col4a1*		F: CTGGCACAAAAGGGACGAG R: ACGTGGCCGAGAATTTCACC	
Mouse *Col4a2*		F: TGTGGGCATGAAAGGTCTGT R: CGACCCTCGATCACCTTTCT	
Mouse *Col5a2*		F: GTACCACTGGGCAAAGAGGA R: CTTTTCCTGGTGTACCCGCT	
Mouse *Col6a3*		F: GCCCAACAGCATGGAGATGT R: CTTCCCAGCACTCCAAGAGG	
Mouse *Pdch7*		F: AGTACAGCAAACAGCCATTTCG R: ACTCTGCAGTGACCCCTGAT	
Mouse *Actb*		F: GTGACGTTGACATCCGTAAAGA R: GCCGGACTCATCGTACTCC	
Human *TEAD1*		F: TCCACCAAAGTTTGCTCCTT R: GCCATTCTCAAACCTTGCAT	
Human *TEAD2*		F: TTTTGGTCTGGAGGATCTGG R: ATGGGGGAGTCAGTGACAAG	
Human *TEAD3*		F: CATCCACAAGCTGAAGCAC R: AGCAATGACAAGCAGGGTCT	
Human *TEAD4*		F: GAACGGGGACCCTCCAATG R: GCGAGCATACTCTGTCTCAAC	
Human *FASN*		F: TCTACTACAAGCTGCGTGCC R: CGGATACTTTCCCGTCGCAT	
Human *ACACA*		F: GCGGAGTGGCTAGAGAAACA R: GGGTGATATGTGCTGCGTCA	
Human *PPARG*		F: GTGACGTTGACATCCGTAAAGA R: GCCGGACTCATCGTACTCC	
Human *SCD1*		F: TGCGATATGCTGTGGTGCTT R: GTTGATGTGCCAGCGGTACT	
Human *IL1β*		F: GCAAGGGCTTCAGGCAGGCCGCG R: GGTCATTCTCCTGGAAGGTCTGTGGGC	
Human *VGLL4*		F: AATCAGCCCCAGCAAGAGGAAG R: GCAGTCTCCATTGGCAGTCTTG	
Human *ACTB*		F: CATGTACGTTGCTATCCAGGC R: CTCCTTAATGTCACGCACGAT	
**Antibodies**			
VGLL4	Abclonal		Cat# A18248, RRID:AB_2862024
p-IRS1^Ser307^	Cell Signaling Technology		Cat# 2381
IRS1	Cell Signaling Technology		Cat# 2382
p-AKT^Ser473^	Cell Signaling Technology		Cat# 9271, RRID:AB_329825
AKT	Cell Signaling Technology		Cat# 9272, RRID:AB_329827
p-FOXO1^Ser256^	Cell Signaling Technology		Cat# 84192, RRID:AB_2800035
FOXO1	HUABIO		Cat# ET1608-25, RRID:AB_3069795
PEPCK2	Proteintech		Cat# 14892-1-AP, RRID:AB_2160044
G6Pase	Proteintech		Cat# 66860-1-LG
TEAD4	Abcam		Cat# ab58310, RRID:AB_945789
CEBPA	Abcam		Cat# Ab317442
FAS	Abcam		Cat# ab22759, RRID:AB_732316
ACCα	Cell Signaling Technology		Cat# 4190, RRID:AB_10547752
SCD1	Cell Signaling Technology		Cat# 2438, RRID:AB_823634
PDK4	Abclonal		Cat# A13337, RRID:AB_2760193
CYR61	Cell Signaling Technology		Cat# 14479, RRID:AB_2798492
TAZ	Cell Signaling Technology		Cat# 83669, RRID:AB_2800026
YAP1	Sigma-Aldrich		Cat# WH0010413M1, RRID:AB_1844253
β-Actin	Abclonal		Cat# AC026, RRID:AB_2768234
IgG	Proteintech		Cat# B900620, RRID:AB_2883054
IgG	Proteintech		Cat# 30000-0-AP, RRID:AB_2819035
Flag	Sigma-Aldrich		Cat# F3165, RRID:AB_259529
Flag	Sigma-Aldrich		Cat# F7425, RRID:AB_439687
HA	GNI		Cat# GNI4110-MC, RRID:AB_3076243
HA	Invitrogen		Cat# 71-5500, RRID:AB_87935
Myc	Tiandi Renhe		Cat# SLAB2900, RRID:AB_3662587
Myc	Sigma-Aldrich		Cat# C3956, RRID:AB_439680
**Chemicals, enzymes and other reagents**			
Fetal bovine serum	ExCell Bio		Cat# 021C0420A
Protease Inhibitor Cocktail	MedChemExpress		Cat# HY-K0010
QIAQIUCK PCR Purification Kit	QIAGEN		Cat# 28104
37% Formaldehyde	Sigma		Cat# 50-00-0
2 × Phanta Max Master Mix	Vazyme		Cat# P515-03
ClonExpress II One Step Cloning Kit	Vazyme		Cat# C112-01
AceQ Universal SYBR qPCR Master Mix	Vazyme		Cat# Q511-02
RNA Isolater Total RNA Extraction Reagent	Vazyme		Cat# R401-01
HiScript III RT SuperMix for qPCR ( + gDNA wiper)	Vazyme		Cat# R323-01
Dual-Luciferase reporter assay system	Promega		Cat# E1960
**Software**			
ImageJ 1.52a	https://imagej.net/		N/S
GraphPad Prism 10	GraphPad		N/S
Adobe Illustrator	Adobe		N/S
Snapgene	https://www.snapgene.com/		N/S
Leica Application Suite	https://www.leica-microsystems.com/		N/S
**Other**			
RNA-seq RAW data	China National Center for Bioinformation		CRA032116
CUT&Tag RAW data	China National Center for Bioinformation		CRA042790

### Human liver samples

All procedures involving human samples conformed to the ethical guidelines of the 1975 Declaration of Helsinki and were approved by the Institutional Review Board and the Ethics Committee for Human Research of Zhongshan Hospital, Fudan University (B2025-687). Written informed consent was obtained from each patient (or their legally authorized representatives) prior to tissue collection, covering both tissue acquisition and the collection of clinical information. Human liver samples, including those from patients with MAFLD, were obtained from patients who underwent liver resection for hepatic cancer or cyst in the Department of Liver Surgery, Zhongshan Hospital. Non-steatotic control and steatotic regions were collected from the resected liver tissues. MAFLD was diagnosed by pathologists in a blinded manner. Samples with liver steatosis caused by excessive alcohol consumption, drug/toxin exposure, or hepatitis virus infection were excluded to ensure the samples were specific to MAFLD. The clinical characteristics of each patient are provided in Appendix Table S1.

### Experimental animals

All animal experimental procedures complied with the National Institutes of Health (NIH) *Guide for the Care and Use of Laboratory Animals*. All mice were housed in a specific pathogen-free (SPF) environment. Some were kept at the Center for Excellence in Molecular Cell Science, with protocols approved by the Institutional Animal Care and Use Committee (Approval No. SIBCB-S328-1511-052-C01), and others were housed at Shanghai Jiao Tong University, with protocols approved by the Laboratory Animal Center (Approval No. A2023115-003).

*LAP-tTA*, *TetO-Vgll4*, *Mx1*^*cre*^ and *Vgll4*^*f/f*^ mice were used in this study. The generation of the transgenic *TetO-Vgll4* mouse line has been previously described (Wang et al, 2022). Briefly, this line was generated by inserting a tetO-Vgll4-Flag-wpre-polyA cassette behind the 3′UTR of the *Col1a1* gene. To generate hepatocyte-enriched *Vgll4*-overexpressing mice, *TetO-Vgll4* mice were crossed with LAP-tTA mice (kindly provided by Prof. Lijian Hui), in which tTA expression is driven by the liver-enriched LAP promoter. This model functions as a Tet-off system: in the absence of doxycycline, tTA binds to the tetO-responsive element and activates *Vgll4*-Flag expression. Doxycycline was not administered in the experiments using *LAP-tTA; TetO-Vgll4* mice; therefore, *Vgll4*-Flag was constitutively overexpressed from early life in *LAP-tTA*-positive mice. The sibling littermates without the *LAP-tTA* allele were used as non-*Vgll4* overexpressing controls and maintained under the same doxycycline-free conditions.

The hepatic *Vgll4*-knockout (*Vgll4*-HKO) mice were generated as follows. *Vgll4*^*f/f*^ mice were generated as we previously described (Yu et al, 2019). In this mouse line, exon 2 of the *Vgll4* gene was flanked by two *loxP* sites using CRISPR/Cas9. Upon crossbreeding with *Mx1*^*cre*^ mice which were also kindly provided by Prof. Lijian Hui, successful *Vgll4* knockout and loss of VGLL4 protein expression were verified by Western blot (Fig. 3J). Poly I:C (2 mg/mL, GE27-4732-01, Sigma-Aldrich) was prepared in PBS. A dose of 13 mg/kg body weight was intraperitoneally injected into 8-week-old male mice twice at 24-h intervals. Mice were analyzed 4 months post-poly I:C injection.

All mice, which were on a C57BL/6 J background and had undergone eight generations of backcrossing following standard procedures, were housed in individually ventilated cage (IVC) standard, with a 12-h light/dark cycle at room temperature. Mice in the high-fat diet (HFD) group were fed a typical 60% HFD (D12492, 60 kcal% fat, 20 kcal% carbohydrates, and 20 kcal% protein, Research Diets), while those in the control diet group received a standard chow diet (P1300F-25, 10 kcal% fat, 70 kcal% carbohydrates, and 20 kcal% protein; Shanghai Puluteng Co. Ltd.).

### Public RNA-sequencing data

RNA-seq data were obtained from the Gene Expression Omnibus (GEO) database: transcriptomic profiling across the spectrum of MAFLD (GSE135251). These data comprise 216 snap-frozen liver biopsies, of which 206 are from MAFLD/MASH cases.

### ITT and GTT experiment

Mice were intraperitoneally injected with glucose at a dosage of 1.5 g/kg body weight after an overnight fast for the glucose tolerance test. Insulin tolerance tests were performed after a 4-h fast, followed by intraperitoneal injection of insulin at 1.5 IU/kg or 0.5 IU/kg. Blood glucose levels were measured at 0, 15, 30, 60, 90, and 120 min using blood glucose test strips (Roche, 06454038).

### Liver triglyceride and total cholesterol assay

Liver triglyceride and total cholesterol assays were performed using triglyceride content assay kit and total cholesterol content assay kit (BC1985 and BC0625, Solarbio).

### Administration of adeno-associated virus and adenovirus

For adeno-associated virus serotype 2/8 (AAV2/8)-mediated gene delivery, eight-week-old mice received an intravenous injection of 5 × 10^11^ viral particles through the tail vein. For hepatic *Pdk4* knockdown, mice were injected with AAV-sh*Pdk4* or the corresponding control virus. The shRNA sequence targeting *Pdk4* was 5′-AGACGCTATCATCTACTTAAA-3′. For hepatocyte-targeted *Vgll4* deletion, *Vgll4*^*f/f*^ mice were injected with AAV2/8-TBG-Cre or the corresponding AAV2/8-TBG control virus. AAV2/8 viruses were provided by the Gene Editing Facility of CEBSIT. Adenovirus (ADV) packaging and purification were performed as previously described (He et al, 1998). For adenovirus-mediated gene delivery, mice were intravenously injected with 5 × 10^8^ plaque-forming units (PFU) of the indicated ADV vectors through the tail vein. Glucose tolerance tests (GTT) and insulin tolerance tests (ITT) were performed at the indicated time points. Tissues were collected for gene expression and histological analyses, and serum samples were used to measure triglycerides (TG), alanine aminotransferase (ALT), and aspartate aminotransferase (AST).

### Histology and immunohistochemistry staining

Mouse liver samples were fixed in 4% (w/v) paraformaldehyde (PFA, P6148, Sigma-Aldrich) overnight, followed by equilibration in 30% sucrose at 4 °C for 2–3 days. Subsequently, the dehydrated liver tissues were embedded in Tissue-Tek optimal cutting temperature (OCT, H-LD-803, Thermo) compound, and 10-μm thick frozen sections were prepared. These sections were stained with Oil Red O (O1391, Sigma-Aldrich) to assess hepatic lipid droplet accumulation. For immunofluorescence staining, sections were first washed with phosphate-buffered saline (PBS, BL1425A, Biosharp) and then blocked in PBS supplemented with 5% bovine serum albumin (BSA, A8020, Solarbio) and 1% Triton X-100 (V900502, Sigma-Aldrich) at room temperature. Next, sections were incubated with primary antibodies at 4 °C overnight, followed by three washes with PBS containing 0.1% Triton X-100. Sections were then incubated with Alexa Fluor-conjugated secondary antibodies and 4’,6-diamidino-2-phenylindole (DAPI, C1002, Beyotime) for 1 h at room temperature, and images were acquired using a Leica STELLARIS 5 confocal microscope.

For paraffin sections, liver samples from the same cohort were fixed in 4% paraformaldehyde for 24 h, followed by gradient dehydration through ethanol solutions, clearing in xylene, and embedding in paraffin. In all, 5-μm-thick paraffin sections were then prepared, dewaxed, and rehydrated prior to staining: they were typically stained with hematoxylin and eosin (H&E) for general histological assessment, and some were subjected to periodic acid-Schiff (PAS, C0142, Beyotime) staining for glycogen content assessment. The slides were observed using an Olympus BX63 microscope.

### Cell culture and stimulation

Mouse hepatocyte AML12, human embryonic kidney HEK293T cells and human fetal hepatocyte L02 cells were maintained in Dulbecco’s Modified Eagle Medium (DMEM, C11995500BT, Gibco) and RPMI 1640 medium (1640, C11875500BT, Gibco), respectively. Both media were supplemented with 10% fetal bovine serum (FBS, A5669701, Gibco) and 1% penicillin–streptomycin solution (15140163, Gibco), and the cells were cultured at 37 °C in a humidified incubator with 5% CO_2_. All cell lines were tested for mycoplasma contamination.

### PA-induced hepatosteatosis model and insulin stimulation in L02 Cells

100 mM palmitic acid (PA, P0500, Sigma-Aldrich) was dissolved in 0.1 M NaOH at 95 °C for 30 min, and 100 mM oleic acid (OA, O1383, Sigma-Aldrich) was dissolved in 0.1 M NaOH at 95 °C for 10 min; the two solutions were separately added to 10% (weight/volume) bovine serum albumin (BSA, A8850, Solarbio) while still hot, to prepare 10 mM stock solutions respectively. The stock solutions were sterilized through a 0.22-μm filter (FPE204030, Jet BIOFIL). L02 cells maintained under normal conditions were starved overnight in 1640 medium containing 1% FBS before being stimulated with 0.9 mM PA/OA (molar ratio OA:PA = 2:1). After PA/OA stimulation, cells were washed with PBS, fixed in 4% PFA, and then washed with PBS followed by 60% isopropanol. Neutral lipid content in L02 cells was visualized by 0.3% (w/v) ORO staining followed by washing in PBS, and counterstaining with hematoxylin. To assess fatty acid uptake, L02 cells (after 20 h PA/OA stimulation) were incubated with a fluorescent saturated fatty acid, BODIPY^TM^ 558/568 C_12_ (D3835, Thermo Fisher Scientific) for 4 h at room temperature. This probe was first prepared as a 10 mM stock solution in dimethyl sulfoxide (DMSO), then diluted to a final concentration of 6 nM in the cell culture medium before incubation. After incubation, cells were harvested, washed with PBS and fixed in 4% PFA for 10 min. For insulin stimulation assays, the L02 cells were synchronized in serum-free medium overnight, then stimulated with insulin (500 ng/mL, P3376, Beyotime) in serum-free medium.

### Immunoprecipitation and immunoblotting

For the immunoprecipitation assay, HEK293T cells were transfected with different plasmids using polyethylenimine (PEI). After 48 h, cells were harvested and lysed in IP buffer (50 mM Tris-HCl pH 8.0, 0.1 M NaCl, 1% NP40, 10% glycerol, 1.5 mM EDTA) supplemented with protease inhibitor cocktail (C0001, TargetMOI). After centrifugation (12,000×*g* for 10 min at 4 °C), cell lysates were divided into input groups and pulldown groups. For the pulldown groups, 20 µL of the indicated antigen-conjugated magnetic beads (B26101, B26201, B26301, Selleck) were added and incubated at room temperature for 1 h, followed by three washes with cold IP buffer. Samples were prepared in SDS loading buffer (2% SDS, 10% Glycerol, 50 mM Tris-HCl pH 6.8, 0.025% Bromophenol Blue, 5% β-Mercaptoethanol), subjected to SDS-polyacrylamide gel electrophoresis (SDS-PAGE), followed by immunoblotting with the indicated antibodies. All immunoblotting results were visualized using a Tanon-4200 Multi Imaging System.

### RNA extraction, reverse transcription, and real-time PCR

Total RNA was extracted from tissues and cells using TRIzol reagent (R401-01, Vazyme) and reverse-transcribed into cDNA using HiScript III RT SuperMix for qPCR (r323-01, Vazyme). The relative mRNA levels of the indicated genes were determined by quantitative real-time PCR (qPCR) using LightCycler™ 480 Instrument II (Roche) with SYBR green Master Mix (Q511-03, Vazyme). Results were normalized to β-actin expression (mouse Actb or human ACTB). Primers used in this study are listed in the Reagents & Tools table.

### Luciferase reporter assay

The promoter region of *Pdk4* (NC_000072.7: 5496128-5497290) was cloned into the pGL3-basic vector using NheI (FD0974, Thermo Fisher Scientific) and XhoI (FD0694, Thermo Fisher Scientific) restriction enzymes. L02 cells were seeded in 24-well plates and co-transfected with the indicated protein-coding plasmids and firefly luciferase reporter plasmids driven by *Pdk4* gene fragments. Renilla luciferase-expressing plasmids were co-transfected as internal controls for normalization. The total amount of plasmids was kept constant by adding equal amounts of the corresponding empty vectors. Cells were transfected with plasmids using ExFect Transfection Reagent (T101, Vazyme) for 12 h, then maintained under normal conditions for an additional 36 h. Cells were washed three times and harvested in passive lysis buffer (E1941, Promega). Finally, supernatants were assayed for both firefly and Renilla luciferase activities using the Dual-Luciferase™ Reporter Assay System (E1960, Promega) according to the manufacturer’s instructions. Luminescence was measured using a GloMax™ 20/20 Luminometer (Promega). Firefly luciferase activity was normalized to Renilla luciferase activity. Results are presented as the ratio of firefly to Renilla luciferase activity and are representative of at least three independent experiments.

### CUT&Tag assay

To evaluate the alterations in the genomic binding landscape of TEAD4, CUT&Tag assays were performed in WT and *Vgll4* knockdown (*Vgll4* KD) AML12 cells. The assay was executed using the Hyperactive Universal CUT&Tag Assay Kit for Illumina Pro (TD904, Vazyme) according to the manufacturer’s instructions. Briefly, cells were harvested, bound to Con A-coated magnetic beads, and incubated overnight at 4 °C with a specific primary antibody against TEAD4 or a normal IgG control. Following secondary antibody incubation, cells were treated with the hyperactive Tn5 transposome. To enable accurate quantitative normalization between the WT and *Vgll4* KD groups, *E. coli* spike-in DNA (provided in the kit) was introduced into each reaction. Tagmented DNA was extracted, PCR-amplified, and purified using VAHTS DNA Clean Beads (N411, Vazyme) to construct the sequencing libraries. The libraries were subjected to paired-end sequencing on an Illumina platform by Novogene (Beijing, China). Bioinformatic processing of the raw sequencing reads was performed using the standardized pipeline implemented on the Vazyme-Biocloud platform (http://cloud.vazyme.com:83/). Briefly, raw reads were quality-filtered and adapter-trimmed using cutadapt (v1.12). Clean reads were aligned to the mouse reference genome (mm10) using Bowtie2 (v2.2.9) with parameters --very-sensitive-local --no-unal --no-mixed --no-discordant -I 10 -X 700. Simultaneously, to calibrate signals across different samples, reads were mapped to the *E. coli* genome using identical parameters to calculate the spike-in scale factor for each sample. Alignment files were processed via SAMtools (v1.3), and potential PCR duplicates were marked using Picard MarkDuplicates (v1.119). To evaluate the local binding signals of TEAD4 at specific target loci, spike-in normalized read density tracks (BigWig files) were generated using the bamCoverage tool from deepTools (v3.5.1) and visualized via the Integrative Genomics Viewer (IGV).

### Chromatin immunoprecipitation-PCR (ChIP-PCR) and two-step ChIP assay

The ChIP and two-step ChIP assays were performed as previously described (He et al, 2021). Briefly, AML12 cells were seeded in 10-cm plates and transfected with the indicated protein-coding plasmids using Lipofectamine™ 3000 (L3000015, Thermo) for 48 h. After two washes with PBS, cells were fixed with 1% formaldehyde for 15 min at 37 °C to crosslink protein-DNA complexes followed by quenching with 0.125 M glycine at 37 °C for 5 min. Cells were scraped and lysed in ice-cold cell lysis buffer (50 mM HEPES-KOH pH 7.5, 140 mM NaCl, 1 mM EDTA pH 8.0, 1% Triton X-100, 0.1% sodium deoxycholate, 0.1% SDS) supplemented with protease inhibitor cocktail (4693132001, Roche). Genomic DNA was sheared into 200–500 bp fragments by sonication using an Ultrasonic Cell Grinder (JYD-900L, Shanghai Zhisun Equipment) on ice: 25 cycles (10 s sonication per cycle, interrupted by 20 s rest, 25% power) at 4 °C. Sonicated samples were centrifuged at 12,000×*g* for 10 min at 4 °C to collect supernatants. Antibodies against the target proteins or normal IgG (as a negative control) were used for immunoprecipitation overnight. Dynabeads™ Protein A beads (10002D, Thermo Fisher Scientific) were added to enrich antibody-protein-DNA complexes, followed by incubation at 4 °C for 2 h. Beads were then washed three times with wash buffer (150 mM NaCl, 2 mM EDTA pH 8.0, 1% Triton X-100, 0.1% SDS, 20 mM Tris-HCl) and once with high-salt wash buffer (500 mM NaCl, 2 mM EDTA pH 8.0, 1% Triton X-100, 0.1% SDS, 20 mM Tris-HCl). For two-step ChIP, AML12 cells expressing FLAG-CEBPA and MYC-TEAD4 were used. FLAG-CEBPA complexes were eluted from beads with 0.5 mg/mL 3×FLAG peptide (A6001, APExBIO) at 4 °C for 1 h; this elution step was repeated twice. The combined eluate was subjected to a second immunoprecipitation (re-ChIP) by incubating overnight at 4 °C with normal IgG (B900620, Proteintech) or anti-MYC (SLAB2903, Changzhou Tiandirenhe Co. Ltd.) antibody. After re-ChIP, the aforementioned washing procedure was repeated. Subsequently, the antibody-chromatin complexes were eluted from the Protein A beads in elution buffer (1% SDS, 100 mM NaHCO_3_) and subjected to reverse crosslinking by incubating with 0.28 M NaCl at 65 °C overnight. Proteinase K (20 mg/mL, 539480, MERCK) and 0.5 mM EDTA were added and incubated at 55 °C for 2 h to remove proteins. The remaining DNA fragments were purified by QIAquick PCR Purification Kit (28106, QIAGEN) and analyzed by real-time PCR to determine the abundance of target DNA regions. The qPCR primers used were as follows: Pdk4-ChIP-qPCR-F (5′-GTCCTTGCTTTCGAGGTCCA-3′), Pdk4-ChIP-qPCR-R (5′-AGCCATCTCAATGTCACGCA-3′).

### RNA sequencing and bioinformatics analysis of the hepatocytes

Liver hepatocytes were isolated as previously described (Jung et al, 2020). Mice were euthanized using CO2, and a catheter was inserted into the inferior vena cava to initiate perfusion until the liver turned pale and enlarged. The liver was then digested, and the resulting cell suspension was passed through a 70-μm strainer. The single-cell suspension was washed, centrifuged in 25% Percoll (P1644, Sigma-Aldrich), and the pellet was collected. Total RNA was extracted from liver hepatocytes by Trizol reagent, qualified using Agilent 5300 RNA Nano Kit. Only high-quality RNA samples (OD260/280 = 1.8–2.2, OD260/230 ≥ 2.0, RQN ≥ 6.5, 28S:18S ≥ 1.0, >1 μg) were used to construct sequencing libraries. RNA sequencing was performed at Shanghai Majorbio Biopharm Biotechnology Co., Ltd. according to the manufacturer’s instructions, and the data were analyzed using the free online Majorbio Cloud platform (cloud.majorbio.com). The transcriptome library was prepared following Illumina Standard mRNA Prep protocol, and the sequencing was performed on the Illumina NovaSeq X Plus platform (paired-end 150 bp, PE150) using NovaSeq Reagent Kit. Clean reads were aligned to the reference genome (Ensembl_GRCm38.p6) with HISAT2 (Version 2.1.0) software. RSEM (Version 1.3.1) software was used to quantify the expression level. Differentially expressed genes (DEGs) were identified by FDR < 0.05 and |log2FC | ≥1 with DESeq2 (Version 1.24.0). In addition, functional-enrichment analysis and Reactome enrichment analysis were performed to identify which DEGs were significantly enriched in metabolic pathways using a Bonferroni-corrected *P* value ≤ 0.05 compared with the whole-transcriptome background. Reactome enrichment analysis was carried out by Reactome (https://reactome.org/). Transcription factor enrichment analysis was performed using MSigDB (https://www.gsea-msigdb.org/gsea/msigdb), utilizing its C3:TF target gene sets. Analyses in R Studio with clusterProfiler employed hypergeometric tests, with significance set at FDR < 0.05.

### Graphics

The summary figure was created with BioRender.com. Some images were processed using Imaris software (Version 10.2.0; Oxford Instruments, Abingdon, UK).

### Statistical analysis

All data are presented as mean ± SD and were analyzed using GraphPad Prism software (version 8.2.1; GraphPad Software Inc., La Jolla, CA). Unless otherwise indicated, n denotes biologically independent samples or experiments; technical replicates are explicitly identified in the corresponding figure legends. For comparisons between two groups, Welch’s *t* test was used. For comparisons among multiple groups or across time-course measurements, one-way or two-way ANOVA was applied as appropriate. Data were considered statistically significant when the corresponding statistical test yielded a *P* value < 0.05. In the figures, asterisks denote statistical significance (***P < 0.001, ****P < 0.0001).

## Supplementary information

Appendix

Peer Review File

Dataset EV1

Source data Fig. 1

Source data Fig. 2

Source data Fig. 3

Source data Fig. 4

Source data Fig. 5

Source data Fig. 6

Source data Fig. 7

Figure EV1 Source Data

Figure EV2 Source Data

Figure EV3 Source Data

Figure EV4 Source Data

Figure EV5 Source Data

Appendix Figure Source Data

Expanded View Figures

## Data Availability

The RNA-sequencing and CUT&Tag datasets generated in this study have been deposited in the Genome Sequence Archive (GSA) under accession numbers CRA032116 and CRA042790, respectively. The source data of this paper are collected in the following database record: biostudies:S-SCDT-10_1038-S44319-026-00934-x.
