# Supplementary material for: VGLL4 enhances PDK4 transcription and promotes metabolic dysfunction-associated fatty liver disease
Source: EMBO Rep. 2026 Sep 22;27(20):5807–36. doi: 10.1038/s44319-026-00934-x (PMC13694279; doi:10.1038/s44319-026-00934-x)
Supplement: Supplementary file 1 [file 44319_2026_934_MOESM17_ESM.pdf]

Expanded View Figures

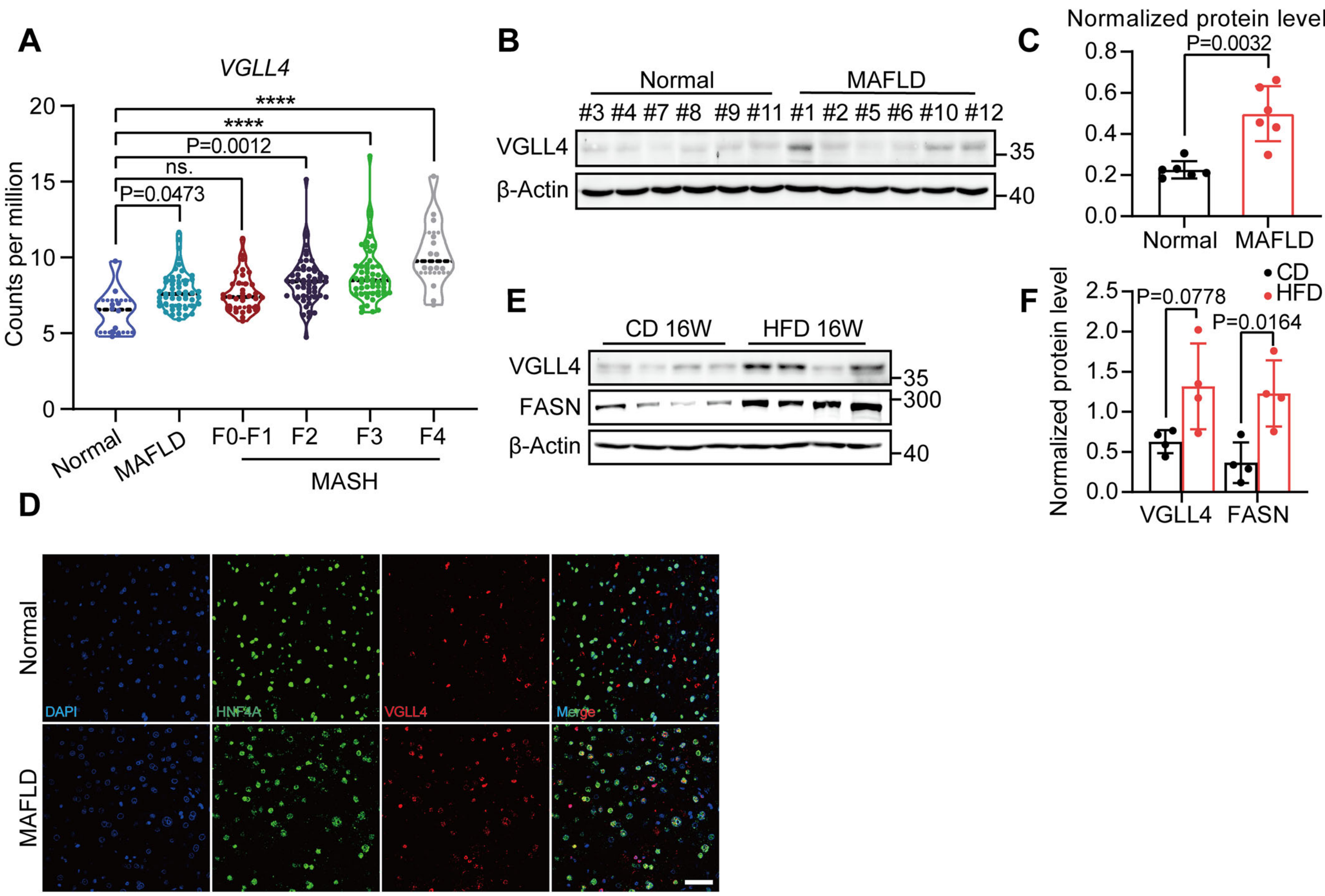

Figure EV1. VGLL4 protein expression is elevated in a subset of MAFLD liver samples.

(**A**) Violin plot depicting *VGLL4* expression in normal liver tissues, MAFLD liver tissues, and MASH liver tissues stratified by fibrosis stage based on the GSE135251 dataset ($n = 10$–54). (**B**) Western blot analysis of VGLL4 protein levels in liver tissues from controls and patients with MAFLD ($n = 6$). Samples were derived from the same experiment and blots were processed in parallel. (**C**) Quantification of VGLL4 protein levels normalized to β-actin ($n = 6$). (**D**) Immunofluorescence staining for VGLL4 and HNF4A in liver tissues from the indicated groups (scale bar, 20 μm). (**E**) Western blot analysis of protein levels of VGLL4 and FASN in liver tissues from CD and HFD mice ($n = 4$). Samples were derived from the same experiment and blots were processed in parallel. (**F**) Quantification of VGLL4 and FASN protein levels normalized to β-actin ($n = 4$). Data information: Data are presented as mean ± SD. Biological replicates are shown in (**A**). For (**C**, **F**), significance was determined by Welch's *t* test. For (**A**), significance was determined using one-way ANOVA. ****$P < 0.0001$, ns. indicates no significant difference. MAFLD metabolic dysfunction-associated fatty liver disease, CD chow diet, HFD high-fat diet. Source data are available online for this figure

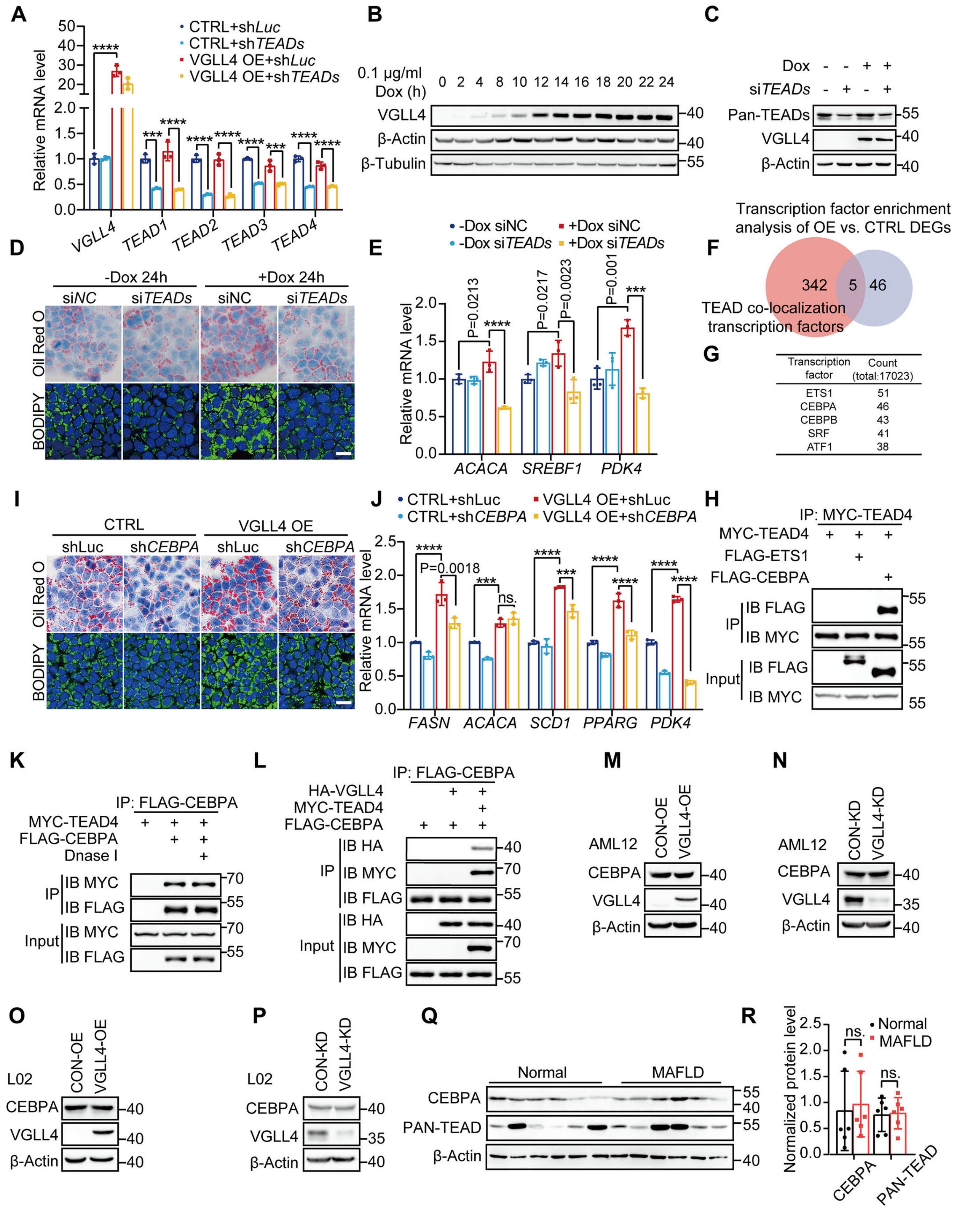
A
Relative mRNA level
CTRL+shLuc
CTRL+shTEADs
VGLL4 OE+shLuc
VGLL4 OE+shTEADs
VGLL4
TEAD1
TEAD2
TEAD3
TEAD4
B
0.1 μg/ml Dox (h)
0 2 4 8 10 12 14 16 18 20 22 24
VGLL4
β-Actin
β-Tubulin
C
Dox
siTEADs
Pan-TEADs
VGLL4
β-Actin
D
-Dox 24h
+Dox 24h
siNC
siTEADs
Oil Red O
BODIPY
E
-Dox siNC
+Dox siNC
-Dox siTEADs
+Dox siTEADs
Relative mRNA level
P=0.0213
P=0.0217
P=0.0023
P=0.001
ACACA
SREBF1
PDK4
F
Transcription factor enrichment analysis of OE vs. CTRL DEGs
342
5
46
TEAD co-localization transcription factors
G
Transcription factor
Count (total:17023)
ETS1 51
CEBPA 46
CEBPB 43
SRF 41
ATF1 38
I
CTRL
VGLL4 OE
shLuc
shCEBPA
Oil Red O
BODIPY
J
CTRL+shLuc
VGLL4 OE+shLuc
CTRL+shCEBPA
VGLL4 OE+shCEBPA
Relative mRNA level
P=0.0018
ns.
FASN
ACACA
SCD1
PPARG
PDK4
H
IP: MYC-TEAD4
MYC-TEAD4
FLAG-ETS1
FLAG-CEBPA
IP
Input
IB FLAG
IB MYC
K
IP: FLAG-CEBPA
MYC-TEAD4
FLAG-CEBPA
Dnase I
IP
Input
IB MYC
IB FLAG
L
IP: FLAG-CEBPA
HA-VGLL4
MYC-TEAD4
FLAG-CEBPA
IP
Input
IB HA
IB MYC
IB FLAG
M
AML12
CON-OE
VGLL4-OE
CEBPA
VGLL4
β-Actin
N
AML12
CON-KD
VGLL4-KD
CEBPA
VGLL4
β-Actin
O
L02
CON-OE
VGLL4-OE
CEBPA
VGLL4
β-Actin
P
L02
CON-KD
VGLL4-KD
CEBPA
VGLL4
β-Actin
Q
Normal
MAFLD
CEBPA
PAN-TEAD
β-Actin
R
Normalized protein level
Normal
MAFLD
ns.
CEBPA
PAN-TEAD

 © The Author(s)

Figure EV2. VGLL4 strengthens the TEAD4–CEBPA interaction in hepatosteatosis.

(**A**) RT-qPCR analysis of *VGLL4* and *TEAD1–4* mRNA levels in control or *VGLL4*-overexpressing L02 cells transduced with sh*Luc* or sh*TEADs*, with expression levels normalized to those of β-actin ($n = 3$). (**B**) Western blot analysis showing Dox-induced VGLL4 expression in the inducible *VGLL4*-overexpressing L02 cell line. Cells were treated with 0.1 μg/mL Dox for the indicated times. Samples were derived from the same experiment and blots were processed in parallel. (**C**) Western blot validation of siRNA-mediated *TEADs* knockdown and Dox-induced VGLL4 expression in inducible *VGLL4*-overexpressing L02 cells treated with or without Dox. Samples were derived from the same experiment and blots were processed in parallel. (**D**) Representative images of Oil Red O-stained cells and BODIPY 558/568 C_{12}-stained cells from the indicated groups after PA/OA treatment in the presence or absence of Dox (scale bar, 10 μm). (**E**) Relative mRNA levels of genes related to fatty acid metabolism and regulation in L02 cells from the indicated groups, with expression levels normalized to those of β-actin ($n = 3$). (**F**) Venn diagram of TEAD co-localizing transcription factors from ChIP-ATLAS and transcription factors enriched in *Vgll4*-overexpressing hepatocytes. (**G**) List of the intersection transcription factors from (**F**) and their regulating gene count in VGLL4-OE mice DEGs. (**H**) Co-immunoprecipitation analysis of the interactions between TEAD4 and ETS1 or CEBPA. (**I**) Representative images of Oil Red O-stained and BODIPY 558/568 C12-stained L02 cells from the indicated groups (scale bar, 10 μm). (**J**) Relative mRNA levels of genes related to fatty acid metabolism and regulation in L02 cells from the indicated groups, with expression levels normalized to those of β-actin ($n = 3$). (**K**) Co-immunoprecipitation assay to evaluate the interaction between CEBPA and TEAD4 in HEK293T cells, with or without DNase I treatment (to exclude DNA-mediated indirect binding). (**L**) Co-immunoprecipitation assay to examine the interaction between CEBPA and VGLL4 to determine whether the CEBPA–VGLL4 association depends on TEAD4 in HEK293T cells. (**M–P**) Western blot analysis of CEBPA protein levels in AML12 and L02 cells after *VGLL4/Vgll4* overexpression or knockdown. Samples were derived from the same experiment and blots were processed in parallel. (**Q**, **R**) Western blot analysis of CEBPA and TEAD protein levels in liver tissues from controls and patients with MAFLD (**Q**), with corresponding quantification normalized to β-actin (**R**) ($n = 6$). Samples were derived from the same experiment and blots were processed in parallel. Data information: Data are presented as mean ± SD. Representative technical replicates are shown in (**A**, **E**, **J**). For (**R**), significance was determined by Welch's *t* test. For (**A**, **E**, **J**), significance was determined using one-way ANOVA. $^{***}P < 0.001$, $^{****}P < 0.0001$, ns. indicates no significant difference. Source data are available online for this figure

© The Author(s)

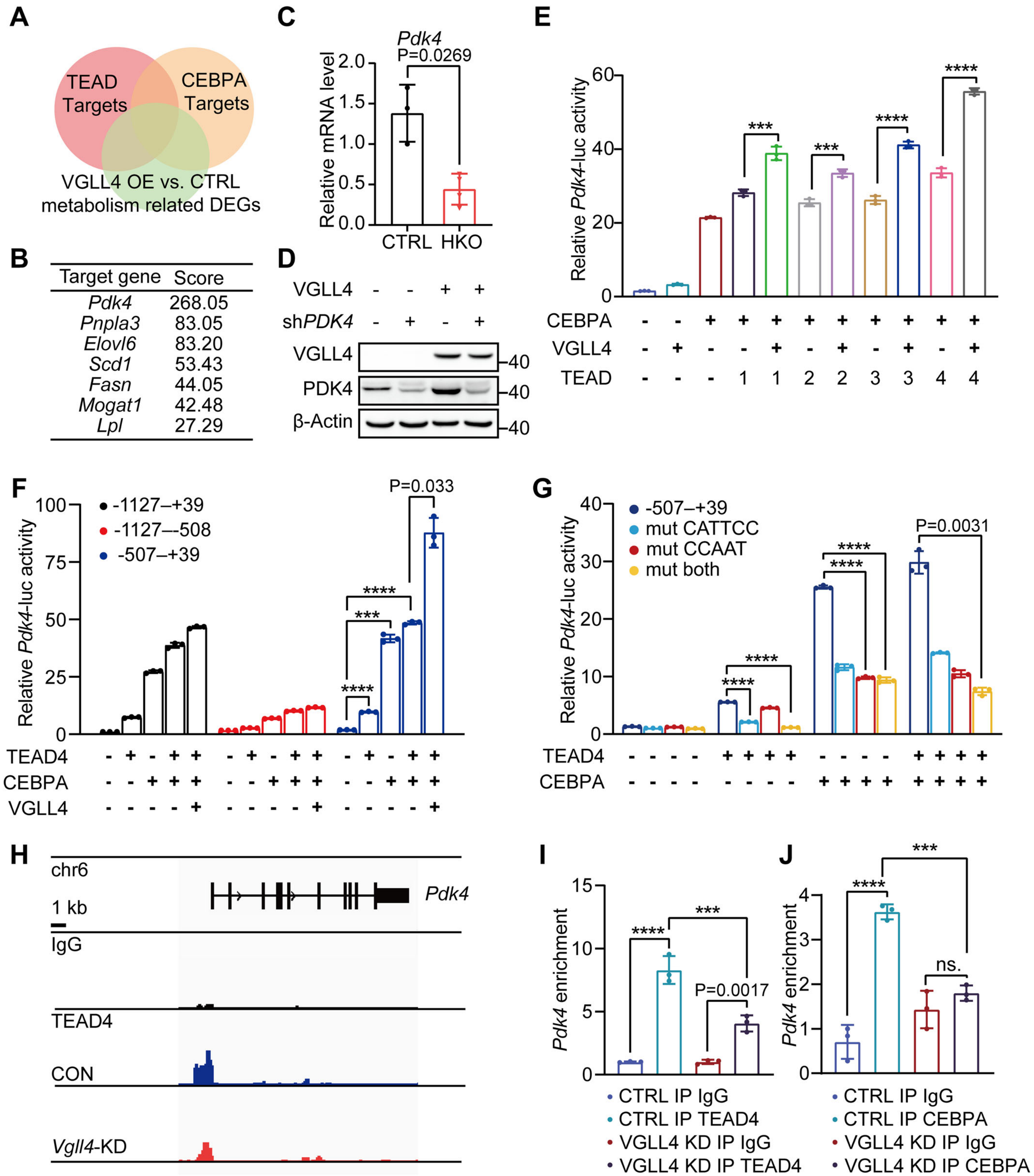
A
TEAD Targets
CEBPA Targets
VGLL4 OE vs. CTRL metabolism related DEGs
B
Target gene Score
Pdk4 268.05
Pnpla3 83.05
Elovl6 83.20
Scd1 53.43
Fasn 44.05
Mogat1 42.48
Lpl 27.29
C
Pdk4
P=0.0269
Relative mRNA level
CTRL HKO
D
VGLL4
shPDK4
PDK4
β-Actin
40
E
Relative Pdk4-luc activity
CEBPA
VGLL4
TEAD
F
-1127–+39
-1127–-508
-507–+39
P=0.033
Relative Pdk4-luc activity
TEAD4
CEBPA
VGLL4
G
-507–+39
mut CATTCC
mut CCAAT
mut both
P=0.0031
Relative Pdk4-luc activity
TEAD4
CEBPA
H
chr6
1 kb
Pdk4
IgG
TEAD4
CON
Vgll4-KD
I
Pdk4 enrichment
P=0.0017
CTRL IP IgG
CTRL IP TEAD4
VGLL4 KD IP IgG
VGLL4 KD IP TEAD4
J
Pdk4 enrichment
ns.
CTRL IP IgG
CTRL IP CEBPA
VGLL4 KD IP IgG
VGLL4 KD IP CEBPA

© The Author(s)

Figure EV3. VGLL4 promotes *Pdk4* transcription through TEAD4 and CEBPA.

(**A**) Venn diagram illustrating the strategy for selecting genes downstream of VGLL4-dependent TEAD/CEBPA regulation. (**B**) ChIP-ATLAS binding scores for candidate metabolic genes identified among DEGs in VGLL4-OE mice. Among the selected candidates, *Pdk4* showed the highest score, supporting its prioritization as a candidate gene with strong TEAD-binding evidence. (**C**) Relative mRNA levels of *Pdk4* in *Vgll4*-knockout and the corresponding control mice, with expression levels normalized to those of β-actin ($n = 3$–4). (**D**) Validation of the constructed L02 stable cell line. Samples were derived from the same experiment and blots were processed in parallel. (**E**) Luciferase reporter assay analyzing the transcriptional activity of the *Pdk4* promoter in L02 cells co-transfected with CEBPA, VGLL4, and different isoforms of the TEAD family ($n = 3$). (**F**) Luciferase reporter assays using serially truncated *Pdk4* promoter constructs in cells co-transfected with the indicated combinations of TEAD4, CEBPA, and VGLL4 ($n = 3$). (**G**) Luciferase reporter assays using the −507 to +39 *Pdk4* promoter reporter carrying point mutations in the predicted TEAD4-binding motif CATTCC, the predicted CEBPA-binding motif CCAAT, or both motifs ($n = 3$). (**H**) TEAD4 CUT&Tag tracks at the *Pdk4* locus in control and *Vgll4*-knockdown cells. (**I**, **J**) ChIP-qPCR validation of TEAD4 (**I**) and CEBPA (**J**) enrichment at the *Pdk4* promoter after *Vgll4* knockdown ($n = 3$). Data information: Data are presented as mean ± SD, representative technical replicates are shown in (**I**, **J**). Biological replicates are shown in (**C**, **E**–**G**). For (**C**), significance was determined by Welch's *t* test. For (**E**, **I**, **J**), significance was determined using one-way ANOVA. For (**F**, **G**), significance was determined using two-way ANOVA. ***$P < 0.001$, ****$P < 0.0001$, ns. indicates no significant difference. Source data are available online for this figure

© The Author(s)

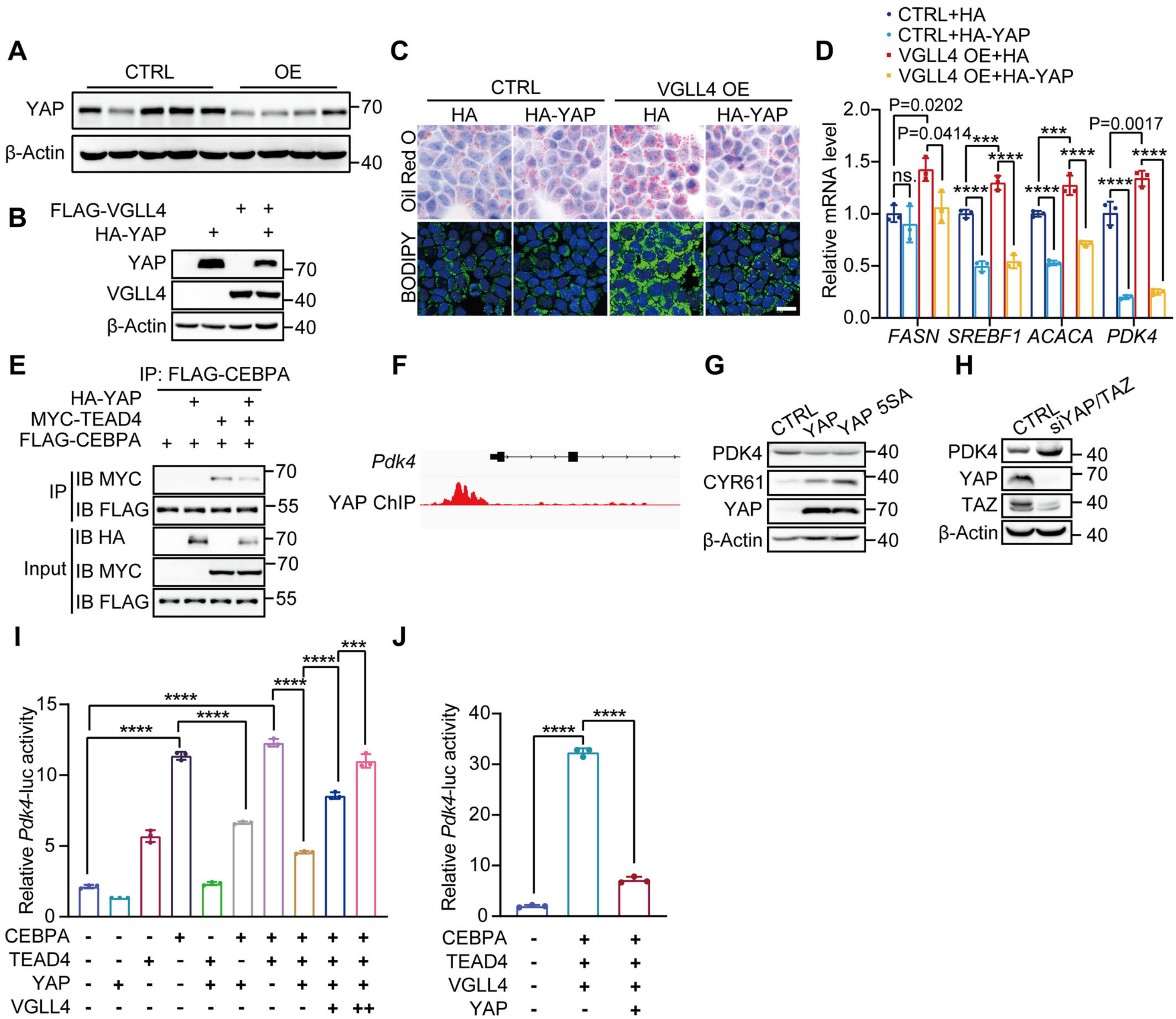

Figure EV4. VGLL4-dependent regulation of *Pdk4* by TEAD and CEBPA is antagonized by YAP.

(**A**) Western blot detection of YAP in CTRL and VGLL4-OE mice ($n = 4–5$). Samples were derived from the same experiment and blots were processed in parallel. (**B**) Validation of the constructed L02 stable cell line. Samples were derived from the same experiment and blots were processed in parallel. (**C**) Representative images of Oil Red O-stained and BODIPY 558/568 C12-stained L02 cells from the indicated groups (scale bar, 10 μm). (**D**) Relative mRNA levels of genes related to fatty acid metabolism and regulation in L02 cells from the indicated groups, with expression levels normalized to those of β-actin ($n = 3$). (**E**) Co-IP assay to assess the impact of YAP on the interaction between CEBPA and TEAD4 in HEK293T cells. (**F**) ChIP-seq data showing a YAP binding peak (GSM1331248) at the *Pdk4* promoter. (**G**, **H**) Western blot analysis of PDK4 and known YAP target proteins in YAP- or YAP-5SA-overexpressing and YAP/TAZ-knockdown L02 cells. Samples were derived from the same experiment and blots were processed in parallel. (**I**, **J**) Luciferase reporter assays were performed to analyze the transcriptional activity of the *Pdk4* promoter in L02 cells co-transfected with CEBPA, TEAD4, YAP and VGLL4 ($n = 3$). Data information: Data are presented as mean ± SD. Biological replicates are shown in (**I**, **J**). Representative technical replicates are shown in (**D**). For (**D**, **I**, **J**), significance was determined using one-way ANOVA. ***$P < 0.001$, ****$P < 0.0001$, ns. indicates no significant difference. Source data are available online for this figure

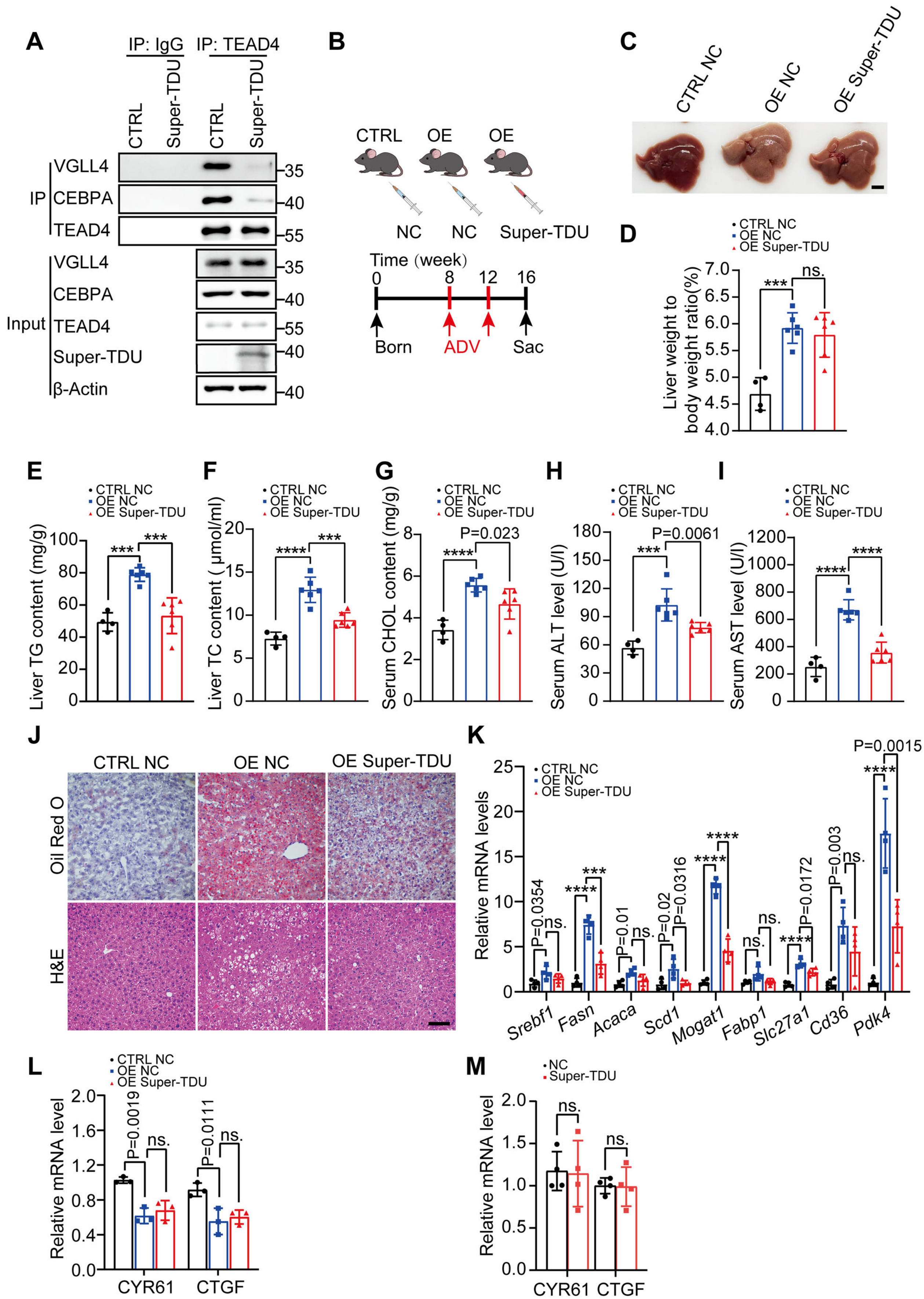
A
IP: IgG
IP: TEAD4
CTRL
Super-TDU
CTRL
Super-TDU
IP
VGLL4
CEBPA
TEAD4
Input
VGLL4
CEBPA
TEAD4
Super-TDU
β-Actin
B
CTRL
OE
OE
NC
NC
Super-TDU
Time (week)
0
8
12
16
Born
ADV
Sac
C
CTRL NC
OE NC
OE Super-TDU
D
CTRL NC
OE NC
OE Super-TDU
Liver weight to body weight ratio(%)
E
Liver TG content (mg/g)
F
Liver TC content (μmol/ml)
G
Serum CHOL content (mg/g)
P=0.023
H
Serum ALT level (U/l)
P=0.0061
I
Serum AST level (U/l)
J
CTRL NC
OE NC
OE Super-TDU
Oil Red O
H&E
K
Relative mRNA levels
P=0.0015
Srebf1
Fasn
Acaca
Scd1
Mogat1
Fabp1
Slc27a1
Cd36
Pdk4
L
Relative mRNA level
CYR61
CTGF
M
NC
Super-TDU
Relative mRNA level
CYR61
CTGF

Figure EV5. Administration of Super-TDU relieves hepatosteatosis induced by *Vgll4* overexpression.

(**A**) Endogenous TEAD4 Co-IP assay in L02 cells. Cellular extracts were subjected to IP with an anti-TEAD4 antibody or IgG control. IP samples were probed for coprecipitating endogenous proteins by immunoblot. Samples were derived from the same experiment and blots were processed in parallel. (**B**) Schematic diagram of animal experiments. OE mice were injected with ADV-Super-TDU (OE Super-TDU) or the corresponding ADV negative control vector (OE NC). CTRL mice received the ADV negative-control vector (CTRL NC), and metabolic phenotypes were subsequently assessed. (**C**) Representative images of the livers ($n = 3$). Scale bar, 5 mm. (**D–F**) Liver-to-body weight ratio (**D**), liver TG (**E**) and TC (**F**) from the indicated groups ($n = 4$–6). (**G–I**) Serum CHOL (**G**), ALT (**H**) and AST (**I**) from the indicated groups ($n = 4$–6). (**J**) Representative images of Oil Red O-stained (upper panel) and H&E-stained (lower panel) liver sections ($n = 3$). Scale bar, 100 µm. (**K–M**) Relative mRNA levels of genes from the indicated groups, with expression levels normalized to those of β-actin ($n = 3$–4). Data information: Data are presented as mean ± SD. Biological replicates are shown in (**D–I**, **K–M**). For (**M**), significance was determined by Welch's *t* test. For (**D–I**, **K**, **L**), significance was determined using one-way ANOVA. ***$P < 0.001$, ****$P < 0.0001$, ns. indicates no significant difference. TG triglyceride, CHOL cholesterol. Source data are available online for this figure

 © The Author(s)
