# Supplementary material for: VGLL4 enhances PDK4 transcription and promotes metabolic dysfunction-associated fatty liver disease
Source: EMBO Rep. 2026 Sep 22;27(20):5807–36. doi: 10.1038/s44319-026-00934-x (PMC13694279; doi:10.1038/s44319-026-00934-x)
Supplement: Supplementary file 2 [file 44319_2026_934_MOESM1_ESM.pdf]

Appendix

VGLL4 enhances PDK4 transcription and promotes metabolic dysfunction-associated fatty liver disease

Chen et al.

Contents

Appendix Figure S1 2

Appendix Figure S2 3

Appendix Figure S3 4

Appendix Figure S4 5

Appendix Figure S5 6

Appendix Figure S6 7

Appendix Table S1 9

Appendix Figure S1 Hepatocyte-specific *Vgll4* overexpression induces hepatic steatosis.

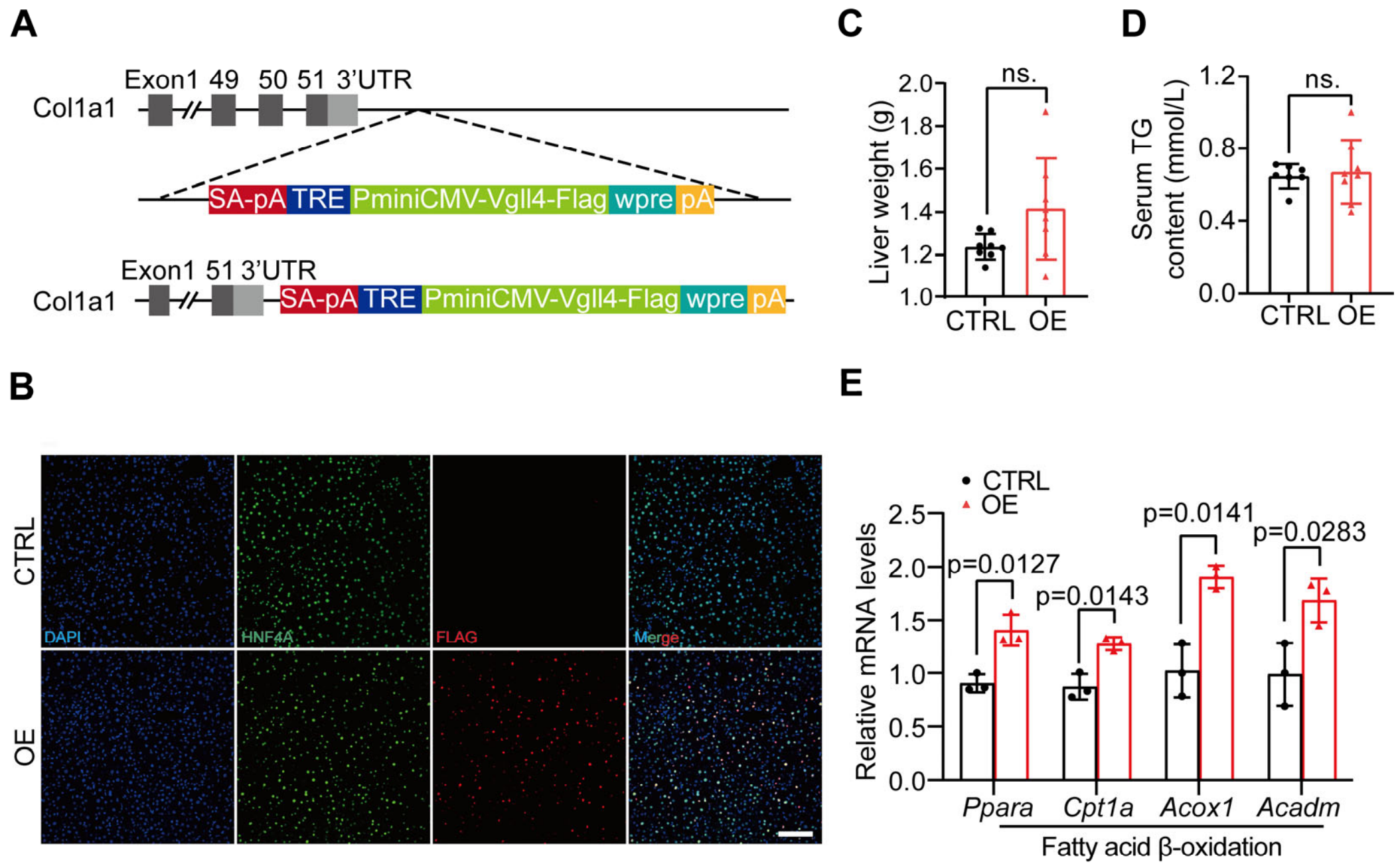

Appendix Figure S1

(A) Schematic diagram illustrating the generation of *Col1a1-Teto-Vgll4-Flag-wpre-polyA* mice. (B) Immunofluorescence staining for FLAG-VGLL4 and HNF4A in liver tissues from control and *Vgll4* overexpression mice (scale bar, 50 μm) (n=3). (C) Liver weights of mice in the indicated groups (n=8). (D) Serum triglyceride levels in mice in the indicated groups (n=7–8). (E) The mRNA levels of genes involved in fatty acid β-oxidation, with expression levels normalized to those of β-actin (n=3). ns. indicates no significant difference. Data are presented as mean ± SD. Biological replicates are shown in panels C, D and E. For (C–E), significance was determined by Welch's t-test. Abbreviations: OE, hepatocyte-specific VGLL4 overexpression.

Appendix Figure S2 Hepatocyte-specific *Vgll4* overexpression induces inflammation.

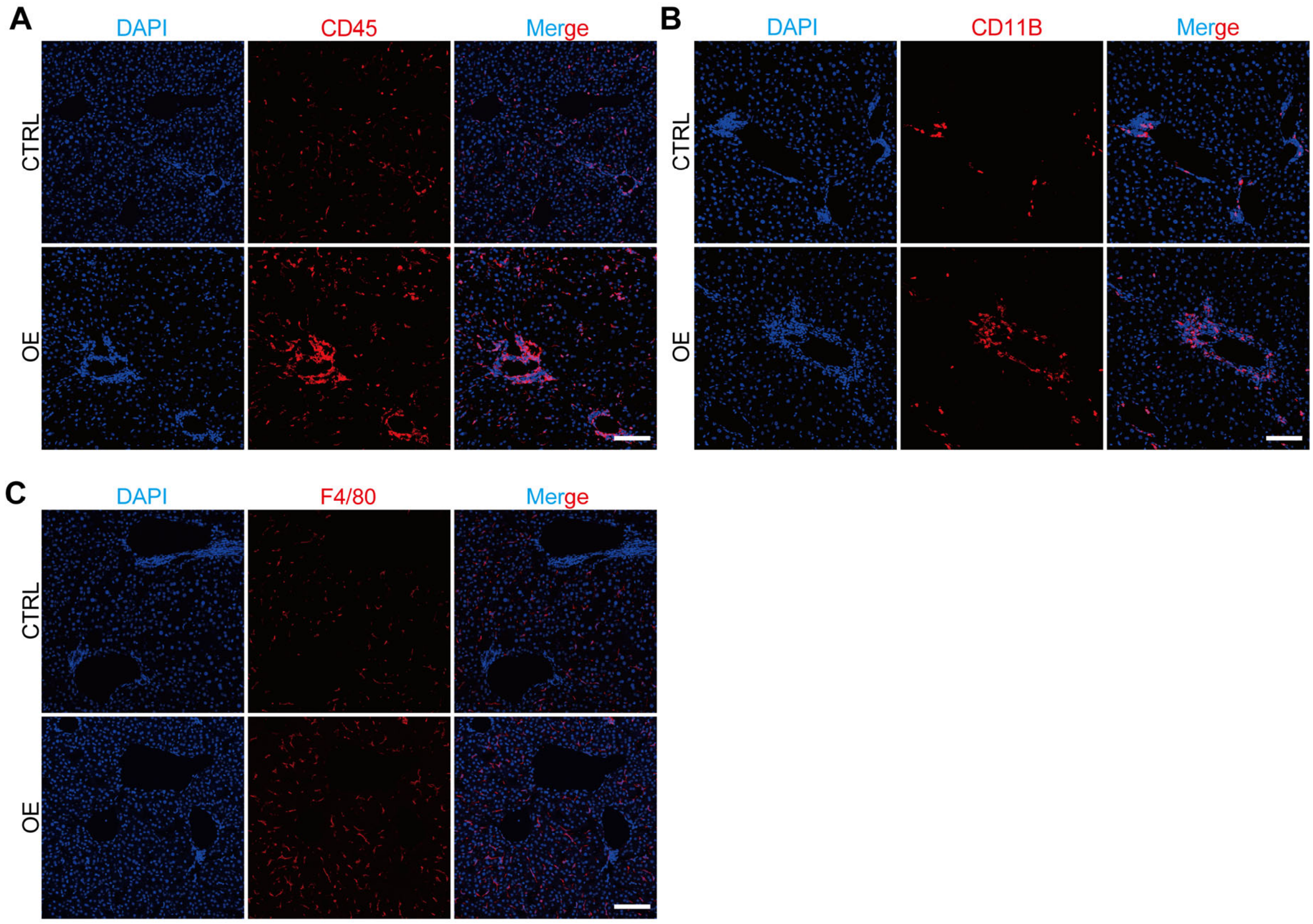

Appendix Figure S2

(A–C) Immunofluorescence staining in liver tissues from control and VGLL4 overexpression mice. CD45 (marker for leukocytes, red) with DAPI (nuclear stain, blue); merged images show overlay (A). CD11B (marker for myeloid cells, red) with DAPI (blue); merged images show overlay (B). F4/80 (marker for macrophages, red) with DAPI (blue); merged images show overlay (C). Each image is representative of three independent experiments. Scale bar represents 100 μm.

Appendix Figure S3 Adenovirus-mediated *Vgll4* overexpression in adult mice recapitulates hepatic steatosis and impaired insulin signaling.

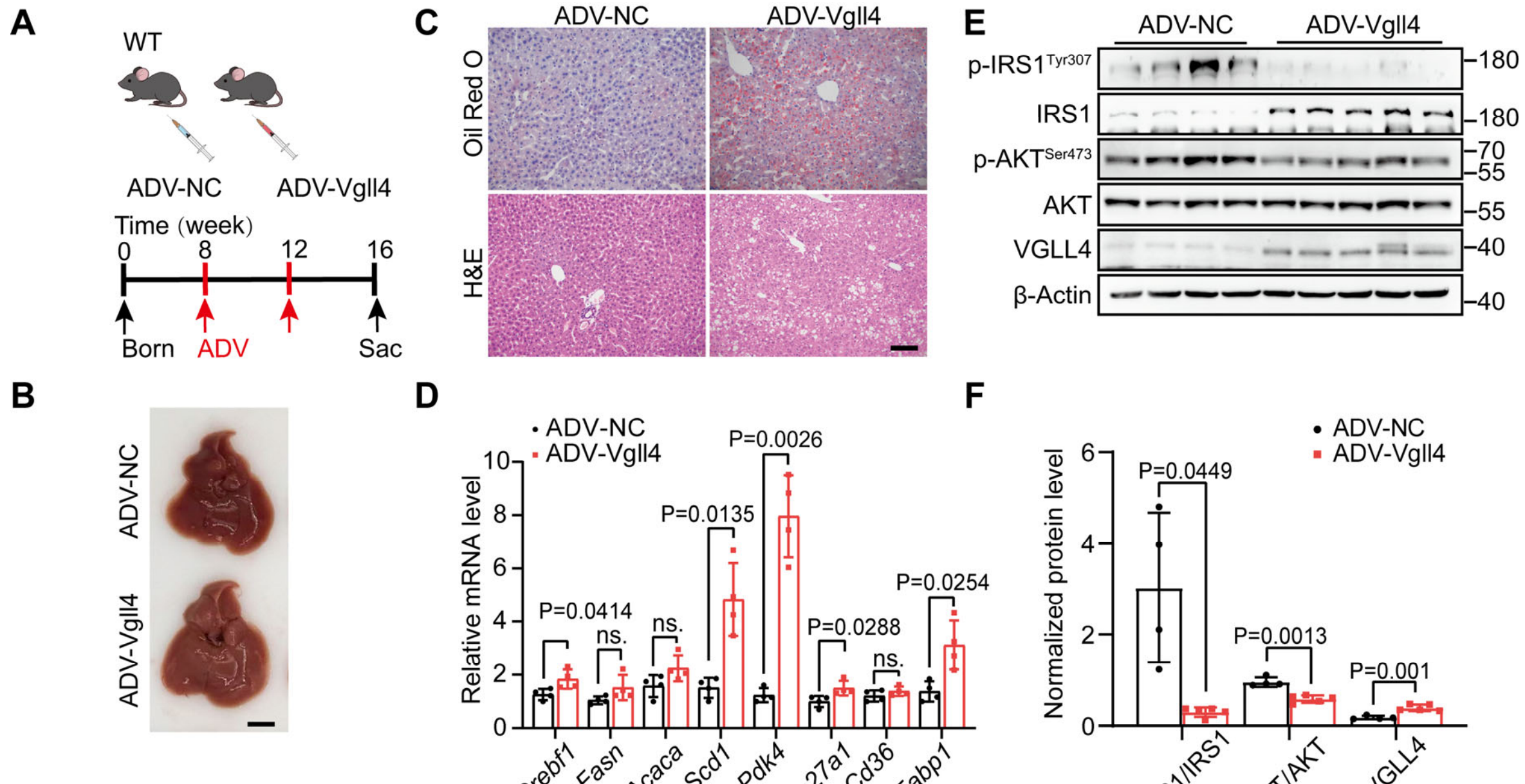

Appendix Figure S3

(A) Schematic diagram of animal experiments. WT mice were injected with ADV-Vgll4 or ADV negative control vector (ADV-NC) through the tail vein, and the metabolic phenotype was assessed. (B) Representative images of the livers (n=3). Scale bar, 5 mm. (C) Representative images of Oil Red O-stained (upper panel) and H&E-stained (lower panel) liver sections (n=3). Scale bar, 100 μm. (D) The mRNA levels of genes involved in fatty acid synthesis and uptake from the indicated groups, with expression levels normalized to those of β-actin (n=4). (E, F) Mice were intraperitoneally injected with insulin after 4 h of fasting. Activation of the insulin signaling pathway in the livers of mice from the indicated groups was detected by Western blotting (E), and protein levels of insulin signaling-related molecules were normalized to β-actin (F) (n=4–5). Samples were derived from the same experiment and blots were processed in parallel. Data are presented as mean ± SD. Biological replicates are shown in panel D. For (D) and (F), significance was determined by Welch’s t-test. ns. indicates no significant difference.

Appendix Figure S4 Hepatic *Vgll4* deletion attenuates HFD-induced hepatic steatosis.

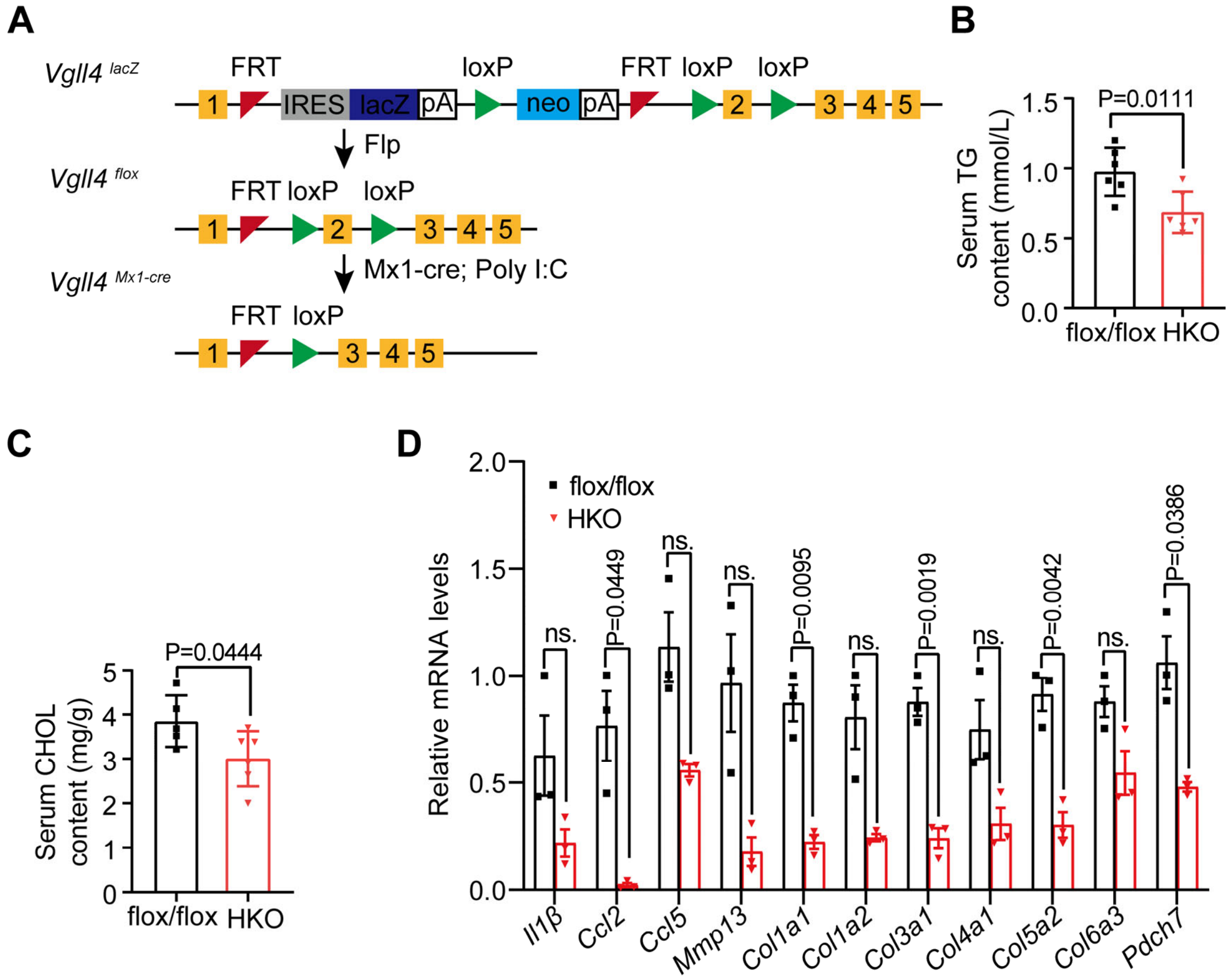

Appendix Figure S4

(A) Schematic diagram illustrating the generation of *Vgll4* $^{Mx1\text{-}cre}$ mice (HKO). (B, C) Serum triglyceride (B) and cholesterol (C) of mice in the indicated groups (n=5–6). (D) The relative mRNA levels of genes related to liver inflammation and fibrosis from the indicated groups, with expression levels normalized to those of β-actin (n=3). Data are presented as mean ± SD. Biological replicates are shown in panels B–D. For (B–D), significance was determined by Welch's t-test. ns. indicates no significant difference. Abbreviations: TG, triglyceride; CHOL, cholesterol.

Appendix Figure S5 AAV2/8-TBG-Cre-mediated *Vgll4* deletion protects adult mice from HFD-induced hepatic steatosis and impaired insulin signaling.

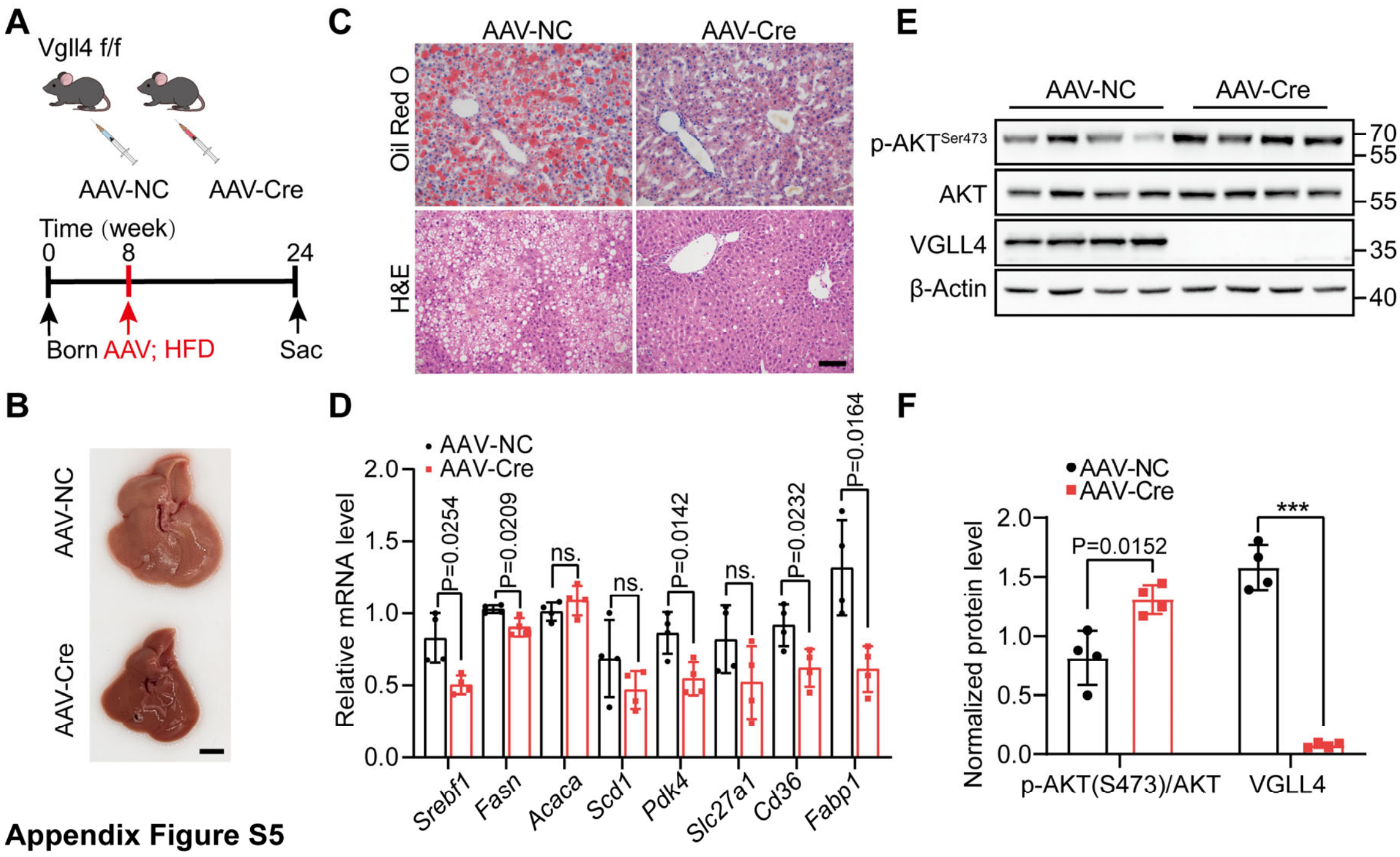

Appendix Figure S5

(A) Schematic diagram of animal experiments. *Vgll4$^{f/f}$* mice were injected with AAV2/8-TBG-Cre (AAV-Cre) or AAV negative control vector (AAV-NC) through the tail vein, and the metabolic phenotype was assessed. (B) Representative images of the livers (n=3). Scale bar, 5 mm. (C) Representative images of Oil Red O-stained (upper panel) and H&E-stained (lower panel) liver sections (n=3). Scale bar, 100 μm. (D) The mRNA levels of genes involved in fatty acid synthesis and uptake from the indicated groups, with expression levels normalized to those of β-actin (n=4). (E, F) Mice were intraperitoneally injected with insulin after a 4-h fast. Activation of the insulin signaling pathway in the livers of mice from the indicated groups was detected by Western blotting (E), and protein levels of insulin signaling-related molecules were normalized to β-actin (F) (n=4). Samples were derived from the same experiment and blots were processed in parallel. Data are presented as mean ± SD. Biological replicates are shown in panel D. For (D) and (F), significance was determined by Welch's t-test. ***p < 0.001, ns. indicates no significant difference.

Appendix Figure S6 VGLL4 positively regulates PA/OA-induced hepatosteatosis in vitro.

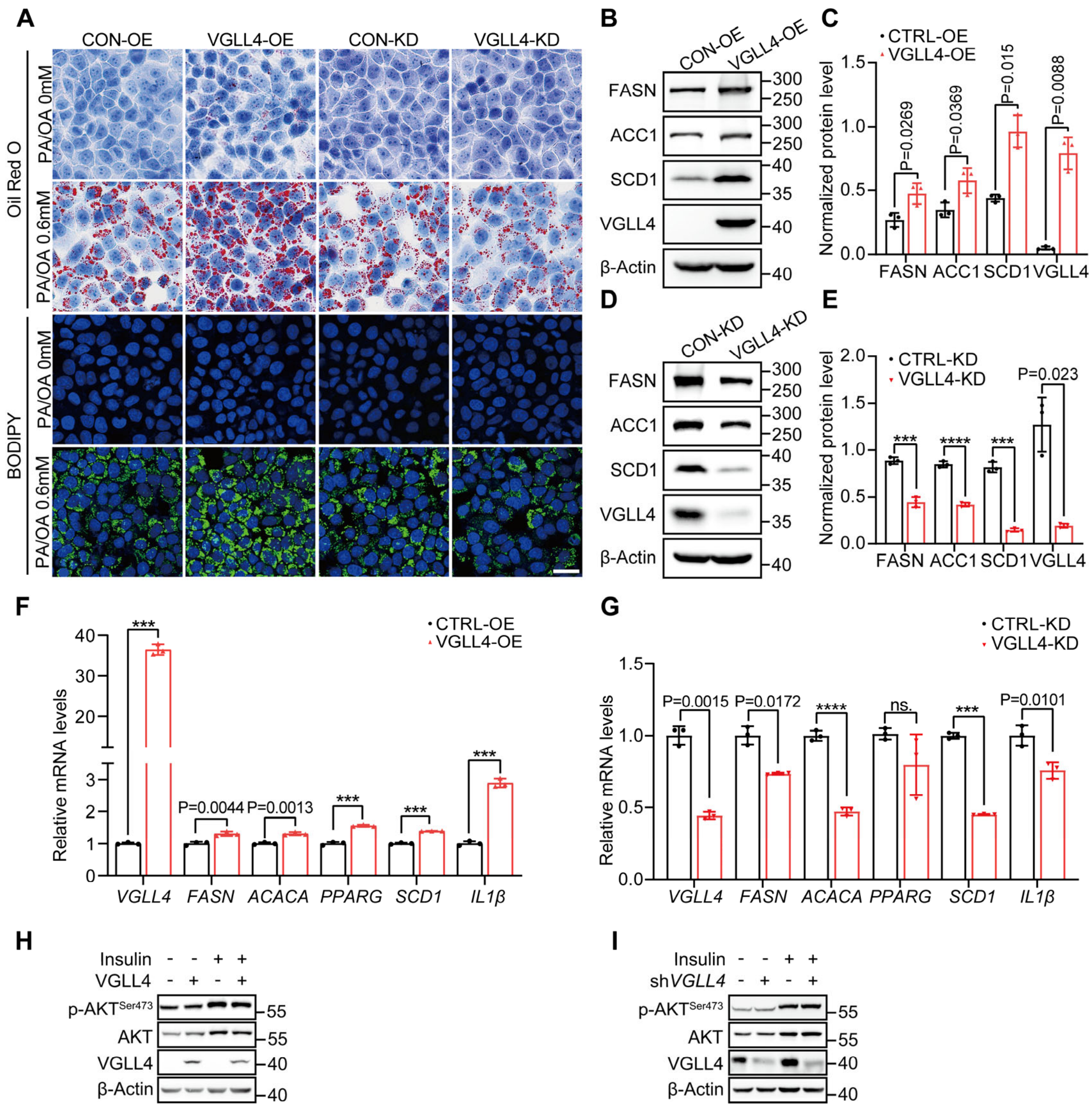

Appendix Figure S6

(A) *VGLL4*-overexpressing (VGLL4-OE) and corresponding control (CON-OE) L02 cells, and *VGLL4*-knockdown (VGLL4-KD) and corresponding control (CON-KD) L02 cells, were stimulated with PA/OA at 0 or 0.6 mM. The images presented include representative Oil Red O-stained (first and second panels) and BODIPY™ 558/568 C12-stained (third and fourth panels) L02 cells from the specified groups (scale bar, 10 μm). Data shown are representative of at least three independent experiments. (B–E) L02 cells from the indicated groups were assayed by Western blotting, and the protein levels were normalized to β-actin (n=3). Samples were derived from the same experiment

and blots were processed in parallel. (F, G) The relative mRNA levels of genes related to fatty acid metabolism and hepatic inflammation in L02 cells from the indicated groups, with expression levels normalized to those of β-actin (n=3). (H, I) Western blot detection of p-AKT^{Ser473}, AKT, and VGLL4 in L02 cells with *VGLL4* overexpression (H) or knockdown (I) treated with or without insulin. Samples were derived from the same experiment and blots were processed in parallel. Data are presented as mean ± SD, representative technical replicates are shown in panels F and G. For (C), (E–G), significance was determined by Welch's t-test. ***p < 0.001, ****p < 0.0001, ns. indicates no significant difference. Abbreviation: PA, palmitic acid; OA, oleic acid.

Appendix Table S1 The clinical characteristics of MAFLD patients and normal control patients.

No.	Type	Sex	Age (years)	BMI (kg/m2)	ALT (U/L)	AST (U/L)	TC (mmol/L)	TG (mmol/L)	LDL (mmol/L)	HDL (mmol/L)
#1	MAFLD	Male	34	26.23	25	15	5.55	2.18	3.69	0.87
#2	MAFLD	Male	73	24.16	28	32	7.3	2.23	4.91	1.38
#3	Normal	Male	57	21.7	-	-	4.87	0.97	3.03	1.40
#4	Normal	Male	54	20.57	7	14	-	-	-	-
#5	MAFLD	Male	57	22.3	15	18	5.14	1.19	3.68	0.92
#6	MAFLD	Male	49	26.4	49	25	4.20	1.10	2.99	0.75
#7	Normal	Male	46	22.5	-	-	-	1.95	-	6.36
#8	Normal	Male	53	22.49	21	25	3.75	1.23	2.47	0.72
#9	Normal	Male	58	23.88	23	19	3.19	0.79	1.96	0.87
#10	MAFLD	Male	40	-	21	29	4.14	1.76	2.23	1.11
#11	Normal	Male	64	24.86	-	-	-	-	2.73	1.15
#12	MAFLD	Male	49	27.14	23	20	5.90	3.09	3.48	1.02

Abbreviations: BMI, body mass index; AST, aspartate aminotransferase; ALT, alanine aminotransferase; TG, triglyceride; TC, Total cholesterol; HDL, high density lipoprotein; and LDL, low density lipoprotein.
