# Supplementary material for: VGLL4 enhances PDK4 transcription and promotes metabolic dysfunction-associated fatty liver disease
Source: EMBO Rep. 2026 Sep 22;27(20):5807–36. doi: 10.1038/s44319-026-00934-x (PMC13694279; doi:10.1038/s44319-026-00934-x)
Supplement: Supplementary file 3 [file 44319_2026_934_MOESM2_ESM.pdf]

EMBO reports

VGLL4 enhances PDK4 transcription and promotes metabolic dysfunction-associated fatty liver disease

Yixing Chen, Xiaodong Wang, Wentao Yu, Meixi Gong, Yi Lu, Jinjin Xu, Ruizeng Yang, Haoran Gao, Zhuoqun Gao, Jiayan Yan, Zhifeng Jiang, Shiyu Zhang, Xueyan Ma, Jing Yu, Fang Wei, Qiang Fu, Yi Zhou, Jian Zhou, Jian Wan, Yunyun Jin, Lei Zhang, Haoen Zhang, and Hao Zhang

Corresponding author(s): Lei Zhang (rayzhang@sjtu.edu.cn) , Yunyun Jin (yunyunjin@sjtu.edu.cn), Jian Wan (wanjian@shpdph.com)

Review Timeline:

Submission Date:	23rd Jan 26
Editorial Decision:	12th Mar 26
Revision Received:	8th Jun 26
Editorial Decision:	15th Jul 26
Revision Received:	11th Aug 26
Accepted:	2nd Sep 26

Editor: Achim Breiling

Transaction Report:

(Note: With the exception of the correction of typographical or spelling errors that could be a source of ambiguity, letters and reports are not edited. Depending on transfer agreements, referee reports obtained elsewhere may or may not be included in this compilation. Referee reports are anonymous unless the Referee chooses to sign their reports.)

Dear Dr. Zhang,

Thank you for the transfer of your manuscript to EMBO reports. I have now received reports from the three referees that were asked to evaluate your study, which can be found at the end of this email. As you will see, the referees have several comments, concerns, and suggestions, indicating that a major revision of the manuscript is necessary to allow publication of the study in EMBO reports. As the reports are below, and all points need to be addressed, I will not detail them here.

Given the constructive referee comments, I would like to invite you to revise your manuscript with the understanding that the concerns of the referees must be addressed in the revised manuscript and in a detailed point-by-point response. Acceptance of your manuscript will depend on a positive outcome of a second round of review. It is EMBO reports policy to allow a single round of revision only and acceptance of the manuscript will therefore depend on the completeness of your responses included in the next, final version of the manuscript.

Revised manuscripts should be submitted within three months of a request for revision. Please contact me to discuss the revision (also by video chat) if you have questions or comments regarding the revision, or should you need additional time.

When submitting your revised manuscript, please also carefully review the instructions that follow below.

PLEASE NOTE THAT upon resubmission revised manuscripts are subjected to an initial quality control prior to exposition to re-review. Upon failure in the initial quality control, the manuscripts are sent back to the authors, which may lead to delays. Frequent reasons for such a failure are the lack of the data availability section (please see below) and the presence of statistics based on n=2 (the authors are then asked to present scatter plots or provide more data points).

When submitting your revised manuscript, we will require:

1) a .docx formatted version of the final manuscript text (including legends for main figures, EV figures and tables), but without the figures included. Figure legends should be compiled at the end of the manuscript text.

2) individual production quality figure files as .eps, .tif, .jpg (one file per figure), of main figures and EV figures. Please upload these as separate, individual files upon re-submission.

The Expanded View format, which will be displayed in the main HTML of the paper in a collapsible format, has replaced the Supplementary information. You can submit up to 6 images as Expanded View. Please follow the nomenclature Figure EV1, Figure EV2 etc. The figure legend for these should be included in the main manuscript document file in a section called Expanded View Figure Legends after the main Figure Legends section. Additional Supplementary material should be supplied as a single pdf file labeled Appendix. The Appendix should have page numbers and needs to include a table of content on the first page (with page numbers) and legends for all content. Please follow the nomenclature Appendix Figure Sx, Appendix Table Sx etc. throughout the text, and also label the figures and tables according to this nomenclature.

For more details, please refer to our guide to authors:
https://link.springer.com/journal/44319/submission-guidelines#cms-Revised-submissions

Please consult our guide for figure preparation:
https://media.springernature.com/original/springer-cms/rest/v1/content/27825798/data/v1

3) a .docx formatted letter INCLUDING the reviewers' reports and your detailed point-by-point responses to their comments. As part of the EMBO Press transparent editorial process, the point-by-point response is part of the Review Process File (RPF), which will be published alongside your paper.

4) a complete author checklist, which you can download from our author guidelines (https://link.springer.com/journal/44319/submission-guidelines#cms-Revised-submissions). Please insert page numbers in the checklist to indicate where the requested information can be found in the manuscript. The completed author checklist will also be part of the RPF.

Please also follow our guidelines for the use of living organisms, and the respective reporting guidelines:
https://link.springer.com/partners/embo-press/editorial-policies#Animal%20and%20human%20studies

5) that primary datasets produced in this study (e.g. RNA-seq, ChIP-seq, structural and array data) are deposited in an appropriate public database. If no primary datasets have been deposited, please also state this in a dedicated section (e.g. 'No primary datasets have been generated and deposited'), see below.

See also: https://link.springer.com/partners/embo-press/editorial-policies#Data%20deposition

Please remember to provide a reviewer password if the datasets are not yet public.

The accession numbers and database should be listed in a formal "Data Availability" section that follows the model below. This is now mandatory (like the COI statement). Please note that the Data Availability Section is restricted to new primary data that are part of this study. This section is mandatory. As indicated above, if no primary datasets have been deposited, please state this in this section

Data availability

The datasets produced in this study are available in the following databases:

- RNA-Seq data: Gene Expression Omnibus GSE46843 (https://www.ncbi.nlm.nih.gov/geo/query/acc.cgi?acc=GSE46843)
- [data type]: [name of the resource] [accession number/identifier/doi] ([URL or identifiers.org/DATABASE:ACCESSION])

*** Note - All links should resolve to a page where the data can be accessed. ***

See also: https://link.springer.com/partners/embo-press/editorial-policies#Data%20availability%20statement

Moreover, I have these editorial requests:

6) We now request the publication of original source data with the aim of making primary data more accessible and transparent to the reader. You will receive a separate email with instructions for providing source data with your revised manuscript, including information how to upload and organize the files.

7) Our journal encourages inclusion of *data citations in the reference list* to directly cite datasets that were re-used and obtained from public databases. Data citations in the article text are distinct from normal bibliographical citations and should directly link to the database records from which the data can be accessed. In the main text, data citations are formatted as follows: "Data ref: Smith et al, 2001" or "Data ref: NCBI Sequence Read Archive PRJNA342805, 2017". In the Reference list, data citations must be labeled with "[DATASET]". A data reference must provide the database name, accession number/identifiers and a resolvable link to the landing page from which the data can be accessed at the end of the reference. Further instructions are available at: https://link.springer.com/journal/44319/submission-guidelines#cms-Reference-guidelines

8) Regarding data quantification and statistics, please make sure that the number "n" for how many independent experiments were performed, their nature (biological versus technical replicates), the bars and error bars (e.g. SEM, SD) and the test used to calculate p-values is indicated in the respective figure legends (also for EV and Appendix figures). Please also check that all the p-values are explained in the legend, and that these fit to those shown in the figure. Please provide statistical testing where applicable. Please avoid the phrase 'independent experiment' but clearly state if these were biological or technical replicates. Please also indicate (e.g. with n.s.) if testing was performed, but the differences are not significant. In case n=2, please show the data as separate datapoints without error bars and statistics. See also:
https://link.springer.com/journal/44319/submission-guidelines#cms-Figure-and-data-presentation

If n<5, please show single datapoints for diagrams.

9) Please add scale bars of similar style and thickness to microscopic images, using clearly visible black or white bars (depending on the background). Please place these in the lower right corner of the images themselves. Please do not write on or near the bars in the image but define the size in the respective figure legend.

10) Please also note our reference format:
https://link.springer.com/journal/44319/submission-guidelines#cms-Reference-guidelines

11) We updated our journal's competing interests policy in January 2022 and request authors to consider both actual and perceived competing interests. Please name this section 'Disclosure and Competing Interests Statement' and put it after the Acknowledgements section.
See: https://link.springer.com/partners/embo-press/editorial-policies#Competing%20interest%20disclosures

12) We now use CRediT to specify the contributions of each author in the journal submission system. CRediT replaces the author contribution section. Please use the free text box to provide more detailed descriptions and do NOT provide your final manuscript text file with an author contributions section. See also our guide to authors (section 'Author contributions'):
https://link.springer.com/journal/44319/submission-guidelines#cms-Revised-submissions

13) All Materials and Methods need to be described in the main text using our 'Structured Methods' format, which is required for all research articles. According to this format, the Methods section should include a Reagents and Tools Table (listing key reagents, experimental models, software, and relevant equipment and including their sources and relevant identifiers), uploaded

as separate file, and a Methods section in which we encourage the authors to describe their methods using a step-by-step protocol format with bullet points, to facilitate the adoption of the methodologies across labs. More information on how to adhere to this format as well as downloadable templates (.doc) for the Reagents and Tools Table can be found in our author guidelines (section 'Structured Methods'):

https://link.springer.com/journal/44319/submission-guidelines#cms-Manuscript-organisation-and-formatting

Please add all primer or antibody information directly to the Reagents and Tools table.

14) Please add up to five keywords and order the manuscript sections like this, using only these names:
Title page - Abstract (max. 175 words) - Keywords (max. 5) - Introduction - Results - Discussion - Methods - Data availability section - Acknowledgements (please include here all the funding information) - Disclosure and Competing Interests Statement - References - Figure legends - Expanded View Figure legends

Please also provide a title with as little abbreviations as possible.

15) Please make sure that all the funding information is also entered into the online submission system and that it is complete and similar to the one in the acknowledgement section of the manuscript text file.

16) Please also confirm that for all Western blot panels shown the loading control was run on the same gel as the other proteins detected. Please note that we discourage comparisons between samples on different gels/blots, even if the samples derive from one experiment, as confounding factors reduce comparability. If unavoidable, the figure legend must state that the samples derive from the same experiment and that gels/blots were processed in parallel. If a 'representative' loading control is shown for multiple gels/blots, the intra-gel controls should be shown in the source data files, and the figure legends should describe the data displayed accurately. See our author guidelines:

https://link.springer.com/journal/44319/submission-guidelines#cms-Figure-and-data-presentation (section 'Electrophoretic gels and blots').

We would also welcome the submission of cover suggestions, or motifs to be used by our Graphics Illustrator in designing a cover.

As part of the EMBO publication's Transparent Editorial Process, EMBO reports publishes online a Review Process File (RPF) to accompany accepted manuscripts. This File will be published in conjunction with your paper and will include the referee reports, your point-by-point response and all pertinent correspondence relating to the manuscript.

You are able to opt out of this by letting the editorial office know (emboreports@embo.org). If you do opt out, the Review Process File link will point to the following statement: "No Review Process File is available with this article, as the authors have chosen not to make the review process public in this case."

I look forward to seeing a revised form of your manuscript when it is ready.

Yours sincerely,

Achim Breiling
Senior Editor
EMBO Reports

Referee #1:

This manuscript by Yixing Chen and colleagues represent a very solid amount of work that highlights the effect of VGLL4 on lipid metabolism in hepatocytes with possible relevance to MASLD. The quantity of work that has been done is impressive, including overexpression and knock out studies in vivo and in cell systems.

Overall, in my opinion, this is a super solid work and the authors have to be congratulated for the amount of high quality work.

The authors have acknowledged that they have not identified how VGLL4 becomes overexpressed in the context of MASLD which would have been interesting to know.
In my opinion, there is one other major limitation to this study. The authors show that VGLL4 influences the expression of many genes including lipogenic genes. But they claim that VGLL4/Tead/CEBPA modulates steatosis by regulating the transcription of PDK4. I am not sure to understand why can they claim that VGLL4/Tead/CEBPA specifically regulate PDK4 and not a broader

range of genes involved in metabolic homeostasis. If PDK4 is the responsible for the effect on steatosis, how does PDK4 modulation influence the whole transcriptome including lipogenic genes?

A minor remark, given that lipid accumulation is a major observation in this study, a more detailed analysis of changes in tissue lipid and cell lipid composition would have been interesting.

Finally, the authors used the term MAFLD and NASH... I am not sure but it might be better to use MASLD and MASH? Please check what would the most suitable nomenclature.

Altogether, I would like to let the authors know that given the amount of work they have performed, they should not consider my review as requiring mandatory extra experiments.

Referee #2:

In this manuscript Chen et al. identify that VGLL4 is induced in MAFLD. Using mouse models they go on to show that VGLL4 oe in the liver induces hepatic steatosis and other phenotypes (glucose intolerance, insulin resistance), and that VGLL4 KO makes the mice resistant to these changes after HFD.
The authors propose that a trimeric complex of TEAD/CEBPA/VGLL4 (TCV) directly upregulates PDK4 expression which they propose to be the critical target gene for the observed phenotypes.

In general, the manuscript addresses a topic of high relevance. Also the use of Sper-TDU is very interesting and shows some translational value of their findings. The authors mostly use appropriate tools to address their research question.
However, several weaknesses (particularly with regard to the proposed mechanism), which are outlined in detail below, dampen this reviewer's enthusiasm.

General:
This reviewer is not yet convinced regarding the proposed linear pathway of the putative TEAD/CEBPA/VGLL4 (TCV) complex directly regulating PDK4. The data presented would also be consistent with a parallel model: TCV regulates metabolism via genes XYZ and this can be normalized by downregulating the master regulator PDK4. This part would need to be substantially bolstered by additional experiments, e.g. ChIP-Seq/CUT&RUN (specified below).

Statistics:
- The authors should rather use a Welch than a Student's t-test
- For repeated measurements (e.g. insulin resistance) a two-way ANOVA is more appropriate

Fig. 1:
- (How long have the mice been on doxycycline food? What is th control group?)

Fig. S1B/C:
The upregulation seems rather minor (only 1 shows strong, 2 show weak upregulation - 3 none). The authors should do co-stainings with HNF4a to demonstrate that VGLL4 upregulation occurs in hepatocytes. The presented data is not convincing

Fig. 2:
Are the changes, e.g. in 2C, related to the steatosis phenotype or to VGLL4 oe?. The authors should analyze these proteins after VGLL4 induction when no steatosis has developed yet.

Fig. 3:
VGLL4 fl/fl mice as control without the transgene (Cre) is not an ideal control. Better control animals would fl/+; cre/+ or +/+; cre/+
Using Mx1-Cre for "liver-specific" KOs is very unusual and not very clean. Mx1-Cre is also highly active in hematopoeitic cells which could confound the analyses here. At least, the authors should discuss this shortcoming and make the reader aware of this.
Ideally, rather use Alb-CreERT or Adeno-Cre.

Fig. 4:
4A : Please provide a quantification with proper statistical test (one-way ANOVA)
How acute are the gene expression changes. Please use an inducible system to analyze this. If oe persists over weeks, the effects could be due to selection processes as compared to gene regulation.

4H: Please provide a schematic showing which domains are deleted in CEBPA.

4I: I find the argument regarding the DNA dependency hard to follow. The authors claim that TEAD and CEBPA bind to joint

targets. By using DNAse I they simply demonstrate that the PPI is independent of DNA (which is crucial). However, if CEPBA binds to the TEAD DNA binding domain, it could also interfere with TEAD's ability to bind to DNA?
The authors should also analzye whether VGLL4 oe/KD has an acute impact on CEBPA protein expression in the L02 cell line.

Fig. 5:
5A:-C: If the model is that CEBPA and TEAD bind to joint target genes (e.g.) and this interaction is stabilized by VGLL4. This needs to be shown in a genome-wide ChIP-Seq/CUT&RUN experiment using shCtrl vs. shVGLL4. Please use spike-in controls for normalization here.

5F: The authors need to show the protein expression of PDK4. Does shPDK4 bring dwon PDK4 expression to control conditions (Ctrl / sh Luc).
Based on their model, the PDK4 expression should go up after VGLL4 oe. This per se, is against the current model of how VGLL4 acts in conjunction with TEAD-YAP/TAZ: it normally acts as a repressor (this would need be addressed by the authors). Furthermore, there is only very little - and no strong evidence - that YAP can act as a repressor (related to Fg. S7)

5H: The CEBPA ChIP-Seq track does not look convincing. Is this region significantly enriched, i.e. called as a peak? Since this is a crucial part of the proposed mechanism, the authors would need to address this experimentally (see point for Fig. 5A-C)

5I: The authors should map the TF binding sites (TEAD and CEBPA) and mutate them using point mutations. This is critical to bolster to support the proposed mechanism of a joint regulation by TCV.

Figure 7:
Is Super-TDU really on target: do you see upregulation of YAP/TAZ target genes after treatment?

Minor comments:
- Header line 149: Hepatocyte-specific...
- Sup. FigS1B: The authors claim upregulation of VGLL4 in patient-derived liver samples in a subset of MAFLD cases. Only 3 out of 3 cases seem to upregulate VGLL4, in the others the expression is similar to controls. Please quantifiy VGLL4 levels
- FigS1E: Statistics in legend is missing.
- FigS2B: Co-staining with hepatocyte marker (e.g. HNF4) would be more convincing to show hepatocyte specific OE
- Fig1I: Scale bar in all pics

Referee #3:

In this manuscript, Chen et al. identify VGLL4 as a regulator of MAFLD progression. Hepatocyte-specific overexpression of VGLL4 induced hepatic steatosis, whereas genetic ablation of VGLL4 in hepatocytes protected mice from high-fat diet-induced metabolic dysfunction. Mechanistically, the authors demonstrate that VGLL4 functions as a transcriptional activator by forming a complex with TEAD4 and CEBPA, thereby promoting PDK4 expression. In contrast, YAP overexpression disrupted the activity of this complex and attenuated steatosis. The findings are novel, mechanistically supported by the presented experimental data, and of broad relevance to hepatology and to the understanding of Hippo pathway signaling in metabolic liver disease.

These findings are novel, mechanistically supported by the presented experimental data, and of broad relevance to hepatology and to the understanding of Hippo pathway signaling in metabolic liver disease. Importantly, the study expands the functional repertoire of VGLL4 beyond its established role as a YAP antagonist and positions it as a context-dependent transcriptional co-activator in hepatocytes. By demonstrating that VGLL4 promotes steatosis through a TEAD4-CEBPA complex that drives PDK4 expression, the work connects Hippo pathway components to metabolic flux control and mitochondrial substrate utilization. This mechanistic link provides a conceptual framework for how transcriptional cofactor dynamics can reprogram hepatocyte metabolism during MAFLD progression.

Major remarks:
1. Human samples: there is no information about the fibrosis stage in the MAFLD patient samples where VGLL4 expression is analyzed. Without fibrosis staging, it's unclear whether increased VGLL4 correlates specifically with steatosis, or instead with fibrosis or inflammation.
2. The use of Mx1-Cre complicates the interpretation of the claim that Vgll4 deletion in the liver inhibits HFD-induced steatosis. Mx1-Cre does not restrict recombination to hepatocytes; it induces deletion in hematopoietic stem cells and their progeny, including Kupffer cells, infiltrating macrophages, and other immune populations that critically regulate hepatic lipid accumulation under high-fat diet conditions. Given the well-established contribution of liver-resident and bone marrow-derived macrophages to inflammatory signaling, insulin sensitivity, and steatosis progression, the observed protection from steatosis could arise from altered immune cell function rather than a hepatocyte-intrinsic effect of Vgll4 loss. Without lineage-restricted deletion, bone marrow transplantation experiments, or hepatocyte-specific Cre controls, it is not possible to conclude that Vgll4 acts cell-autonomously within hepatocytes to regulate lipid accumulation. Therefore, attributing the steatosis phenotype solely to hepatic Vgll4 deletion is premature and requires additional compartment-specific validation. I thus suggest to repeat this experiment with

another Cre line (Alb-CreER) or AAV-Cre.
3. In addition, VGLL4 overexpression starts at 4 weeks of age (Fig. 1B), but this is not clearly mentioned in the text. Why did they start so early? Mice are not fully adult at that age and it would be better to induce VGLL4 once the mice are adult in order to exclde the possiblkility of effects on liver development.
4. It is unexpected that PDK4 alone would be sufficient to induce hepatic steatosis in VGLL4 overexpressing mice. PDK4 does not directly activate canonical lipogenic transcriptional programs such as SREBP1c or ChREBP, nor is it known to suppress key β-oxidation enzymes or impair very-low-density lipoprotein secretion. Its primary function is to modulate mitochondrial carbon flux rather than to drive lipid synthesis or storage. How could PDK4 by itself explain the steatosis effects driven by VGLL4 expression? This requires validation, as additional metabolic or inflammatory perturbations would likely be required to produce this phenotype. Do the authors speculate that PDK4 could induce the expression of lipogenic genes? This should be more explicitely discussed.

Minor remarks:
- Check the gene, mRNA and protein nomenclatures and adapt the text accordingly.
- The authors claim "VGLL4 functions as an enhancer". However, enhancers are genetic motifs, not proteins. The correct nomenclature would be VGLL4 acts as a transcriptional activator instead as a transcriptional repressor.

EMBOR-2026-63711-T _Point-to-point response

We would like to thank the reviewers and the editor for their thoughtful and constructive comments, which have helped us improve the rigor and clarity of our manuscript. In this study, we investigated the role of VGLL4 in the progression of metabolic dysfunction-associated fatty liver disease (MAFLD). Our data show that VGLL4 expression is upregulated in HNF4α-positive hepatocytes from MAFLD patients. Using hepatic *Vgll4* gain- and loss-of-function mouse models, we demonstrate that *Vgll4* overexpression promotes spontaneous hepatic steatosis, insulin resistance, and inflammation under normal chow conditions, whereas *Vgll4* deficiency protects mice from HFD-induced hepatic steatosis and metabolic dysfunction. Mechanistically, VGLL4 forms a transcriptional activation complex with TEAD4 and CEBPA at the *Pdk4* promoter, thereby activating *Pdk4* transcription and increasing PDK4 expression to promote lipid accumulation and metabolic dysfunction. Notably, YAP antagonizes VGLL4 function by competing for TEAD4 binding. Finally, we show that Super-TDU-mediated blockade of the VGLL4–TEAD interaction alleviates *Vgll4*-driven MAFLD phenotypes, highlighting the therapeutic potential of targeting the VGLL4/TEAD4/CEBPA–PDK4 axis.

We have closely followed the detailed comments from the reviewers and performed new experiments. Our new results further support our original conclusion. Following suggestions from all reviewers, we also have substantially revised the text to improve its clarity and to avoid potential confusion for general readers.

Here we summarize major new data included in the revision:

- **Addressed concerns regarding mouse model specificity and developmental effects by adding two virus-mediated in vivo approaches**: AAV2/8-TBG-Cre-mediated *Vgll4* deletion in adult *Vgll4*$^{f/f}$ mice to validate the hepatocyte-targeted role of VGLL4 loss, and adenovirus-mediated *Vgll4* overexpression in adult WT mice to test whether VGLL4 overexpression initiated after development is sufficient to promote hepatic lipid accumulation. - ***Reviewer 2 & 3***.
- **Addressed concerns regarding the direct regulation of *Pdk4* by the VGLL4/TEAD4/CEBPA complex** by adding spike-in-normalized CUT&Tag and motif-mutated luciferase assays. These experiments showed TEAD4/CEBPA occupancy at the *Pdk4* promoter, demonstrated that mutation of their predicted binding motifs impaired promoter activation, and revealed that VGLL4 loss reduced their binding at the *Pdk4* locus. - ***Reviewer 2***.
- **Clarified the functional role of PDK4 downstream of VGLL4** by expanding the Discussion on how PDK4-mediated metabolic reprogramming may contribute to VGLL4-driven hepatic steatosis, while acknowledging that PDK4 may act together with broader VGLL4-regulated metabolic pathways. - ***Reviewer 1 & 3***.
- **Clarified the context-dependent interplay between VGLL4 and YAP at the *Pdk4* locus** by revising the mechanistic rationale and Discussion. These revisions better explain how VGLL4 functions as a TEAD4/C/EBPα-associated transcriptional activator in hepatocytes, whereas YAP antagonizes VGLL4-dependent *Pdk4* activation through competition for TEAD4 binding. - ***Reviewer 2***.

Below are our detailed point-to-point responses for each reviewer.

Reviewer 1:

This manuscript by Yixing Chen and colleagues represent a very solid amount of work that highlights the effect of VGLL4 on lipid metabolism in hepatocytes with possible relevance to MASLD. The quantity of work that has been done is impressive, including overexpression and knock out studies in vivo and in cell systems. Overall, in my opinion, this is a super solid work and the authors have to be congratulated for the amount of high quality work.

Response: We sincerely thank the reviewer for the very positive comments and encouraging evaluation of our work. We are also grateful for the constructive suggestions, which are highly valuable for improving the current manuscript and will help guide our future studies on this topic.

1. The authors have acknowledged that they have not identified how VGLL4 becomes overexpressed in the context of MASLD which would have been interesting to know.

   Response: We appreciate the reviewer's insightful comment. We agree that the upstream mechanisms leading to VGLL4 induction in MAFLD remain an important question. The heterogeneous VGLL4 upregulation observed in human MASLD samples suggests that VGLL4 expression may be influenced by disease stage and/or patient-specific factors. Future studies using larger patient cohorts and experimental validation will be needed to identify the upstream regulators of VGLL4 in fatty liver disease.

2. In my opinion, there is one other major limitation to this study. The authors show that VGLL4 influences the expression of many genes including lipogenic genes. But they claim that VGLL4/Tead/CEBPA modulates steatosis by regulating the transcription of PDK4. I am not sure to understand why can they claim that VGLL4/Tead/CEBPA specifically regulate PDK4 and not a broader range of genes involved in metabolic homeostasis.

   Response: Regarding the role of PDK4, we agree that PDK4 should not be considered the sole downstream mediator of VGLL4-dependent metabolic regulation, as VGLL4 modulation led to broader transcriptomic changes involving multiple lipid metabolism-related genes. However, our study specifically prioritized PDK4 because we sought to identify a key hub downstream effector of the complex that makes a major contribution to the steatotic phenotype. Given the complexity of hepatic lipid metabolic regulation, focusing on PDK4 as a functionally relevant target allowed us to dissect a clearer mechanistic path from VGLL4 complex formation to direct promoter regulation and then to the in vivo steatotic phenotype. This focused approach also provides a more defined mechanistic basis for exploring potential therapeutic strategies targeting this pathway in MAFLD. PDK4 was selected for this focused analysis because it showed one of the most pronounced changes among lipid metabolism-related genes upon VGLL4 modulation. (Fig. 5A and Fig. EV3A, B). Moreover, VGLL4, TEAD4, and CEBPA directly occupied and activated the *Pdk4* promoter (Fig. 5C, I and J), and hepatocyte-targeted PDK4 knockdown substantially alleviated VGLL4-induced

hepatic steatosis (Fig. 6), supporting PDK4 as an important downstream effector while still allowing for contributions from additional VGLL4-regulated metabolic pathways.

3. If PDK4 is the responsible for the effect on steatosis, how does PDK4 modulation influence the whole transcriptome including lipogenic genes?

Response: Regarding how PDK4 modulation may influence lipogenic gene expression and hepatic steatosis, we agree that PDK4 is unlikely to function as a direct transcriptional regulator of the lipid metabolic transcriptome. Instead, PDK4 may influence hepatic lipogenic gene expression through multiple metabolic and signaling pathways, with emerging evidence also suggesting non-canonical functions beyond PDC regulation. For example, PDK4 has been reported to exacerbate steatosis in obesity and MASH-related models, whereas its genetic deficiency or pharmacological inhibition attenuates hepatic lipid accumulation, at least in part through enhanced AMPK signaling, reduced ChREBP nuclear translocation, and decreased expression of lipogenic genes such as *Acc1*, *Fasn*, and *Scd1*.[1,2] PDK4 may also regulate hepatic lipid metabolism through the canonical modulation of pyruvate dehydrogenase complex activity.[3,4] In addition, PDK4 has been implicated in hepatocyte lipid accumulation through kinase-dependent regulation of mitochondria-associated ER membranes, independently of the canonical PDC pathway.[5] Consistent with these previous findings, our data further showed that PDK4 overexpression promoted lipid droplet accumulation and lipid uptake in hepatocytes under PA/OA treatment (Fig. 5D, E). Together, these findings suggest that PDK4 may contribute to hepatic lipid accumulation and associated lipogenic changes through multiple metabolic routes. Nevertheless, the additional mechanisms by which PDK4 promotes hepatic lipid accumulation remain to be further investigated.

We have further incorporated this point into the revised Discussion section.

4. A minor remark, given that lipid accumulation is a major observation in this study, a more detailed analysis of changes in tissue lipid and cell lipid composition would have been interesting.

Response: We thank the reviewer for this insightful suggestion. We fully agree that a more detailed lipidomic analysis would provide valuable information for understanding how VGLL4 reshapes hepatic lipid metabolism. A systematic comparison of the lipid composition of *Vgll4*-overexpressing livers with that of classical MAFLD models, such as HFD-fed mice, as well as human MAFLD samples, would be highly informative. Such analysis may help determine whether the *Vgll4*-overexpressing mouse model recapitulates specific lipid metabolic features of MAFLD and further support its use for studying early steatotic remodeling or therapeutic screening. We appreciate the reviewer's constructive suggestion.

5. Finally, the authors used the term MAFLD and NASH. I am not sure but it might be better to use MASLD and MASH? Please check what would the most suitable nomenclature.

Response: We thank the reviewer for this careful and timely comment. Accordingly, we have checked the manuscript and revised the terminology throughout the text. We have corrected NASH to MASH in the relevant sentences. The following sentences have been revised:

- "Lipotoxicity triggered by excessive lipid accumulation can cause endoplasmic reticulum stress and mitochondrial dysfunction, leading to ~~NASH~~ **MASH**, which is characterized by inflammation and hepatocyte ballooning degeneration."

- "Consistent with these mechanisms, PDK4 exacerbates steatosis in obesity and ~~NASH~~ **MASH** models,"

Reviewer 2:

In this manuscript Chen et al. identify that VGLL4 is induced in MAFLD. Using mouse models they go on to show that VGLL4 oe in the liver induces hepatic steatosis and other phenotypes (glucose intolerance, insulin resistance), and that VGLL4 KO makes the mice resistant to these changes after HFD. The authors propose that a trimeric complex of TEAD/CEBPA/VGLL4 (TCV) directly upregulates PDK4 expression which they propose to be the critical target gene for the observed phenotypes. In general, the manuscript addresses a topic of high relevance. Also the use of Sper-TDU is very interesting and shows some translational value of their findings. The authors mostly use appropriate tools to address their research question. However, several weaknesses (particularly with regard to the proposed mechanism), which are outlined in detail below, dampen this reviewer's enthusiasm. This reviewer is not yet convinced regarding the proposed linear pathway of the putative TEAD/CEBPA/VGLL4 (TCV) complex directly regulating PDK4. The data presented would also be consistent with a parallel model: TCV regulates metabolism via genes XYZ and this can be normalized by downregulating the master regulator PDK4. This part would need to be substantially bolstered by additional experiments, e.g. ChIP-Seq/CUT&RUN (specified below).

Response: Thank the reviewer for the valuable and constructive comments from multiple perspectives, which have helped us improve the quality of our manuscript substantially. We sincerely hope that the revised version has adequately addressed the reviewer's concerns and better meets the reviewer's expectations.

1. Statistics:
- The authors should rather use a Welch than a Student's t-test
- For repeated measurements (e.g. insulin resistance) a two-way ANOVA is more appropriate

Response: We thank the reviewer for pointing out these important statistical issues. Following the reviewer's suggestions, two-group comparisons were reanalyzed using Welch's t-test, comparisons involving more than two groups were analyzed using one-way ANOVA, and repeated-measurement experiments, including GTT, ITT, and longitudinal body weight measurements, were reanalyzed using two-way ANOVA.

The statistical annotations, figure panels, and related text have been revised accordingly. These reanalyses did not alter the main conclusions of the study.

2. Fig. 1: How long have the mice been on doxycycline food? What is the control group?

Response: We apologize that the genetic design of this mouse model was not sufficiently clear in the original manuscript. The *LAP-tTA, TetO-Vgll4* mice were generated by crossing *LAP-tTA* mice with *TetO-Vgll4* transgenic mice. This model uses a Tet-off system, in which VGLL4-FLAG expression is activated in the absence of doxycycline and suppressed upon doxycycline administration. Therefore, the mice shown in Fig. 1 were not fed doxycycline-containing food; VGLL4-FLAG was overexpressed under doxycycline-free conditions. The control group consisted of littermate *TetO-Vgll4* single-transgenic mice without the *LAP-tTA*

driver, which carry the VGLL4-FLAG transgene but could not activate its expression regardless of doxycycline administration.

We have revised the Methods section to clarify the Tet-off strategy, doxycycline-free condition, and control genotype.

3. Fig. S1B/C: The upregulation seems rather minor (only 1 shows strong, 2 show weak upregulation - 3 none). The authors should do co-stainings with HNF4a to demonstrate that VGLL4 upregulation occurs in hepatocytes. The presented data is not convincing.

Response: We acknowledge that the extent of VGLL4 upregulation varies among individual samples, which is likely related to the heterogeneity of human liver specimens. Following the reviewer's suggestion, we performed co-immunofluorescence staining for VGLL4 and HNF4α (Fig. EV1D). The new data showed that increased VGLL4 staining was predominantly detected in HNF4α-positive hepatocytes in MAFLD tissues compared with control liver tissues. These results support that VGLL4 upregulation occurs, at least in part, in hepatocytes.

EV1D

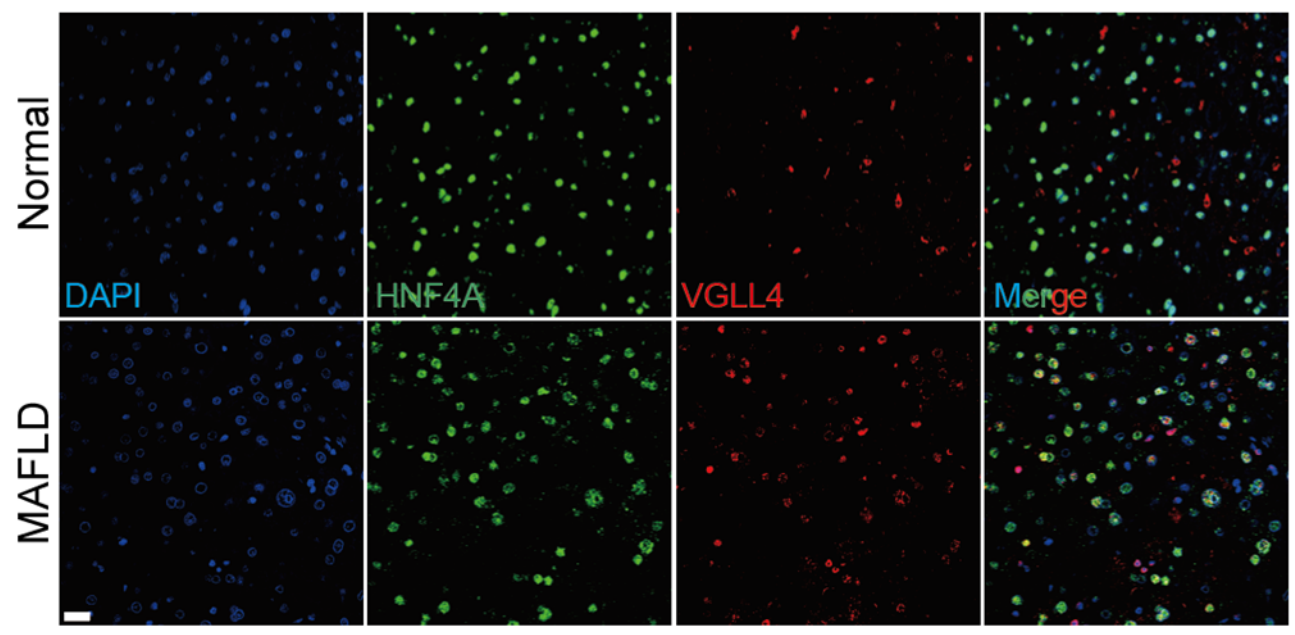

(EV1D) Representative immunofluorescence co-staining for VGLL4 and the hepatocyte marker HNF4α in liver tissues from control and MAFLD patients (n=3). Scale bar, 20 μm.

We have revised the corresponding text and figure legend accordingly.

4. Fig. 2: Are the changes, e.g. in 2C, related to the steatosis phenotype or to VGLL4 oe?. The authors should analyze these proteins after VGLL4 induction when no steatosis has developed yet.

S6H **S6I**

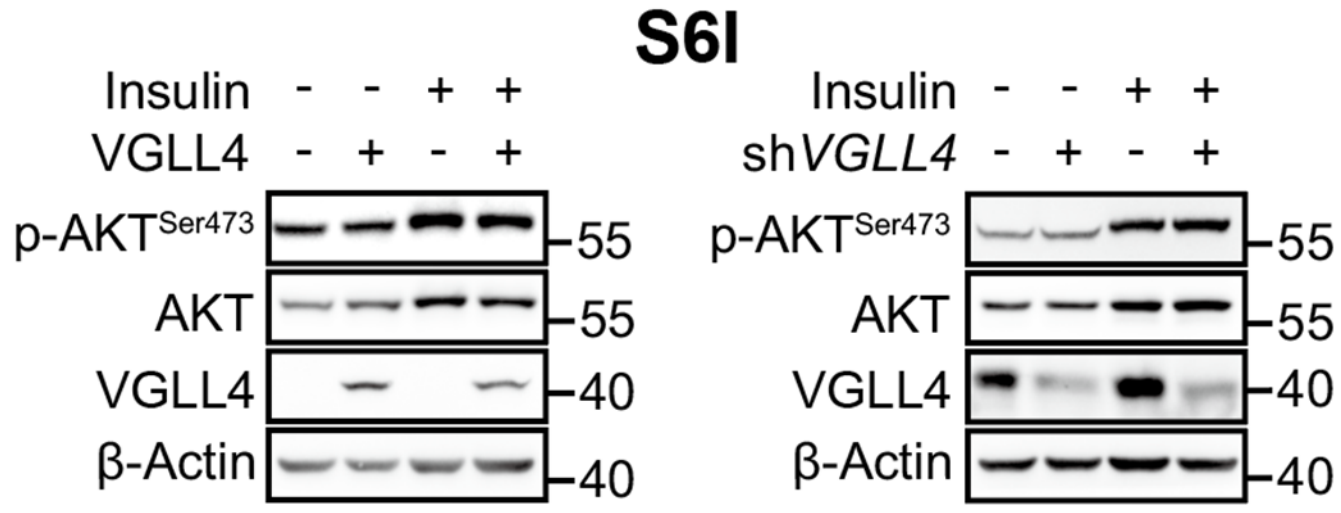

(S6H, I) Western blot detection of p-AKT^{Ser473}, AKT, and VGLL4 in L02 cells with *VGLL4* overexpression or knockdown treated with or without insulin.

Response: We thank the reviewer for this insightful comment. Regarding the causal relationship between *Vgll4* overexpression, steatosis, and insulin signaling, relevant *in vitro* data had been included in the original manuscript, but this point was not sufficiently discussed. In L02 cells, *VGLL4* overexpression promoted lipid accumulation, whereas VGLL4 knockdown reduced lipid accumulation (Appendix Fig. S6). However, VGLL4

modulation did not further alter AKT phosphorylation, a key readout of insulin signaling (Appendix Fig. S6H, I). These data suggest that VGLL4 itself does not directly suppress AKT phosphorylation in a simple hepatocyte cell-autonomous setting, even though it affects lipid accumulation. Therefore, the reduced phosphorylation of IRS1, AKT, and FOXO1 observed in *Vgll4*-overexpressing mouse livers likely reflects impaired hepatic insulin signaling associated with the chronic lipotoxic environment, rather than an immediate direct effect of *Vgll4* overexpression on insulin signaling proteins.

To make this point clearer, we have revised the Discussion and explicitly explained the relationship between *Vgll4*-induced lipid accumulation and insulin resistance.

5. Fig. 3: VGLL4 fl/fl mice as control without the transgene (Cre) is not an ideal control. Better control animals would fl/+; cre/+ or +/+; cre/+, Using Mx1-Cre for "liver-specific" KOs is very unusual and not very clean. Mx1-Cre is also highly active in hematopoeitic cells which could confound the analyses here. At least, the authors should discuss this shortcoming and make the reader aware of this. Ideally, rather use Alb-CreERT or Adeno-Cre.

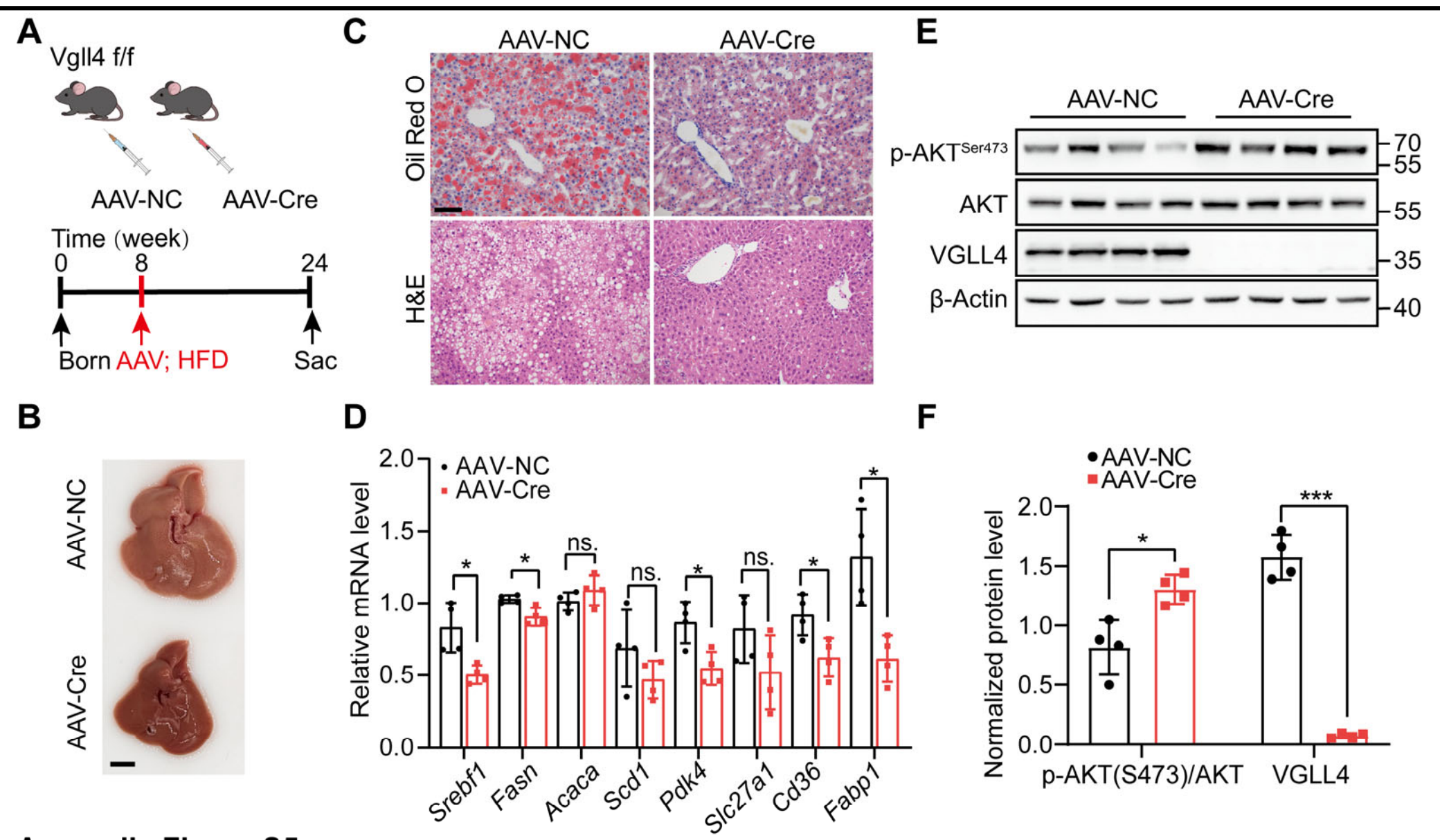

Appendix Figure S5

Appendix Figure S5 AAV2/8-TBG-Cre-mediated *Vgll4* deletion protects adult mice from HFD-induced hepatic steatosis and impaired insulin signaling

(A) Schematic diagram of animal experiments. *Vgll4*$^{f/f}$ mice were injected with AAV2/8-TBG-Cre (AAV-Cre) or AAV negative control vector (AAV-NC) through the tail vein, and the metabolic phenotype was assessed. (B) Representative images of the livers (n=3). Scale bar, 5 mm. (C) Representative images of Oil Red O (upper panel) and H&E (lower panel) stained liver sections (n=3). Scale bar, 100 μm. (D) The mRNA levels of genes involved in fatty acid synthesis and uptake from the indicated groups, with expression levels normalized to those of β-actin (n=4). (E, F) Mice were intraperitoneally injected with insulin following 4 hours fasting. Activation of the insulin signaling pathway in the livers of mice from the indicated groups was detected by Western blotting (E), the protein levels of insulin signaling-related molecules were normalized to β-actin (F) (n=4). Data were presented as mean ± SD. For (D) and (F), significance was determined by Welch t-test. *p < 0.05, ***p < 0.001, ns. indicates no significant difference.

Response: We agree that Mx1-Cre is not an ideal hepatocyte-specific Cre system because of its activity in hematopoietic cells. Given this limitation, we had initiated an independent validation experiment during the course of this study using AAV2/8-TBG-Cre to achieve hepatocyte-targeted *Vgll4* deletion in adult *Vgll4$^{f/f}$* mice (Appendix Fig. S5A). After HFD feeding, hepatocyte-targeted *Vgll4$^{f/f}$* deletion by AAV2/8-TBG-Cre produced results consistent with those observed in the Mx1-Cre model, including reduced hepatic lipid accumulation and improved insulin signaling-related phenotypes (Appendix Fig. S5B–F). These data further support a hepatocyte-associated role of VGLL4 in HFD-induced hepatic steatosis and metabolic dysfunction, and reduce the concern that the phenotype observed in the Mx1-Cre model was caused by Cre activity in hematopoietic cells.

We have now included these validation data in the revised manuscript and modified the related text accordingly.

6. Fig. 4:

6.1 4A: Please provide a quantification with proper statistical test (one-way ANOVA). How acute are the gene expression changes. Please use an inducible system to analyze this. If oe persists over weeks, the effects could be due to selection processes as compared to gene regulation.

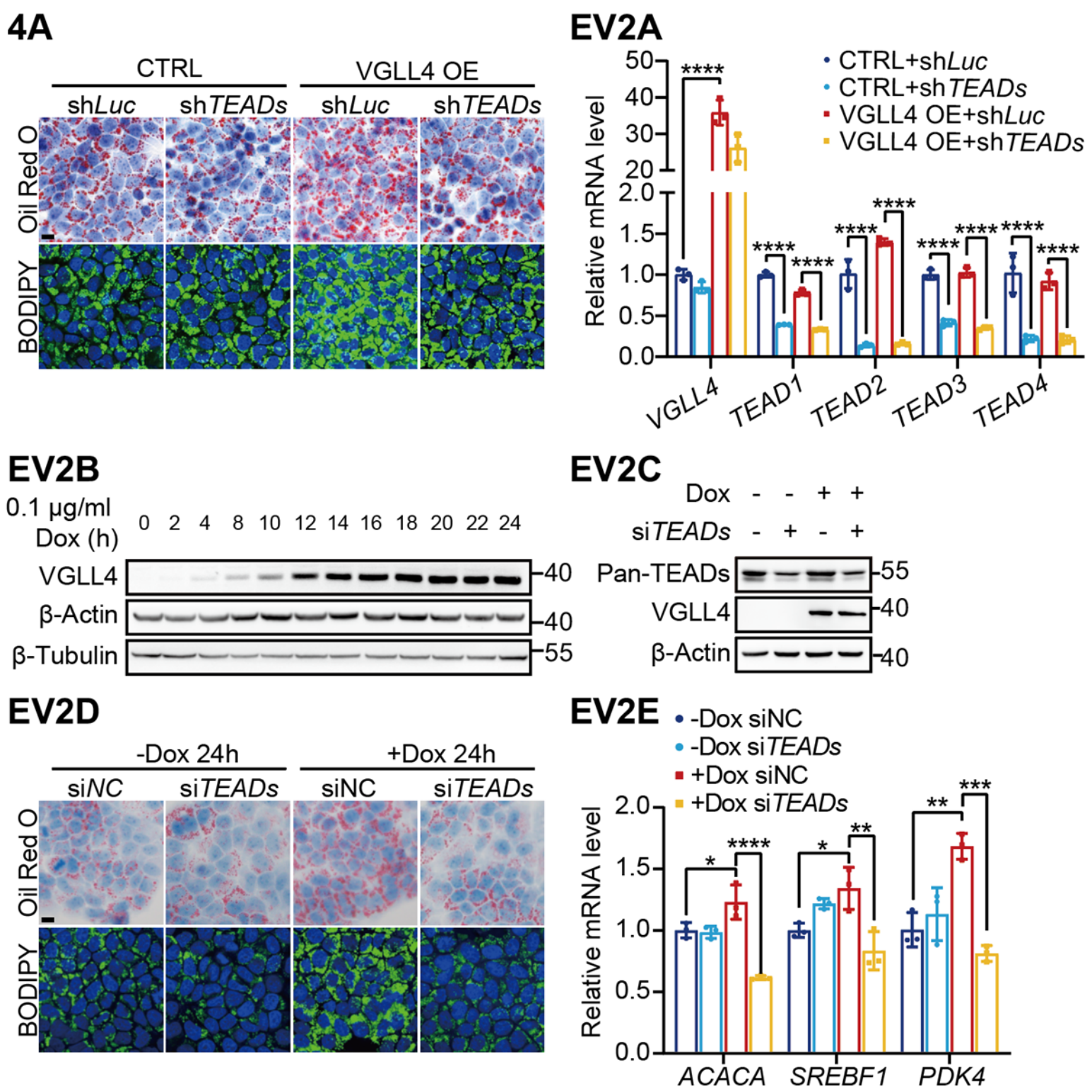

(4A) The representative images of Oil Red O-stained (the first panel) and BODIPY 558/568 C12-stained (the second panels) L02 cells from the indicated groups (scale bar, 20 μm). (EV2A) RT-qPCR analysis of VGLL4 and TEAD1–4 mRNA levels in control or *VGLL4*-overexpressing L02 cells transduced with sh*Luc* or sh*TEADs*, with expression levels normalized to those of β-actin (n=3). (EV2B) Western blot analysis showing Dox-induced VGLL4 expression in the inducible *VGLL4*-overexpressing L02 cell line. Cells were treated with 0.1 μg/mL Dox for the indicated times. (EV2C) Western blot validation of siRNA-mediated *TEADs* knockdown

and Dox-induced VGLL4 expression in inducible *VGLL4*-overexpressing L02 cells treated with or without Dox. (EV2D) Representative images of Oil Red O-stained cells and BODIPY 558/568 C12-stained cells from the indicated groups after PA/OA treatment in the presence or absence of Dox (scale bar, 20 μm). (EV2E) The relative mRNA levels of genes related to fatty acid metabolism and regulation in L02 cells from the indicated groups, with expression levels normalized to those of β-actin (n=3). Data were presented as mean ± SD. For (EV2A), (EV2E), significance was determined using the one-way ANOVA. *p < 0.05, **p < 0.01, ***p < 0.001, ****p < 0.0001 as indicated.

Response: We thank the reviewer for this suggestion. First, we have quantified the knockdown efficiency of *TEAD* family members and the overexpression efficiency of *VGLL4* in the experiment shown in Fig. 4A (Fig. EV2A). The data were analyzed using one-way ANOVA, as suggested by the reviewer. Meanwhile, we also generated a Tet-on *VGLL4* stable L02 cell line. Upon doxycycline treatment, VGLL4 expression increased in a time-dependent manner and reached a peak at approximately 16 h after induction (Fig. EV2B). In this inducible system, doxycycline-induced *VGLL4* expression promoted lipid droplet accumulation under PA/OA treatment, whereas knockdown of *TEAD* family members attenuated this phenotype (Fig. EV2C, D). Consistently, at the molecular level, *TEADs* knockdown also reduced *VGLL4*-induced changes in lipid metabolism-related genes (Fig. EV2E). These results are consistent with our original findings obtained using the constitutive VGLL4 overexpression system and indicate that the lipid accumulation phenotype induced by VGLL4 depends on TEADs.

We have added these data to the revised manuscript and modified the related text accordingly.

6.2 4H: Please provide a schematic showing which domains are deleted in CEBPA.

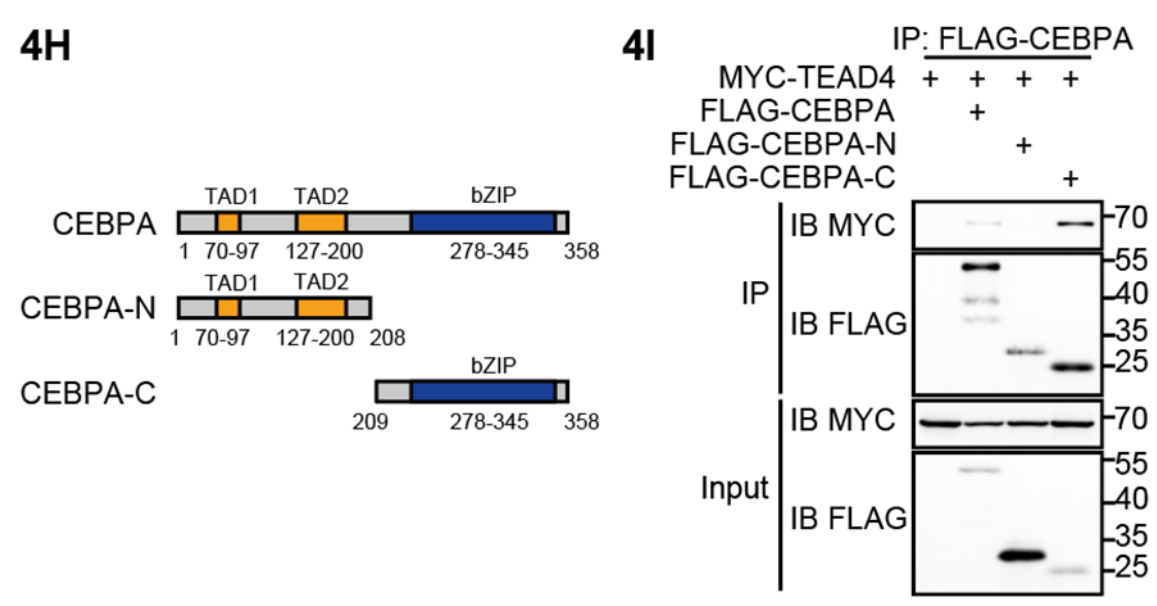

(4H) Schematic representation of full-length CEBPA and its truncation mutants. CEBPA-N (aa 1-208) retains the transactivation domains (TAD1 and TAD2), whereas CEBPA-C (aa 209-358) contains the bZIP DNA-binding domain. (4I) Co-immunoprecipitation assays were conducted to investigate the interaction between TEAD4 and full-length CEBPA or its N-terminal/ C-terminal fragment.

Response: We thank the reviewer for this helpful suggestion, which improved the readability of our mechanistic presentation. We have added a schematic to Fig. 4H to show the domain structure of full-length CEBPA and the deletion constructs used in this study. The revised figure indicates that full-length CEBPA contains two N-terminal transactivation domains (TAD1 and TAD2) and a C-terminal bZIP domain (Fig. 4H), and that TEAD4

predominantly interacts with the C-terminal CEBPA fragment containing the bZIP domain (Fig. 4I).

The figure legend and related text have been revised accordingly.

6.3 4I: I find the argument regarding the DNA dependency hard to follow. The authors claim that TEAD and CEBPA bind to joint targets. By using DNAse I they simply demonstrate that the PPI is independent of DNA (which is crucial). However, if CEPBA binds to the TEAD DNA binding domain, it could also interfere with TEAD's ability to bind to DNA?

Response: We thank the reviewer for this important clarification. We agree that the interpretation of the DNase I experiment was not sufficiently clear in the original manuscript. The DNase I digestion experiment only supports that the TEAD4–CEBPA interaction is not mediated by DNA fragments, but does not prove simultaneous binding of the two proteins to the same DNA element. The previous statement regarding “joint targets” was based on the proximity of TEAD4 and CEBPA peaks in existing ChIP-seq datasets (Fig. 5H) and has now been revised to avoid overinterpretation. We now describe TEAD4 and CEBPA as potentially binding to proximal regions within the *Pdk4* promoter.

6.4 The authors should also analzye whether VGLL4 oe/KD has an acute impact on CEBPA protein expression in the L02 cell line.

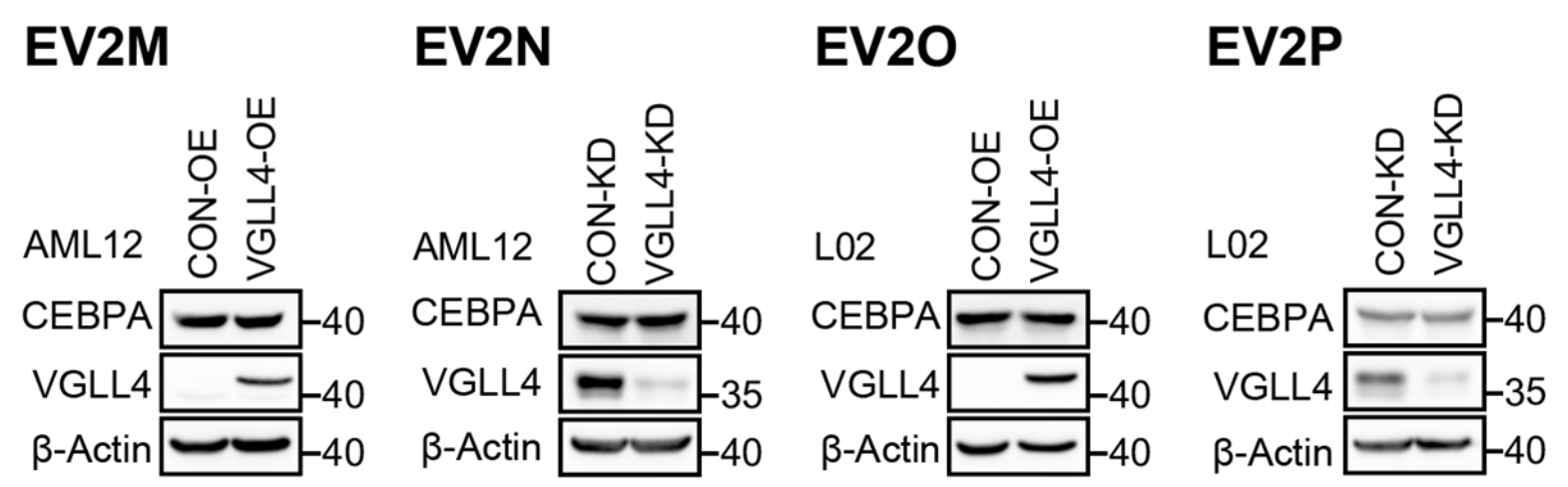

(EV2M–P) Western blot analysis of CEBPA protein levels in AML12 and L02 cells after *VGLL4/Vgll4* overexpression or knockdown.

Response: Thank the review for this suggestion. We examined CEBPA protein expression in mouse AML12 hepatocytes and human L02 hepatocytes after *Vgll4/VGLL4* overexpression or knockdown. VGLL4 modulation did not noticeably alter CEBPA protein levels, suggesting that VGLL4 does not promote PDK4 expression by increasing CEBPA abundance.

We have added these analyses to the revised manuscript and revised the related text accordingly.

7. Fig. 5:

7.1 5A-C: If the model is that CEBPA and TEAD bind to joint target genes (e.g.) and this interaction is stabilized by VGLL4. This needs to be shown in a genome-wide ChIP-Seq/CUT&RUN experiment using shCtrl vs. shVGLL4. Please use spike-in controls for normalization here.

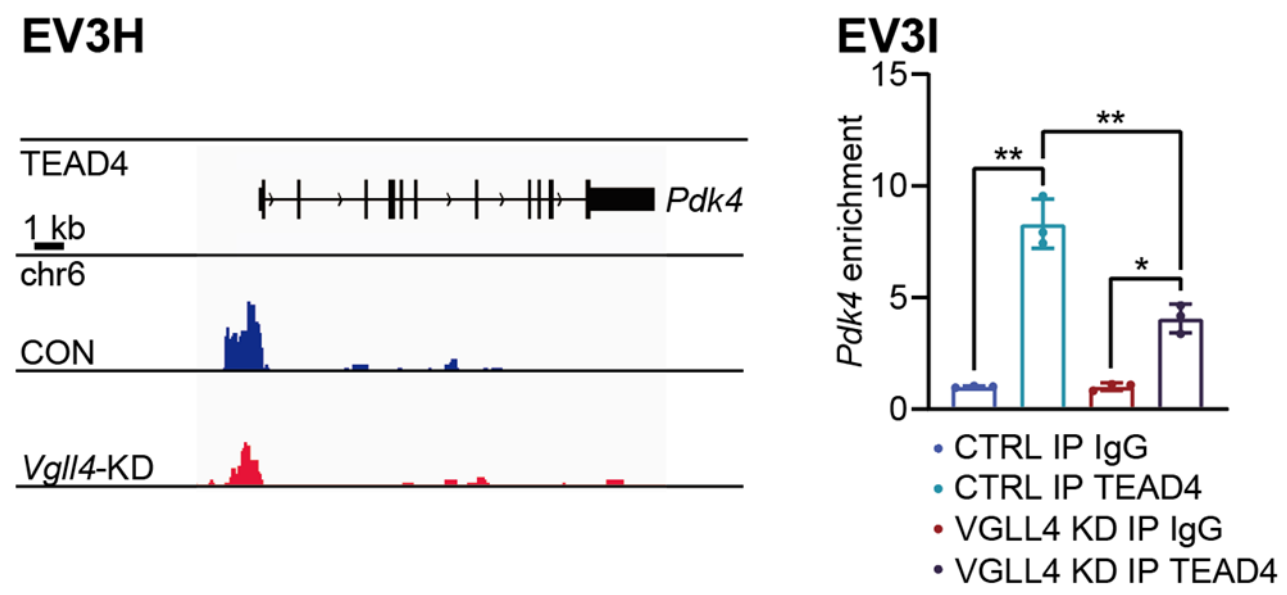

(EV3H) TEAD4 CUT&TAG tracks at the *Pdk4* locus in control and *Vgll4*-knockdown AML12 cells. (EV3I) ChIP-qPCR validation of TEAD4 enrichment at the *Pdk4* promoter after *Vgll4* knockdown AML12 cells (n=3). Data were presented as mean ± SD. For (EV3I), significance was determined using the two-way ANOVA. *p < 0.05, **p < 0.01 as indicated.

Response: We thank the reviewer for this important suggestion. We performed spike-in-normalized TEAD4 CUT&TAG in control and *Vgll4*-knockdown AML12 cells. Due to antibody compatibility limitations, reliable CEBPA CUT&TAG data could not be obtained. TEAD4 CUT&TAG showed reduced TEAD4 occupancy at the *Pdk4* promoter region after *Vgll4* knockdown (Fig. EV3H), which was further confirmed by TEAD4 ChIP-qPCR (Fig. EV3I). These results support that VGLL4 contributes to maintaining TEAD4 occupancy at the *Pdk4* regulatory region.

We have added these data and revised the corresponding text accordingly.

7.2 5F: The authors need to show the protein expression of PDK4. Does shPDK4 bring dwon PDK4 expression to control conditions (Ctrl / sh Luc).

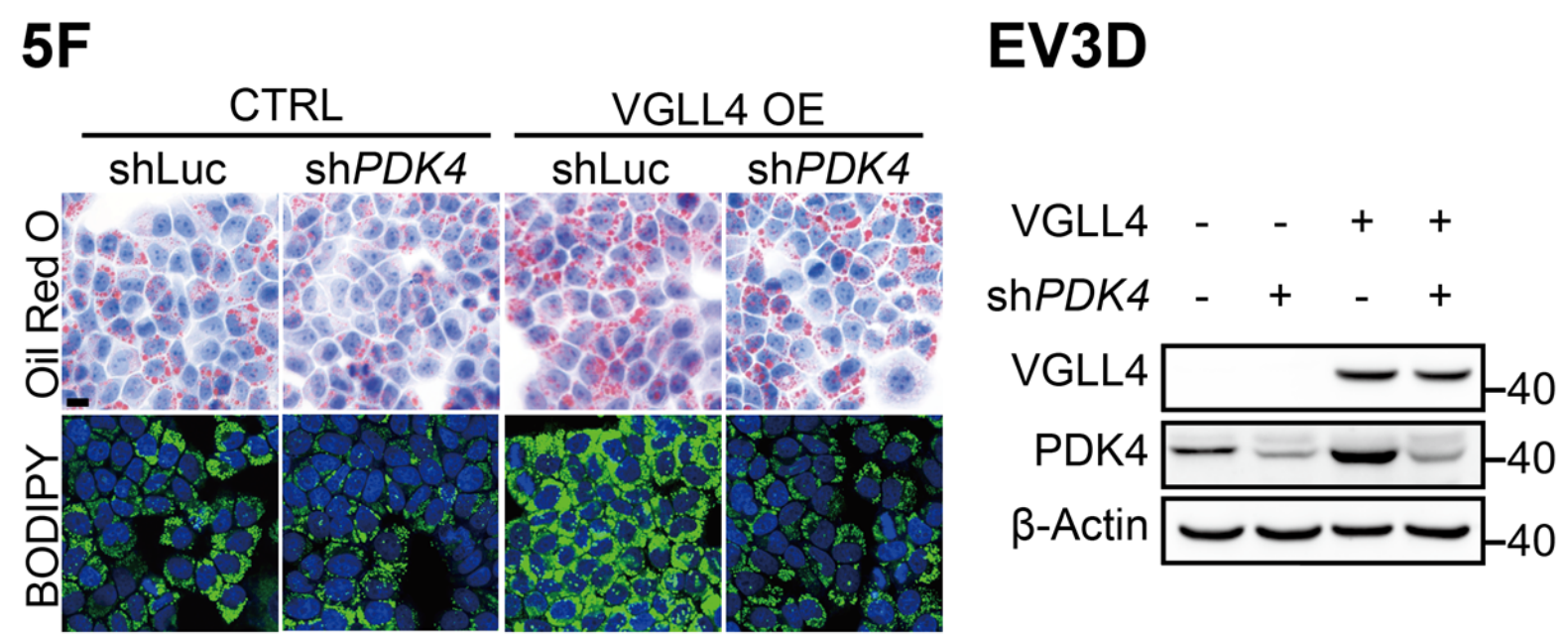

(5F) The representative images of Oil Red O-stained (the first panel) and BODIPY 558/568 C12-stained (the second panels) L02 cells from the indicated groups (scale bar, 20 μm). (EV3D) The validation of the constructed L02 stable cell line.

Response: We thank the reviewer for this suggestion. We have added Western blot analysis of PDK4 protein expression in the Figure. EV3D. VGLL4 overexpression increased PDK4 protein levels, whereas shPDK4 markedly reduced PDK4 expression to a level lower than that in control cells.

The figure legend and related text have been revised accordingly.

7.3 Based on their model, the PDK4 expression should go up after VGLL4 oe. This per se, is against the current model of how VGLL4 acts in conjunction with TEAD-YAP/TAZ: it normally acts as a repressor (this would need be addressed by the authors).

Response: We appreciate the reviewer for raising this conceptual point. We would like to clarify that, in the classical YAP/TAZ–TEAD model, VGLL4 does not act in conjunction with YAP/TAZ. Instead, VGLL4 competes with YAP/TAZ for TEAD binding, thereby disrupting the formation and function of the YAP/TAZ–TEAD transcriptional complex.[6,7] In this sense, VGLL4 and YAP/TAZ act antagonistically in their interaction with TEADs, rather than VGLL4 simply functioning as a general transcriptional repressor. During tumorigenesis, YAP/TAZ can form transcriptional complexes with TEADs to promote the expression of pro-oncogenic target genes, while VGLL4 antagonizes this process by competitively binding to TEADs, thereby giving rise to an apparent <u>repressive</u> effect.[6–8] However, during myogenic differentiation, VGLL4 can compete with YAP/TAZ for TEAD4 binding and <u>promote</u> MyoG transcription as a transcriptional co-activator, thereby supporting myogenic differentiation.[9] In addition to antagonizing the YAP/TAZ–TEAD4 transcriptional complex, VGLL4 has also been reported to directly function as a TEAD-associated transcriptional cofactor with activating effects.[10–12] These examples highlight that the functional relationship between YAP/TAZ and VGLL4 at TEADs is primarily competitive, rather than a fixed pattern in which one cofactor always activates and the other always represses transcription.

In the present study, our data support a metabolic context in which VGLL4 cooperates with TEAD4 and CEBPA to activate *Pdk4* transcription. YAP antagonizes VGLL4-dependent *Pdk4* activation by competing for TEAD4 binding. Thus, the induction of *Pdk4* by *Vgll4* overexpression is consistent with the established competitive relationship between VGLL4 and YAP at TEADs.

We have revised the Discussion to clarify this point and to avoid implying that VGLL4 universally functions as a transcriptional repressor.

7.4 Furthermore, there is only very little - and no strong evidence - that YAP can act as a repressor (related to Fg. S7)

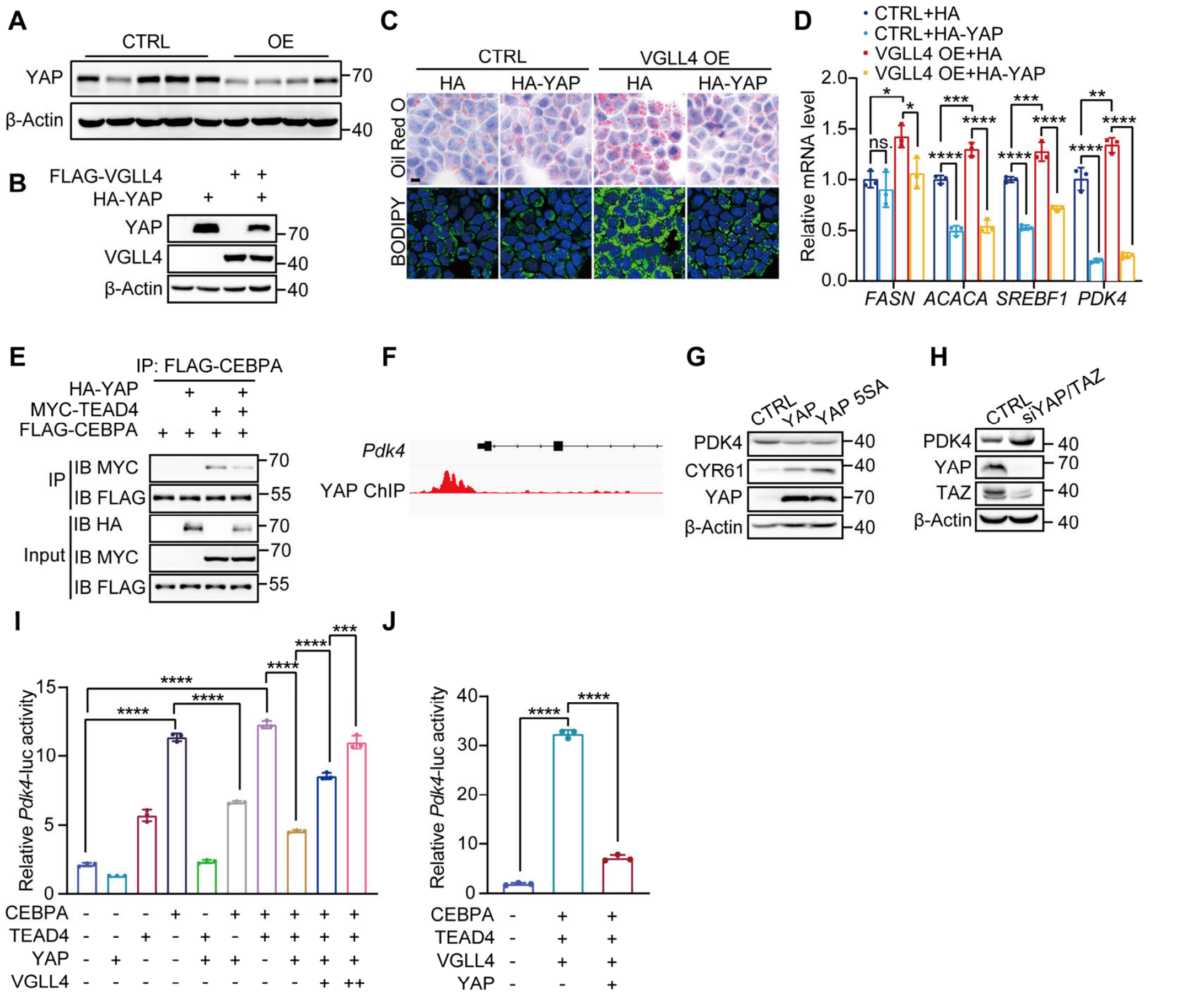

Figure EV4

Figure EV4 The regulatory effect of VGLL4/TEAD/CEBPA on *Pdk4* is antagonized by YAP.

(A) Western blot detection of YAP in CTRL and VGLL4-OE mice (n=4–5). (B) The validation of the constructed L02 stable cell line. (C) The representative images of Oil Red O-stained (the first panel) and BODIPY 558/568 C12-stained (the second panels) L02 cells from the indicated groups (scale bar, 20 μm). (D) The relative mRNA levels of genes related to fatty acid metabolism and regulation in L02 cells from the indicated groups, with expression levels normalized to those of β-actin (n=3). (E) Co-IP assay to assess the impact of YAP on the interaction between CEBPA and TEAD4 in HEK293T cells. (F) ChIP-seq data showing binding peak of YAP (GSM3019324) on the promoter of *Pdk4*. (G, H) Western blot analysis showed the variations of PDK4 and known YAP target in YAP/YAP-5SA-overexpressing and YAP/TAZ-knockdown L02 cells. (I, J) Luciferase reporter assay was performed to analyze the transcriptional activity of the *Pdk4* promoter in L02 cells co-transfected with CEBPA, TEAD4, YAP and VGLL4 (n=3). Data were presented as mean ± SD. For (D), (I, J), significance was determined using the one-way ANOVA. *p < 0.05, ***p < 0.001, ****p < 0.0001, ns. indicates no significant difference.

Response: We agree that YAP is generally recognized as a transcriptional co-activator in many biological contexts, and we did not intend to suggest that YAP broadly functions as a general transcriptional repressor. In our study, YAP exerted an antagonistic effect on VGLL4-dependent PDK4 regulation. Specifically, YAP overexpression reduced PDK4 protein levels, whereas YAP/TAZ knockdown increased PDK4 protein expression (Fig.

EV4G, H). Moreover, in *VGLL4*-overexpressing cells, YAP also attenuated lipid accumulation and reduced the expression of PDK4 and lipid metabolism-related genes which were induced by *VGLL4*-overexpressing (Fig. EV4B, D). Furthermore, in the *Pdk4* promoter luciferase assay, YAP suppressed the promoter activity induced by the CEBPA/TEAD4/VGLL4 complex (Fig. EV4I, J). These data support a model in which YAP inhibits VGLL4-dependent *Pdk4* activation through antagonizing the VGLL4–TEAD4 complex.

We have revised the related text and reorganized Figure EV4 to clarify this point.

7.5 5H: The CEBPA ChIP-Seq track does not look convincing. Is this region significantly enriched, i.e. called as a peak? Since this is a crucial part of the proposed mechanism, the authors would need to address this experimentally (see point for Fig. 5A-C)

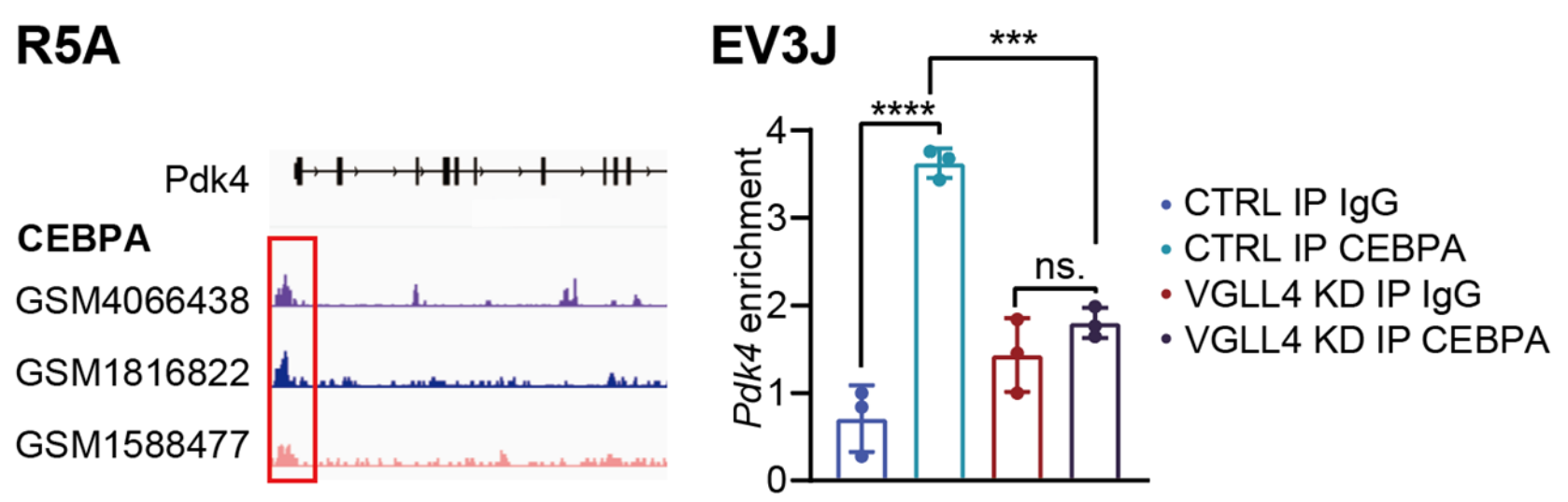

(R5A) ChIP-seq data showing binding peak of CEBPA on the promoter of *Pdk4*. (EV3J) ChIP-qPCR validation of CEBPA enrichment at the *Pdk4* promoter after *Vgll4* knockdown (n=3). Data were presented as mean ± SD. For (EV3J), significance was determined using the two-way ANOVA. ***$p < 0.001$, ****$p < 0.0001$ as indicated.

Response: We thank the reviewer for this important comment. We re-examined multiple publicly available CEBPA ChIP-seq datasets (GSM4066438, GSM1816822 and GSM1588477) and found consistent CEBPA enrichment at the *Pdk4* promoter region (Fig. R5A). Although we attempted CEBPA CUT&Tag, reliable signals could not be obtained due to antibody compatibility limitations. We therefore performed CEBPA ChIP-qPCR, which confirmed CEBPA enrichment at the *Pdk4* promoter and showed that this enrichment was reduced after *Vgll4* knockdown (Fig. EV3J).

These data support CEBPA occupancy at the *Pdk4* promoter and suggest that VGLL4 contributes to maintaining CEBPA enrichment at this locus.

7.6 5I: The authors should map the TF binding sites (TEAD and CEBPA) and mutate them using point mutations. This is critical to bolster to support the proposed mechanism of a joint regulation by TCV.

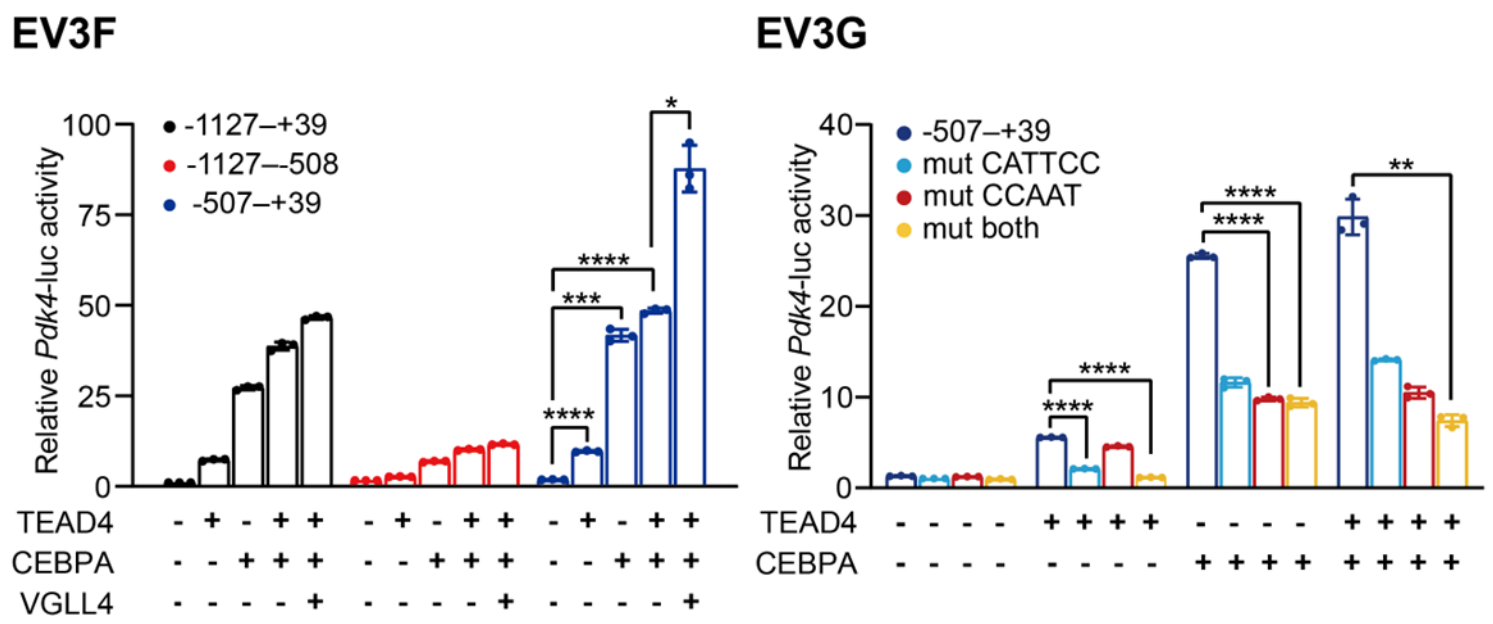

(EV3F) Luciferase reporter assays using serially truncated *Pdk4* promoter constructs in cells co-transfected with the indicated combinations of TEAD4, CEBPA, and VGLL4 (n=3). (EV3G) Luciferase reporter assays using the −507 to +39 *Pdk4* promoter reporter carrying point mutations in the predicted TEAD4-binding motif CATTCC, the predicted CEBPA-binding motif CCAAT, or both motifs (n=3). Data were presented as mean ± SD. For (EV3F, G), significance was determined using the two-way ANOVA. *p < 0.05, ***p < 0.001, ****p< 0.0001 as indicated.

Response: To address this point, we first generated a series of *Pdk4* promoter truncation luciferase reporters. These experiments narrowed the major VGLL4/TEAD4/CEBPA-responsive region to the -507 to +39 region of the *Pdk4* promoter (Fig. EV3F). We then used the JASPAR database to predict potential TEAD4- and CEBPA-binding motifs within this region. Based on this analysis, we introduced point mutations (mutated to adenine residues) into the predicted TEAD4-binding motif CATTCC (-507 to -502) and CEBPA-binding motif CCAAT (-241 to -237) to generate single (mut CATTCC, mut CCAAT) and double (mut both) mutant reporters. The luciferase assays showed that mutation of either the CATTCC or CCAAT motif markedly reduced the TEAD4- or CEBPA-mediated activation of the *Pdk4* promoter, respectively (Fig. EV3G). These results indicate that both predicted binding motifs contribute functionally to *Pdk4* promoter activation. However, mutation of these sites did not completely abolish the activation of the *Pdk4* promoter by TEAD4 and CEBPA. This suggests that additional potential TEAD4- or CEBPA-responsive elements may exist within the promoter region.

We have added these promoter truncation and point-mutation data to the revised manuscript and updated the corresponding text and figure legend accordingly.

8. Figure 7: Is Super-TDU really on target: do you see upregulation of YAP/TAZ target genes after treatment?

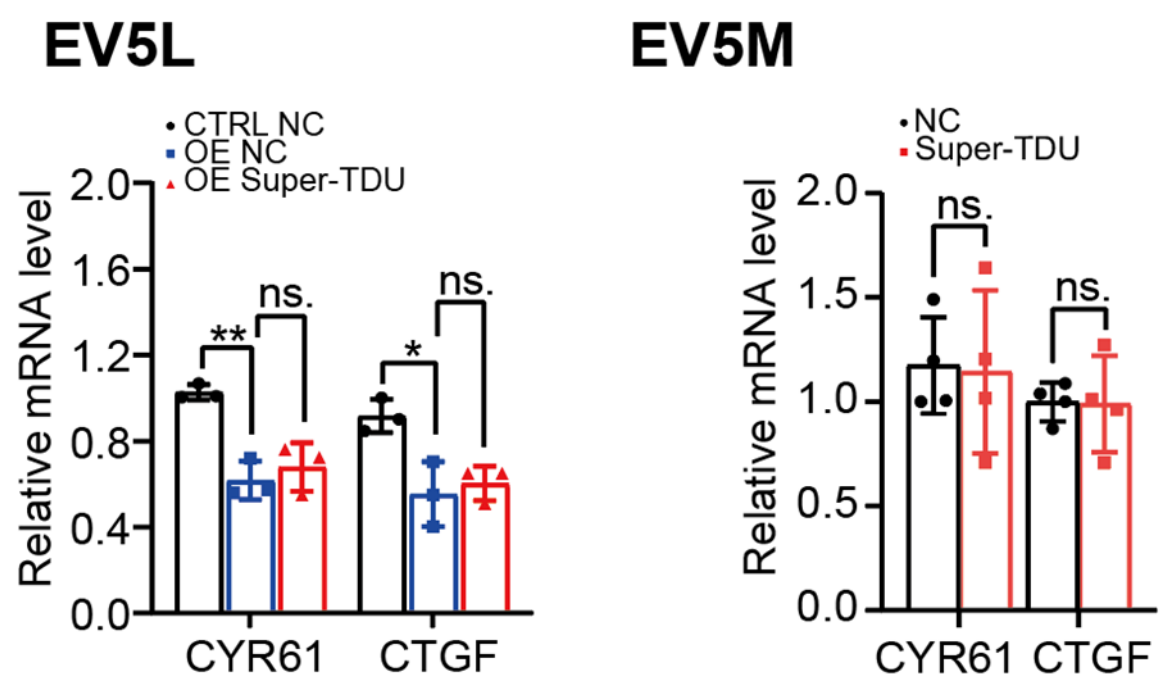

(EV5L, M) The mRNA levels of genes from the indicated groups, with expression levels normalized to those of β-actin (n=3–4). Data were presented as mean ± SD. For (EV5M) significance was determined by Welch t-test. For (EV5L), significance was determined using the one-way ANOVA. $*p < 0.05$, $**p < 0.01$, ns. indicates no significant difference.

Response: We thank the reviewer for this insightful suggestion, which helped us further clarify the mechanism underlying the therapeutic effect of Super-TDU in hepatic steatosis. We examined the expression of the canonical YAP/TAZ target genes *Ctgf* and *Cyr61* after ADV-Super-TDU treatment. In *Vgll4*-overexpressing livers, both genes were downregulated compared with controls (Fig. EV5L), consistent with the antagonistic effect of VGLL4 on YAP–TEAD signaling and the reduced YAP protein level observed in these livers (Fig. EV4A). However, ADV-Super-TDU treatment did not markedly alter *Ctgf* and *Cyr61* expression in either *Vgll4*-overexpressing or HFD-fed mice. Under *Vgll4*-overexpressing and HFD-fed conditions, where VGLL4 is elevated or shows an increasing trend (Fig. EV1E, F and Fig. 1K), TEAD4 may be preferentially engaged by VGLL4, thereby limiting the availability of TEAD4 for canonical YAP/TAZ–TEAD4 transcription. This may explain why Super-TDU treatment alleviated hepatic steatosis and decreased *Pdk4* expression without markedly altering *Ctgf* or *Cyr61* expression.

We have added these data and revised the text accordingly.

Minor comments:

9. Header line 149: Hepatocyte-specific.

Response: We have revised the header to avoid overstatement and have checked the related text to ensure that the specificity of the genetic model is described more accurately.

- ~~hepatocyte-specific~~ hepatic VGLL4 deletion

10. Sup. FigS1B: The authors claim upregulation of VGLL4 in patient-derived liver samples in a subset of MAFLD cases. Only 3 out of 3 cases seem to upregulate VGLL4, in the others the expression is similar to controls. Please quantifiy VGLL4 levels.

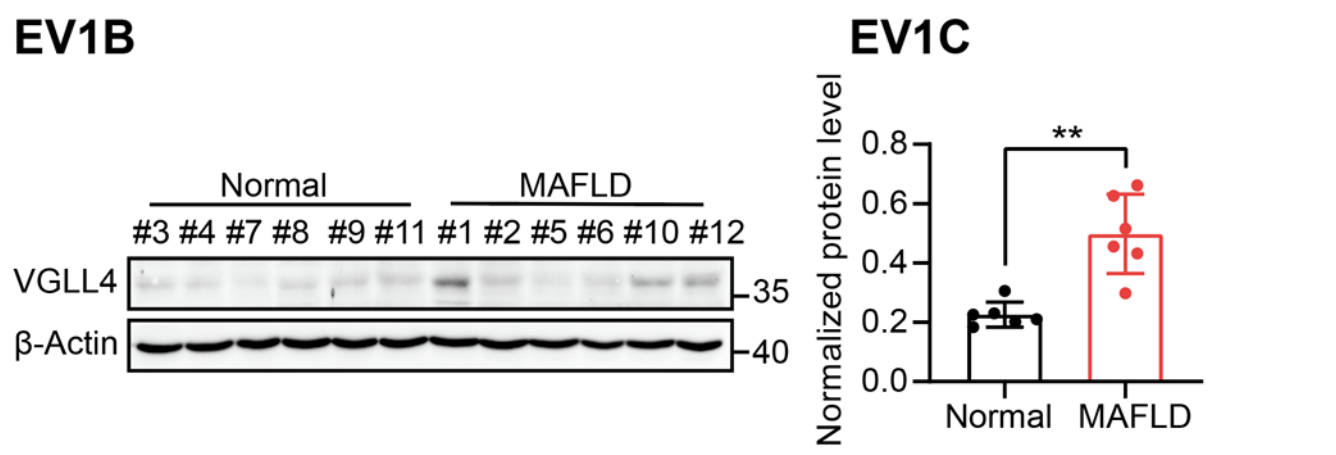

(EV1B) Western blot analysis of protein levels of VGLL4 in liver tissues from normal and MAFLD (n=6). (EV1C) The normalized protein levels of VGLL4, the protein levels were normalized to those of β-actin (n=6). Data were presented as mean ± SD. For (EV1C), significance was determined by Welch t-test. **$p < 0.01$ as indicated.

Response: We thank the reviewer for this helpful suggestion. We have quantified the VGLL4 Western blot signals in control and MAFLD liver samples. The analysis showed significantly increased VGLL4 protein levels in MAFLD samples (Fig. EV1C), consistent with the elevated VGLL4 mRNA expression observed in the public dataset (Fig. EV1A).

We have added this quantification to Fig. EV1C and revised the corresponding figure legend and text accordingly.

11. FigS1E: Statistics in legend is missing.

Response: We thank the reviewer for pointing this out. We have revised the figure legend and updated the statistical annotations after reanalyzing the data using Welch's t-test.

12. FigS2B: Co-staining with hepatocyte marker (e.g. HNF4) would be more convincing to show hepatocyte specific OE.

Response: Following the reviewer's suggestion, we performed co-immunofluorescence staining for FLAG and the hepatocyte marker HNF4α in liver sections from control and VGLL4-OE mice. As shown in the revised Appendix Fig. S1B, FLAG signals were readily detected in VGLL4-OE livers and mainly localized to HNF4α-positive hepatocytes.

We have added these co-staining data to Appendix Fig. S1B and revised the corresponding figure legend and text accordingly.

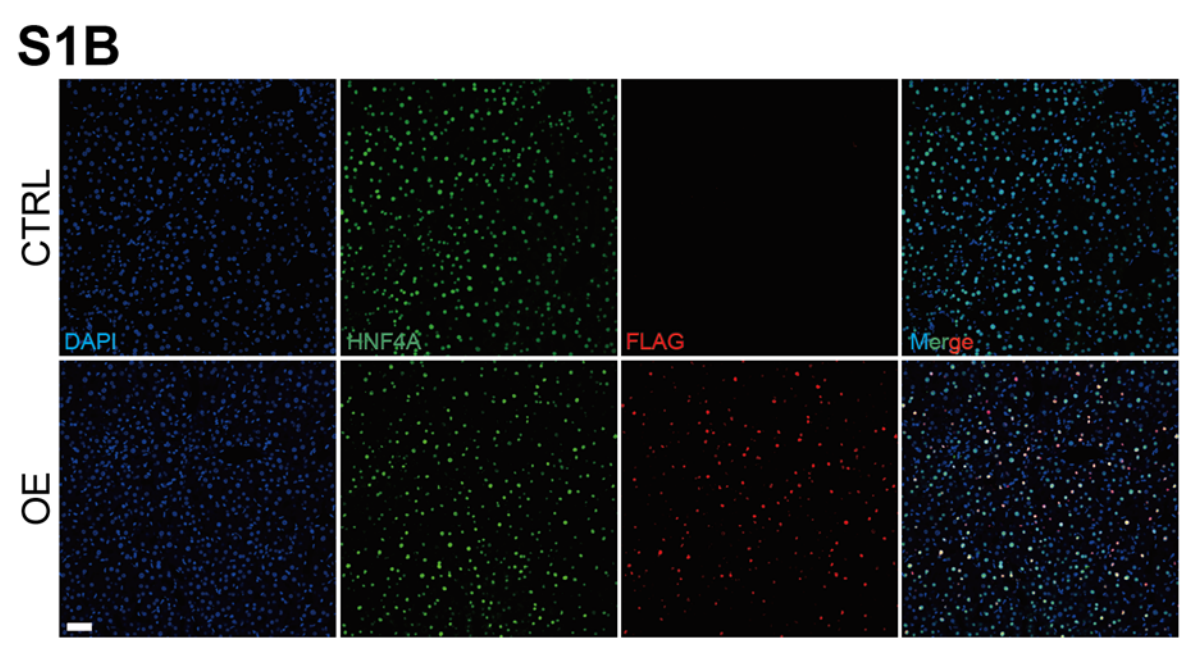

(S1B) Immunofluorescence staining for FLAG-VGLL4 and HNF4A in liver tissues from control and Vgll4 overexpression mice (scale bar, 50 μm).

13. Fig1I: Scale bar in all pics

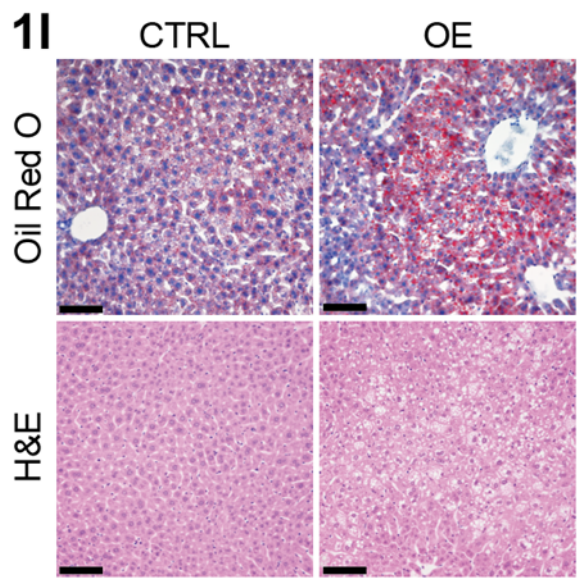

(1I) Representative images of Oil Red O (upper panel) and H&E (lower panel) stained liver sections (n=5). Scale bar, 100 μm.

Response: We thank the reviewer for pointing this out. The scale bar shown in Fig. 1I applies equally to all images in this panel.

Reviewer 3:

In this manuscript, Chen et al. identify VGLL4 as a regulator of MAFLD progression. Hepatocyte-specific overexpression of VGLL4 induced hepatic steatosis, whereas genetic ablation of VGLL4 in hepatocytes protected mice from high-fat diet-induced metabolic dysfunction. Mechanistically, the authors demonstrate that VGLL4 functions as a transcriptional activator by forming a complex with TEAD4 and CEBPA, thereby promoting PDK4 expression. In contrast, YAP overexpression disrupted the activity of this complex and attenuated steatosis. The findings are novel, mechanistically supported by the presented experimental data, and of broad relevance to hepatology and to the understanding of Hippo pathway signaling in metabolic liver disease. These findings are novel, mechanistically supported by the presented experimental data, and of broad relevance to hepatology and to the understanding of Hippo pathway signaling in metabolic liver disease. Importantly, the study expands the functional repertoire of VGLL4 beyond its established role as a YAP antagonist and positions it as a context-dependent transcriptional co-activator in hepatocytes. By demonstrating that VGLL4 promotes steatosis through a TEAD4-CEBPA complex that drives PDK4 expression, the work connects Hippo pathway components to metabolic flux control and mitochondrial substrate utilization. This mechanistic link provides a conceptual framework for how transcriptional cofactor dynamics can reprogram hepatocyte metabolism during MAFLD progression.

Response: Thank the reviewer for the valuable and constructive comments. The detailed and insightful review has greatly helped us improve the rigor and quality of our experiments and manuscript. We are grateful for the reviewer's careful evaluation and for the substantial contribution of these comments to improving our study.

Major remarks:

1. Human samples: there is no information about the fibrosis stage in the MAFLD patient samples where VGLL4 expression is analyzed. Without fibrosis staging, it's unclear whether increased VGLL4 correlates specifically with steatosis, or instead with fibrosis or inflammation.

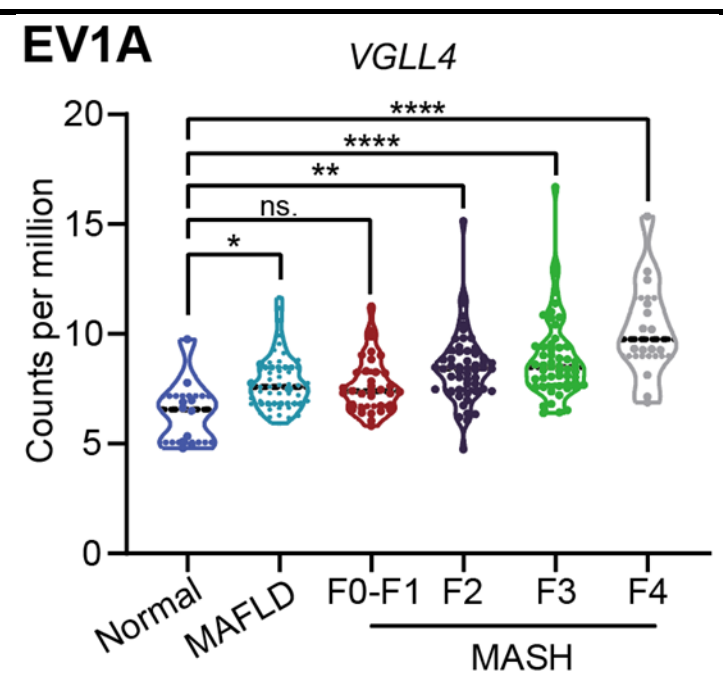

(EV1A) Violin plot depicting VGLL4 expression in normal liver tissues, MAFLD liver tissues, and MASH liver tissues stratified by fibrosis stage based on the GSE135251 dataset (n =10–206). For (EV1A), significance was determined using the one-way ANOVA. *p < 0.05, **p < 0.01, ****p< 0.0001, ns. indicates no significant difference.

Response: We thank the reviewer for raising this important point. Following the reviewer's suggestion, we reanalyzed this dataset based on the histologically annotated fibrosis stages (F0–F4)[13]. VGLL4 expression was increased in MAFLD samples compared with normal controls and was further elevated in MASH samples. These results suggest that VGLL4 upregulation may be associated with MAFLD/MASH progression, rather than being restricted to simple steatosis.

We have added the revised analysis to Fig. EV1A and modified the related text accordingly.

2. The use of Mx1-Cre complicates the interpretation of the claim that Vgll4 deletion in the liver inhibits HFD-induced steatosis. Mx1-Cre does not restrict recombination to hepatocytes; it induces deletion in hematopoietic stem cells and their progeny, including Kupffer cells, infiltrating macrophages, and other immune populations that critically regulate hepatic lipid accumulation under high-fat diet conditions. Given the well-established contribution of liver-resident and bone marrow-derived macrophages to inflammatory signaling, insulin sensitivity, and steatosis progression, the observed protection from steatosis could arise from altered immune cell function rather than a hepatocyte-intrinsic effect of Vgll4 loss. Without lineage-restricted deletion, bone marrow transplantation experiments, or hepatocyte-specific Cre controls, it is not possible to conclude that Vgll4 acts cell-autonomously within hepatocytes to regulate lipid accumulation. Therefore, attributing the steatosis phenotype solely to hepatic Vgll4 deletion is premature and requires additional compartment-specific validation. I thus suggest to repeat this experiment with another Cre line (Alb-CreER) or AAV-Cre.

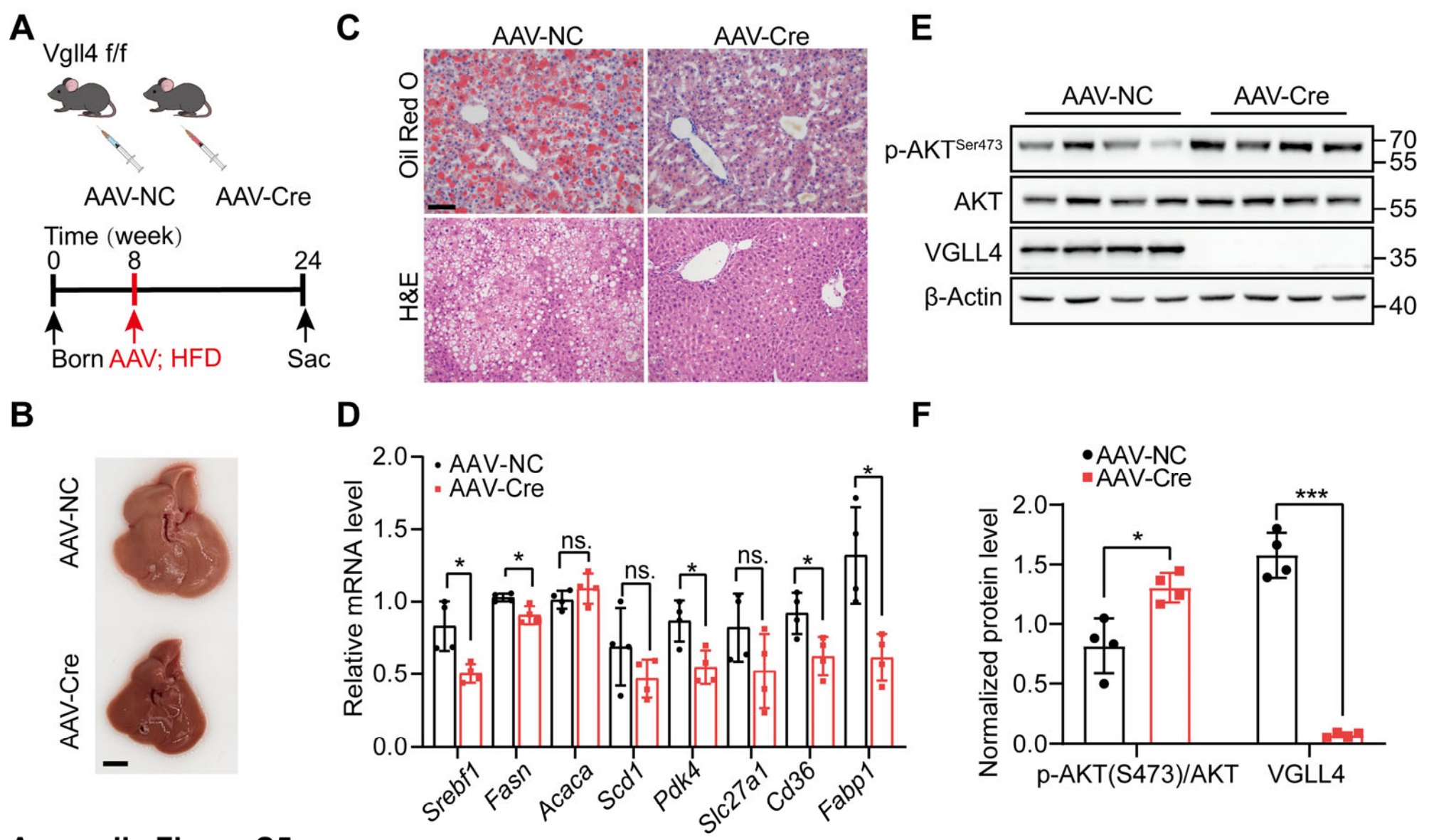

Appendix Figure S5

Appendix Figure S5 AAV2/8-TBG-Cre-mediated *Vgll4* deletion protects adult mice from HFD-induced hepatic steatosis and impaired insulin signaling

(A) Schematic diagram of animal experiments. *Vgll4*$^{f/f}$ mice were injected with AAV2/8-TBG-Cre (AAV-Cre) or AAV negative control vector (AAV-NC) through the tail vein, and the metabolic phenotype was assessed. (B) Representative images of the livers (n=3). Scale bar, 5 mm. (C) Representative images of Oil Red O (upper panel) and H&E (lower panel) stained liver sections (n=3). Scale bar, 100 μm. (D) The mRNA levels of genes involved in fatty acid synthesis and uptake from the indicated groups, with expression levels

normalized to those of β-actin (n=4). (E, F) Mice were intraperitoneally injected with insulin following 4 hours fasting. Activation of the insulin signaling pathway in the livers of mice from the indicated groups was detected by Western blotting (E), the protein levels of insulin signaling-related molecules were normalized to β-actin (F) (n=4). Data were presented as mean ± SD. For (D) and (F), significance was determined by Welch t-test. *p < 0.05, ***p < 0.001, ns. indicates no significant difference.

Response: We thank the reviewer for this important and detailed comment. In fact, we had also recognized this limitation during the course of the study and therefore initiated an independent validation experiment using a more hepatocyte-targeted Cre delivery system. Specifically, *Vgll4$^{f/f}$* mice were injected with AAV2/8-TBG-Cre, while control *Vgll4$^{f/f}$* mice were injected with the corresponding AAV2/8-TBG control vector. The TBG promoter, together with the AAV2/8 serotype, was used to preferentially drive Cre expression in hepatocytes and thereby reduce the potential confounding effects from hematopoietic or immune-cell recombination. After HFD feeding, AAV2/8-TBG-Cre-mediated *Vgll4* deletion produced phenotypes consistent with those observed in the Mx1-Cre model. Hepatocyte-targeted *Vgll4* deletion alleviated HFD-induced hepatic lipid accumulation and improved insulin resistance-related metabolic phenotypes (Fig.EV6). These results support the conclusion that loss of *Vgll4* in hepatocytes contributes substantially to the protection against HFD-induced steatosis.

We now describe the Mx1-Cre data more cautiously as hepatic *Vgll4* deletion and revised the related text, figure legend, and Discussion accordingly.

3. In addition, VGLL4 overexpression starts at 4 weeks of age (Fig. 1B), but this is not clearly mentioned in the text. Why did they start so early? Mice are not fully adult at that age and it would be better to induce VGLL4 once the mice are adult in order to exclude the possibility of effects on liver development.

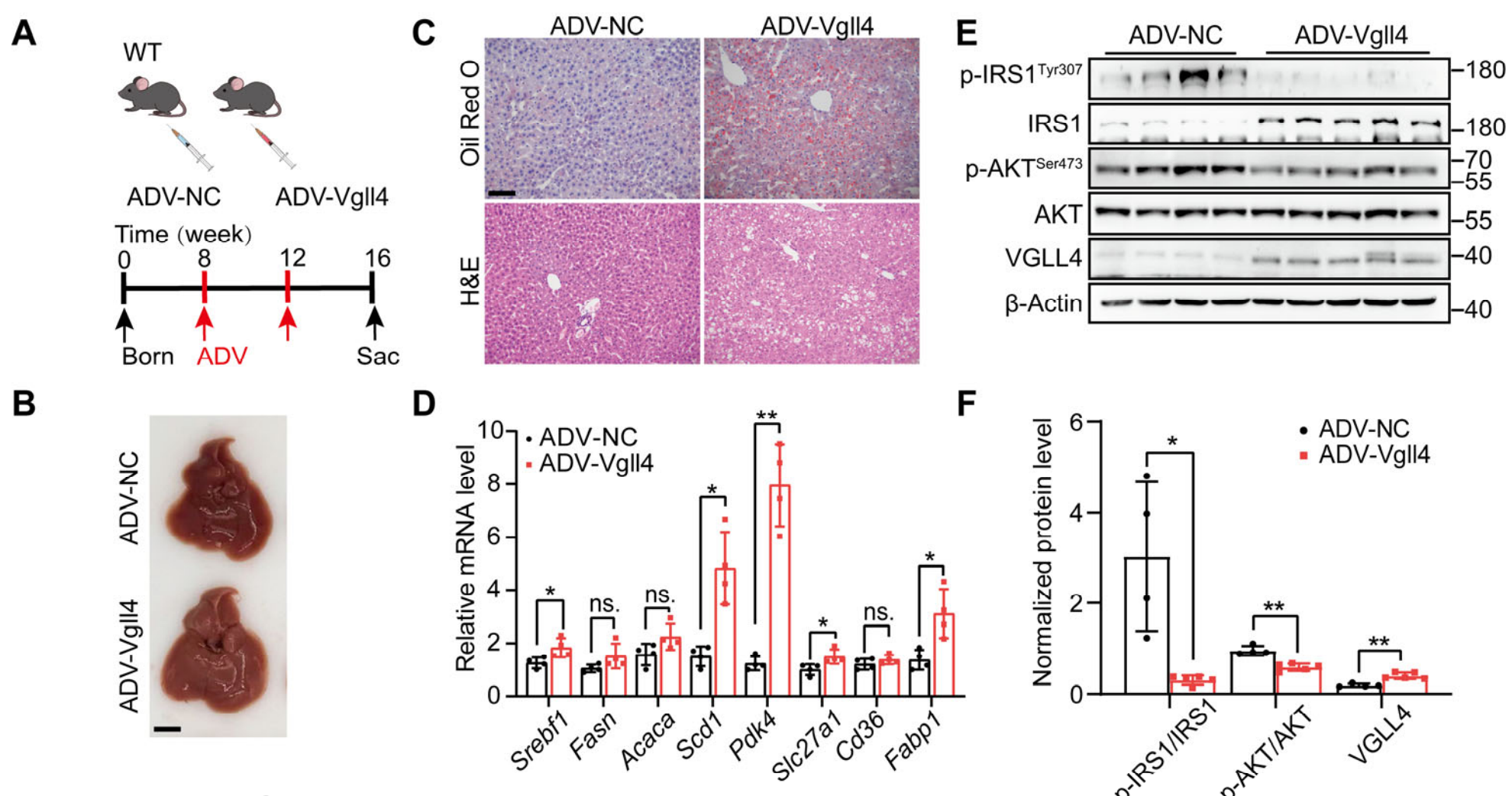

Appendix Figure S3

Appendix Figure S3 Adenovirus-mediated Vgll4 overexpression in adult mice recapitulates hepatic steatosis and impaired insulin signaling

(A) Schematic diagram of animal experiments. WT mice were injected with ADV-Vgll4 or ADV negative control vector (ADV-NC) through the tail vein, and the metabolic phenotype was assessed. (B)

Representative images of the livers (n=3). Scale bar, 5 mm. (C) Representative images of Oil Red O (upper panel) and H&E (lower panel) stained liver sections (n=3). Scale bar, 100 μm. (D) The mRNA levels of genes involved in fatty acid synthesis and uptake from the indicated groups, with expression levels normalized to those of β-actin (n=4). (E, F) Mice were intraperitoneally injected with insulin following 4 hours fasting. Activation of the insulin signaling pathway in the livers of mice from the indicated groups was detected by Western blotting (E), the protein levels of insulin signaling-related molecules were normalized to β-actin (F) (n=4–5). Data were presented as mean ± SD. For (D) and (F), significance was determined by Welch t-test. *$p < 0.05$, **$p < 0.01$, ns. indicates no significant difference.

Response: We thank the reviewer for raising this important concern. In the *LAP-tTA; TetO-Vgll4* model, *LAP-tTA* acts as a constitutive driver, and *Vgll4* overexpression is therefore initiated at an early age under doxycycline-free conditions. We agree that early *Vgll4* expression may raise concerns about potential effects on liver development and subsequent hepatic metabolism.
To address this issue, we performed an additional adult-onset *Vgll4* overexpression experiment. Specifically, 8-week-old WT mice were injected with ADV-*Vgll4* or control ADV-NC. In this adult-stage model (Appendix Fig. S3A), *Vgll4* overexpression still induced hepatic lipid accumulation (Appendix Fig. S3B, C), increased the expression of lipid metabolism-related genes (Appendix Fig. S3D), and impaired hepatic insulin signaling (Appendix Fig. S3E, F), consistent with the phenotype observed in the original transgenic model. These results support that VGLL4 overexpression is sufficient to promote hepatic steatosis and insulin signaling impairment after liver development is largely complete.

We have added these data to the revised manuscript and revised the related text accordingly.

4. It is unexpected that PDK4 alone would be sufficient to induce hepatic steatosis in VGLL4 overexpressing mice. PDK4 does not directly activate canonical lipogenic transcriptional programs such as SREBP1c or ChREBP, nor is it known to suppress key Î²-oxidation enzymes or impair very-low-density lipoprotein secretion. Its primary function is to modulate mitochondrial carbon flux rather than to drive lipid synthesis or storage. How could PDK4 by itself explain the steatosis effects driven by VGLL4 expression? This requires validation, as additional metabolic or inflammatory perturbations would likely be required to produce this phenotype. Do the authors speculate that PDK4 could induce the expression of lipogenic genes? This should be more explicitely discussed.

Response: We agree that PDK4 should not be interpreted as the only downstream mediator that explains all metabolic changes induced by *Vgll4* overexpression. Our screening strategy for VGLL4-regulated downstream targets may have missed additional genes or pathways that also contribute to lipid accumulation (Fig. 5A and Fig. EV3A, B). Nevertheless, our experimental data support an important functional role of PDK4 in *Vgll4*-induced lipid accumulation. At the cellular level, PDK4 overexpression markedly promoted lipid droplet accumulation and increased the expression of key enzymes involved in fatty acid synthesis (Fig. 5D, E). In vivo, PDK4 knockdown substantially alleviated the hepatic steatosis phenotype induced by *Vgll4* overexpression (Fig. 6). These results indicate that, although PDK4 is unlikely to be the only

downstream mediator of VGLL4-induced steatosis, it represents a critical metabolic effector in this process.

Regarding the possible mechanism, we agree that PDK4 is not a classical transcription factor and is unlikely to directly activate canonical lipogenic transcriptional programs. However, previous studies support the idea that PDK4 can promote hepatic lipid accumulation indirectly through metabolic remodeling. PDK4 inhibits the pyruvate dehydrogenase complex and thereby limits pyruvate entry into mitochondrial oxidation, which may alter glucose–lipid metabolic coupling and nutrient-sensitive signaling pathways.[1,3,14,15] In NASH models, hepatic PDK4 expression was reported to be increased in human NASH samples, MCD diet-fed mice, and oleic acid-treated hepatocytes; importantly, *Pdk4* deficiency alleviated hepatic steatosis and reduced the expression of genes involved in fatty acid uptake, lipid synthesis, and gluconeogenesis.[1] In addition, pharmacological inhibition of hepatic PDK activity or PDK2/PDK4 deficiency in diet-induced obese mice reduced hepatic steatosis and attenuated ChREBP-mediated lipogenesis, accompanied by downregulation of lipogenic enzymes such as ACC1, FAS, and SCD1.[2] In addition to its canonical role in regulating PDH activity, PDK4 has also been reported to promote hepatic lipid accumulation through kinase-dependent regulation of mitochondria-associated ER membranes (MAMs).[5] Together with our cellular data, these findings suggest that PDK4 may contribute to hepatic lipid accumulation through multiple mechanisms. However, whether PDK4 directly regulates lipogenesis through PDH/TCA-cycle-independent or non-canonical kinase-dependent pathways remains to be further explored.

We have further incorporated this point into the revised Discussion section.

Minor remarks:

5. Check the gene, mRNA and protein nomenclatures and adapt the text accordingly.

Response: We thank the reviewer for pointing this out. We have carefully checked the nomenclature of genes, mRNAs, and proteins throughout the manuscript and revised the text accordingly. Specifically, gene and mRNA names have been formatted according to species-specific nomenclature, with mouse genes written in italicized lowercase format, such as *Vgll4* and *Pdk4*, and human genes written in italicized uppercase format, such as *VGLL4* and *PDK4*. Protein names have been written in non-italicized uppercase format, such as VGLL4 and PDK4. The figure legends and supplementary materials have also been checked and corrected where necessary.

6. The authors claim "VGLL4 functions as an enhancer". However, enhancers are genetic motifs, not proteins. The correct nomenclature would be VGLL4 acts as a transcriptional activator instead as a transcriptional repressor.

Response: We thank the reviewer for pointing out this inappropriate wording. We have carefully checked the manuscript for similar wording issues and revised the text accordingly.

The sentence has been revised as follows:

- "Mechanistically, VGLL4 facilitates the formation of a transcriptional ternary complex with TEAD4 and CEBPA, thereby promoting *Pdk4* transcription."

Reference

[1] Zhang M, Zhao Y, Li Z, Wang C. Pyruvate dehydrogenase kinase 4 mediates lipogenesis and contributes to the pathogenesis of nonalcoholic steatohepatitis. Biochem Biophys Res Commun 2018;495:582–6. https://doi.org/10.1016/j.bbrc.2017.11.054.

[2] Wu C-Y, Tso S-C, Chuang JL, Gui W-J, Lou M, Sharma G, et al. Targeting hepatic pyruvate dehydrogenase kinases restores insulin signaling and mitigates ChREBP-mediated lipogenesis in diet-induced obese mice. Mol Metab 2018;12:12–24. https://doi.org/10.1016/j.molmet.2018.03.014.

[3] Zhang S, Hulver MW, McMillan RP, Cline MA, Gilbert ER. The pivotal role of pyruvate dehydrogenase kinases in metabolic flexibility. Nutr Metab (Lond) 2014;11:10. https://doi.org/10.1186/1743-7075-11-10.

[4] Jeoung NH. Pyruvate Dehydrogenase Kinases: Therapeutic Targets for Diabetes and Cancers. Diabetes Metab J 2015;39:188–97. https://doi.org/10.4093/dmj.2015.39.3.188.

[5] Thoudam T, Chanda D, Lee JY, Jung M-K, Sinam IS, Kim B-G, et al. Enhanced Ca2+-channeling complex formation at the ER-mitochondria interface underlies the pathogenesis of alcohol-associated liver disease. Nat Commun 2023;14:1703. https://doi.org/10.1038/s41467-023-37214-4.

[6] Jiao S, Wang H, Shi Z, Dong A, Zhang W, Song X, et al. A peptide mimicking VGLL4 function acts as a YAP antagonist therapy against gastric cancer. Cancer Cell 2014;25:166–80. https://doi.org/10.1016/j.ccr.2014.01.010.

[7] Zhang W, Gao Y, Li P, Shi Z, Guo T, Li F, et al. VGLL4 functions as a new tumor suppressor in lung cancer by negatively regulating the YAP-TEAD transcriptional complex. Cell Res 2014;24:331–43. https://doi.org/10.1038/cr.2014.10.

[8] Zhang Y, Shen H, Withers HG, Yang N, Denson KE, Mussell AL, et al. VGLL4 selectively represses YAP-dependent gene induction and tumorigenic phenotypes in breast cancer. Sci Rep 2017;7:6190. https://doi.org/10.1038/s41598-017-06227-7.

[9] Feng X, Wang Z, Wang F, Lu T, Xu J, Ma X, et al. Dual function of VGLL 4 in muscle regeneration. The EMBO Journal 2019;38:e101051. https://doi.org/10.15252/embj.2018101051.

[10] Feng X, Wang Z, Wang F, Lu T, Xu J, Ma X, et al. Dual function of VGLL4 in muscle regeneration. EMBO J 2019;38:e101051. https://doi.org/10.15252/embj.2018101051.

[11] Suo J, Feng X, Li J, Wang J, Wang Z, Zhang L, et al. VGLL4 promotes osteoblast differentiation by antagonizing TEADs-inhibited Runx2 transcription. Sci Adv 2020;6:eaba4147. https://doi.org/10.1126/sciadv.aba4147.

[12] Shi T, Lv W, Zhu Q, Liu H, Wang Y, Wu H, et al. 1 adipose tissue VGLL4 protects hepatic insulin sensitivity through 2 SCD1-derived palmitoleic acid n.d.

[13] Govaere O, Cockell S, Tiniakos D, Queen R, Younes R, Vacca M, et al. Transcriptomic profiling across the nonalcoholic fatty liver disease spectrum reveals gene signatures for steatohepatitis and fibrosis. Science Translational Medicine 2020;12:eaba4448. https://doi.org/10.1126/scitranslmed.aba4448.

[14] Martínez-Reyes I, Chandel NS. Mitochondrial TCA cycle metabolites control physiology and disease. Nat Commun 2020;11:102. https://doi.org/10.1038/s41467-019-13668-3.

[15] Reshef L, Olswang Y, Cassuto H, Blum B, Croniger CM, Kalhan SC, et al. Glyceroneogenesis and the triglyceride/fatty acid cycle. J Biol Chem 2003;278:30413–6. https://doi.org/10.1074/jbc.R300017200.

Dear Dr. Zhang,

Thank you for the submission of your revised manuscript to our editorial offices. I have now received the reports from the three referees that were asked to re-evaluate the study, you will find below. As you will see, the referees now support publication of your study in EMBO reports. Referee #2 has remaining concerns or suggestions to improve the manuscript, I ask you to address in a final revised manuscript.

Moreover, I have the editorial requests below. Please also provide a final p-b-p-response to the remaining referee concerns and the editorial requests.

Editorial requests:

- Please provide a more comprehensive title with less abbreviations and avoiding the phrase 'axis' (with not more than 100 characters including spaces).

- We now use CRediT to specify the contributions of each author in the journal submission system. CRediT replaces the author contribution section. Please use the free text box to provide more detailed descriptions and do NOT provide your final manuscript text file with an author contributions section. See also our guide to authors (section 'Author contributions'): https://link.springer.com/journal/44319/submission-guidelines#cms-Revised-submissions

- Please provide an ORCID linked to the corresponding author Jian Wan in the submission system. All corresponding authors need to provide and ORCID. Otherwise, we will not procced with the manuscript.

- Please check again that the number "n" for how many independent experiments were performed, their nature (biological versus technical replicates), the bars and error bars (e.g. SEM, SD) and the test used to calculate p-values is indicated in the respective figure legends (main and EV figures). Please also check that all the p-values are explained in the legend, and that these fit to those shown in the figure. Please provide statistical testing where applicable. Please avoid the phrase 'independent experiment' but clearly state if these were biological or technical replicates. Please also indicate (e.g. with n.s.) if testing was performed, but the differences are not significant. In case n=2, please show the data as separate datapoints without error bars and statistics. See also:

https://link.springer.com/journal/44319/submission-guidelines#cms-Figure-and-data-presentation

If n<5, please show single datapoints for diagrams. Moreover:
- Please note that the exact p values are not provided in the legends of 1C-H,J,L; 2A,B,D,F; 3B,D-G,K,L,M,P,R; 4B,E; 5B,C,E,G,I,J,L,M,N,O; 6C-H,K-N,P,R; 7B,D-I,K; EV-1A,C,F; EV-2A,E,J; EV-3C,E,F,G,I,J; EV-4D,I,J; EV-5D-I,K,L; S1-E; S3-D,F; S4-B-D; S5-D,F; S6-C,E,F,G.
- Please indicate the statistical test used for data analysis in the legends of 1M; 4F; 5A.

- Table S6 is a dataset. Please name this file Dataset EV1 and upload it as dataset file. Please adjust all callouts (Dataset EV1).

- Please move the primer and antibody information presently part of the Appendix (Tables S2-S5) to the Reagents & Tools table. Then please remove these tables from the Appendix. Please also remove the instructions from the Reagents & Tools Table. Finally, please update the callouts and also add further callouts to the Reagents & Tools Table where appropriate.

- Please provide the Appendix file as pdf.

- Figure EV4 is titled 'Movie EV4', but it is not a movie file. Please check.

- Please add scale bars of similar style and thickness to microscopic images, using clearly visible black or white bars (depending on the background). Please place these in the lower right corner of the images themselves. Please do not write on or near the bars in the image but define the size in the respective figure legend. Presently, many scale bars are hard to see. Please improve.

- Please remove the legends for the movies from the manuscript text file. Please provide separate legends for each movie as text file ZIPped up with its respective movie file so that we have 4 zip folders (Movie EV1 - Movie EV4).

- Please make sure that all the funding information is also entered into the online submission system and that it is complete and similar to the one in the acknowledgement section of the manuscript text file. The Shanghai Municipal Science and Technology Major Project, and the Shanghai Leading Talents Program need to be added as separate funders in the submission system.

- Please confirm that for all Western blot panels shown the loading control was run on the same gel as the other proteins

detected. Please note that we discourage comparisons between samples on different gels/blots, even if the samples derive from one experiment, as confounding factors reduce comparability. If unavoidable, the figure legend must state that the samples derive from the same experiment and that gels/blots were processed in parallel. If a 'representative' loading control is shown for multiple gels/blots, the intra-gel controls should be shown in the source data files, and the figure legends should describe the data displayed accurately. See our author guidelines:

https://link.springer.com/journal/44319/submission-guidelines#cms-Figure-and-data-presentation (section 'Electrophoretic gels and blots').

- Please name the section 'Availability of data and materials' 'Data Availability' and provide final links for these datasets. Please make sure the datasets are public latest upon online publication of the manuscript.

- Thanks for providing the source data. Please provide the source data for the Appendix figure combined in one folder. Moreover, the source data file for Fig. 1B has 6 values for each ctrl condition, although the legend states n=8. The source data file for Fig. 1C contains 9 values for the ctrl condition (with the first value duplicated), although the legend states n=8. Please check. Please carefully go through the source data file and make sure that the values correspond to the n indicated in the legends!

In addition, I would need from you uploaded separately:
- a short, two-sentence summary of the manuscript (not more than 35 words).
- two to four short (!) bullet points highlighting the key findings of your study (two lines each).
- a schematic summary figure as separate file that provides a sketch of the major findings (not a data image) in jpeg or tiff format (with the exact width of 550 pixels and the exact height of 300 pixels) that can be used as a visual synopsis on our website. Please upload this separately and remove the graphical abstract from the manuscript text file.

I look forward to seeing the further revised version of your manuscript when it is ready. Please let me know if you have questions regarding the revision.

Best,

Achim Breiling
Senior Editor
EMBO Reports

Referee #1:

Congrats for this nice work.

Referee #2:

In the revised version, the authors have addressed most of my points. However, one critical point was not addressed: the genome-wide co-occupancy of TEAD/VGLL4 and CEBPA. This is an absolutely critical experiment and the argumentation that "antibody compatibility limitations" are the reason for not having performed the experiment is not convincing. There are dozens of publicly available CEBPA ChIP-Seq datasets, indicating that suitable antibodies exist.

The provided CUT&TAG analysis is very superficial: how many peaks were called, etc. is not explained. Furthermore, it is impossible to judge whether the signal shown for TEAD at the PDK4 promoter is good compared to other canonical TEAD targets (e.g. CTGF, AMOTL2) or whether this is close to background. The authors do not show an IgG control, which is required to judge this and would need to be provided.

Also reviewer #3 touches on this point because it is highly unlikely that a specific trimeric complex exists to regulate one gene, namely PDK4. Either the authors substantially tone down the claim of the trimeric complex regulating PDK4, or they perform additional CUT&TAG experiments.

Referee #3:

The authors added a substantial amount of relevant new data, which addressed my concerns.

EMBOR-2026-63711-V3 _Point-to-point response

Below are our detailed point-to-point responses for each reviewer.

Referee 1:

Congrats for this nice work.

Response: We sincerely thank the reviewer for the positive feedback and recommendation. We greatly appreciate the time and effort devoted to evaluating our manuscript, as well as the valuable comments and suggestions that have helped us substantially improve its quality. We are truly grateful for the reviewer’s recognition and support.

Referee 2:

1. In the revised version, the authors have addressed most of my points. However, one critical point was not addressed: the genome-wide co-occupancy of TEAD/VGLL4 and CEBPA. This is an absolutely critical experiment and the argumentation that "antibody compatibility limitations" are the reason for not having performed the experiment is not convincing. There are dozens of publicly available CEBPA ChIP-Seq datasets, indicating that suitable antibodies exist.

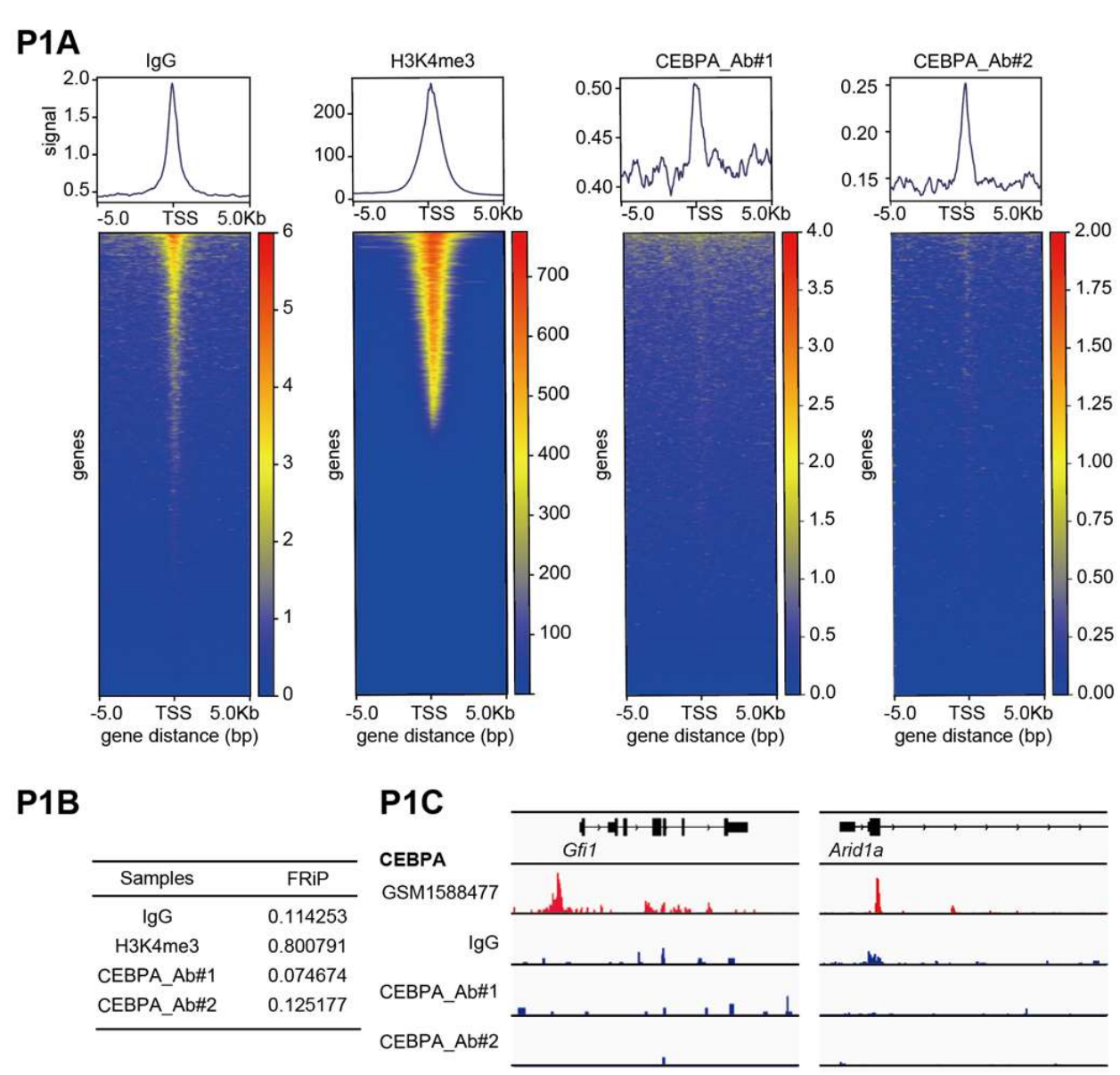

Figure P1. Quality-control assessment of CUT&Tag datasets generated using two CEBPA antibodies.

(A) Average signal profiles and corresponding heatmaps showing CUT&Tag enrichment across a 10-kb window centered on transcription start sites (TSS; ±5 kb) for the IgG control, H3K4me3 positive control, CEBPA antibody #1 (CEBPA_Ab#1), and CEBPA antibody #2 (CEBPA_Ab#2). (B) Fraction of reads in peaks (FRiP) values for the IgG, H3K4me3, CEBPA_Ab#1, and CEBPA_Ab#2 datasets. (C) Representative genome-browser tracks at the *Gfi1* and *Arid1a* loci.

Response: We apologize that our previous response may not have sufficiently addressed the reviewer's concern regarding this experiment. We fully agree that investigating the genome-wide co-occupancy of TEAD/VGLL4 and CEBPA represents an important biological question. During the previous revision, we had attempted CEBPA CUT&Tag, and in the present revision, we provide these results to more clearly demonstrate the outcome of these experiments.

We attempted CEBPA CUT&Tag using two commercially available antibodies that have been validated for ChIP-seq or ChIP-qPCR (CEBPA_Ab#1: Abcam ab317442; CEBPA_Ab#2: Proteintech 84668-4-RR). Although the H3K4me3 positive control showed robust TSS enrichment and a high FRiP score, confirming that the CUT&Tag workflow was technically successful, both CEBPA datasets displayed weak enrichment and lower FRiP scores comparable to the IgG background (Fig. P1 A and B). Moreover, neither antibody produced discernible enrichment at the *Gfi1* and *Arid1a* loci, two well-established CEBPA-binding sites, as supported by prominent enrichment signals for both loci in the public CEBPA ChIP-seq dataset (GSM1588477) (Fig. P1C), consistent with previous reports (Lidonnici *et al*, 2010; Sun *et al*, 2016).

Therefore, although we agree that genome-wide TEAD/VGLL4–CEBPA co-occupancy analysis would be biologically informative, the quality of the CEBPA CUT&Tag datasets was insufficient to support a reliable integrative analysis.

2. The provided CUT&Tag analysis is very superficial: how many peaks were called, etc. is not explained. Furthermore, it is impossible to judge whether the signal shown for TEAD at the PDK4 promoter is good compared to other canonical TEAD targets (e.g. CTGF, AMOTL2) or whether this is close to background. The authors do not show an IgG control, which is required to judge this and would need to be provided.

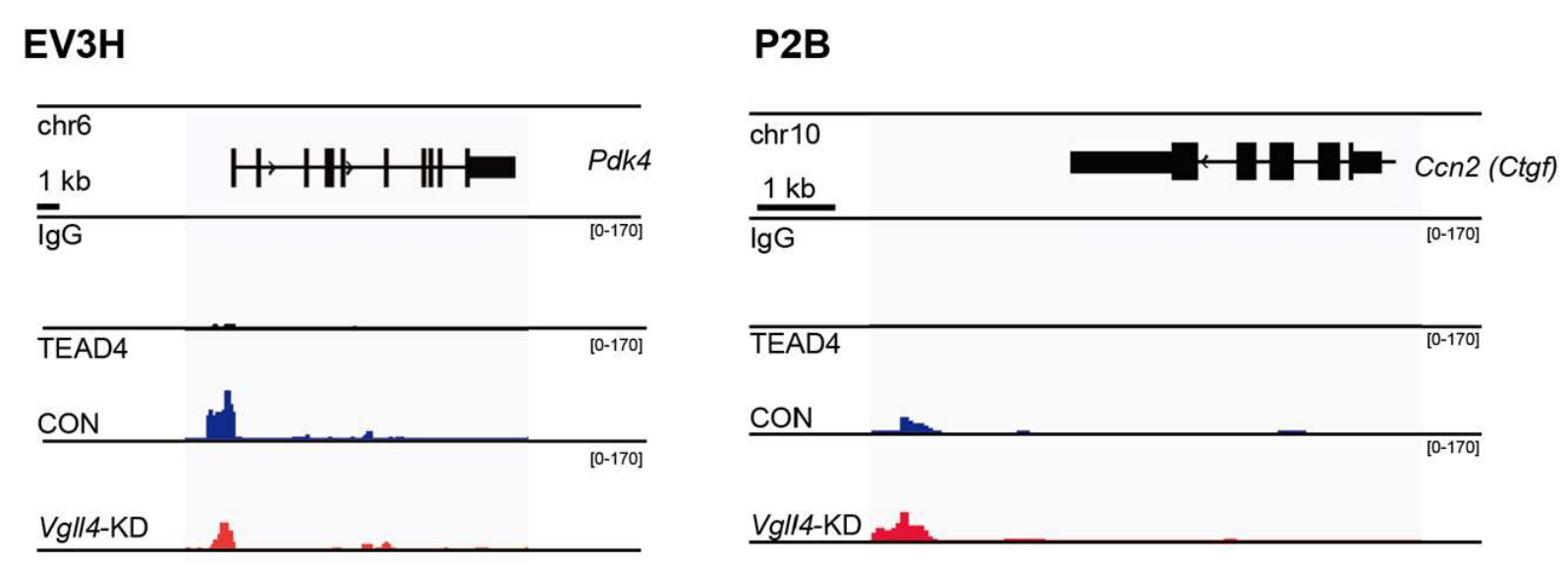

(EV3H) Genome-browser tracks showing TEAD4 CUT&Tag signals at the *Pdk4* locus in control (CON) and *Vgll4*-knockdown (*Vgll4*-KD) cells, with IgG as the negative control. (P2B) Genome-browser tracks showing TEAD4 CUT&Tag signals at the *Ccn2 (Ctgf)* locus in CON and *Vgll4*-KD cells, with IgG as the negative control.

Response: We thank the reviewer for this important comment. Using the same peak-calling criteria, a total of 11,394 TEAD4 peaks were identified in control AML12 cells, whereas 9,068 peaks were identified in *Vgll4* knockdown cells. A peak associated with the *Pdk4* locus was identified in both conditions. The called regions were highly overlapping, spanning chr6:5,496,154–5,497,275 in the control sample and chr6:5,496,232–5,497,032 in the *Vgll4* knockdown sample. These results confirm that the *Pdk4*-associated region passed the peak-calling criteria in both samples. To assess nonspecific background and the relative magnitude of the TEAD4 signal, we added an IgG CUT&Tag track generated from control AML12 cells and compared the *Pdk4* locus with the canonical TEAD target *Ccn2* (*Ctgf*) (Fig. EV3H and Fig. P2B). All displayed tracks were spike-in normalized. In control cells, TEAD4 enrichment at the *Pdk4*-associated region was clearly above the IgG background and was within a similar range to that observed at *Ccn2*. Following *Vgll4* knockdown, the normalized TEAD4 signal at the *Pdk4* locus was reduced, whereas TEAD4 enrichment at *Ccn2* was retained. Thus, the reduction at *Pdk4* is unlikely to reflect a nonspecific loss of CUT&Tag signal and instead supports a locus-selective effect of *Vgll4* depletion on TEAD4 chromatin occupancy.

The reduction in TEAD4 occupancy at the *Pdk4* regulatory region was independently confirmed by TEAD4 ChIP-qPCR (Fig. EV3I). We have added the

peak-calling results, the IgG control, and the corresponding description to the revised manuscript.

3. Also reviewer #3 touches on this point because it is highly unlikely that a specific trimeric complex exists to regulate one gene, namely PDK4. Either the authors substantially tone down the claim of the trimeric complex regulating PDK4, or they perform additional CUT&TAG experiments.

Response: We thank the reviewer for this thoughtful and constructive suggestion. We agree that, if VGLL4-enhanced TEAD4–CEBPA cooperation represents a broader regulatory mechanism, it is unlikely to be restricted to *Pdk4*. Profiling TEAD4 and CEBPA occupancy across the genome would therefore be valuable for identifying additional co-regulated genes, particularly those involved in lipid metabolism, and, in conjunction with our current evidence, for further supporting the presence and functional relevance of the ternary complex at additional genomic loci.

However, we were unable to obtain CEBPA CUT&Tag data of sufficient quality for a reliable co-occupancy analysis. Consequently, the present study cannot define the full target repertoire of VGLL4-enhanced TEAD4–CEBPA cooperation. In response to the reviewer's concern, we have substantially toned down our original claim and revised the manuscript to avoid implying that a broad VGLL4/TEAD4/CEBPA transcriptional program has been established. Specifically, we no longer conclude that a chromatin-bound trimeric complex has been definitively demonstrated to regulate *Pdk4*. Instead, we have limited our interpretation to a promoter-level model in which VGLL4 facilitates TEAD4–CEBPA cooperation at the *Pdk4* locus. This interpretation is supported by biochemical evidence showing that VGLL4 enhances the interaction between TEAD4 and CEBPA, chromatin-based assays supporting VGLL4-dependent TEAD4 and CEBPA occupancy at the *Pdk4* promoter, and promoter-reporter assays demonstrating their functional contribution to *Pdk4* transcriptional activation.

Whether VGLL4-enhanced TEAD4–CEBPA cooperation extends to additional lipid metabolism-related genes remains an important direction for future investigation. We sincerely thank the reviewer for highlighting this valuable question.

Referee 3:

The authors added a substantial amount of relevant new data, which addressed my concerns.

Response: We sincerely thank the reviewer for the positive assessment of our revised manuscript. We are pleased that the additional data and corresponding revisions have adequately addressed the reviewer’s concerns. We greatly appreciate the reviewer’s constructive comments, which have helped us substantially improve the quality and rigor of our work.

Dr. Lei Zhang
Shanghai Jiao Tong University
Sheng Yushou Center of Cell Biology and Immunology
Department of Urology, Shanghai Sixth People' s Hospital Affiliated to Shanghai Jiao Tong University School of Medicine, Shanghai Eastern Institute of Urologic Reconstruction, Shanghai Jiao Tong Uni
School of Life Science, Hangzhou Institute for Advanced Study, University of Chinese Academy of Sciences, Hangzhou 310024, China.
China

Dear Dr. Zhang,

Thank you for the submission of your further revised manuscript to our editorial offices. I have now received the report from the referee that was asked to re-evaluate the study, you will find below. As you will see, the referee now fully supports the publication of your study. I also consider the editorial requests as fully addressed.

I am thus very pleased to accept your manuscript for publication in the next available issue of EMBO reports. Thank you for your contribution to our journal.

Your manuscript will be processed for publication by EMBO Press. It will be copy edited and you will receive page proofs prior to publication. Please note that you will be contacted by Springer Nature Author Services to complete licensing and payment information.

You may qualify for financial assistance for your publication charges - either via a Springer Nature fully open access agreement or an EMBO initiative. Check your eligibility: https://link.springer.com/journal/44319/how-to-publish-with-us

Should you be planning a Press Release on your article, please notify the production team via this form in order to coordinate publication and release dates: https://forms.cloud.microsoft/e/eH4A8JVP6g

If you have any questions, please do not hesitate to contact the Editorial Office. Thank you for your contribution to EMBO Reports.

Yours sincerely,

Achim Breiling
Senior Editor
EMBO Reports

Referee #2:

The authors have addressed my concerns.

>>> Please note that it is EMBO Reports policy for the transcript of the editorial process (containing referee reports and your response letter) to be published as an online supplement to each paper. If you do NOT want this, you will need to inform the Editorial Office via email immediately. More information is available here: https://link.springer.com/partners/embo-press/editorial-policies#Peer%20review

EMBO Press Author Checklist

Corresponding Author Name: Lei Zhang, Yunyun Jin, Jian Wan
Journal Submitted to: EMBO Reports
Manuscript Number: EMBOR-2026-63711

USEFUL LINKS FOR COMPLETING THIS FORM
The EMBO Journal - Author Guidelines
EMBO Reports - Author Guidelines
Molecular Systems Biology - Author Guidelines
EMBO Molecular Medicine - Author Guidelines

Reporting Checklist for Life Science Articles (updated January
This checklist is adapted from Materials Design Analysis Reporting (MDAR) Checklist for Authors. MDAR establishes a minimum set of requirements in transparent reporting in the life sciences (see Statement of Task: 10.31222/osf.io/9sm4x). Please follow the journal's guidelines in preparing your
Please note that a copy of this checklist will be published alongside your article.

Abridged guidelines for figures
1. Data
The data shown in figures should satisfy the following conditions:
- the data were obtained and processed according to the field's best practice and are presented to reflect the results of the experiments in an accurate and unbiased manner.
- ideally, figure panels should include only measurements that are directly comparable to each other and obtained with the same assay.
- plots include clearly labeled error bars for independent experiments and sample sizes. Unless justified, error bars should not be shown for technical
- if n<5, the individual data points from each experiment should be plotted. Any statistical test employed should be justified.
- Source Data should be included to report the data underlying figures according to the guidelines set out in the authorship guidelines on Data

2. Captions
Each figure caption should contain the following information, for each panel where they are relevant:
- a specification of the experimental system investigated (eg cell line, species name).
- the assay(s) and method(s) used to carry out the reported observations and measurements.
- an explicit mention of the biological and chemical entity(ies) that are being measured.
- an explicit mention of the biological and chemical entity(ies) that are altered/varied/perturbed in a controlled manner.
- the exact sample size (n) for each experimental group/condition, given as a number, not a range;
- a description of the sample collection allowing the reader to understand whether the samples represent technical or biological replicates (including how many animals, litters, cultures, etc.).
- a statement of how many times the experiment shown was independently replicated in the laboratory.
- definitions of statistical methods and measures:
  - common tests, such as t-test (please specify whether paired vs. unpaired), simple χ2 tests, Wilcoxon and Mann-Whitney tests, can be unambiguously identified by name only, but more complex techniques should be described in the methods section;
  - are tests one-sided or two-sided?
  - are there adjustments for multiple comparisons?
  - exact statistical test results, e.g., P values = x but not P values < x;
  - definition of 'center values' as median or average;
  - definition of error bars as s.d. or s.e.m.

Please complete ALL of the questions below.
Select "Not Applicable" only when the requested information is not relevant for your study.

Materials

Newly Created Materials	Information included in the manuscript?	In which section is the information available? (Reagents and Tools Table, Materials and Methods, Figures, Data Availability Section)
New materials and reagents need to be available; do any restrictions apply?	Yes	Reagents and Tools Table, Materials and Methods

Antibodies	Information included in the manuscript?	In which section is the information available? (Reagents and Tools Table, Materials and Methods, Figures, Data Availability Section)
For **antibodies** provide the following information:<br>- Commercial antibodies: RRID (if possible) or supplier name, catalogue number and or/clone number<br>- Non-commercial: RRID or citation	Yes	Reagents and Tools Table, Appendix

DNA and RNA sequences	Information included in the manuscript?	In which section is the information available? (Reagents and Tools Table, Materials and Methods, Figures, Data Availability Section)
**Short novel DNA or RNA including primers, probes**: provide the sequences.	Yes	Materials and Methods, Appendix

Cell materials	Information included in the manuscript?	In which section is the information available? (Reagents and Tools Table, Materials and Methods, Figures, Data Availability Section)
**Cell lines:** Provide species information, strain. Provide accession number in repository **OR** supplier name, catalog number, clone number, and/**OR** RRID.	Yes	Reagents and Tools Table, Materials and Methods
**Primary cultures:** Provide species, strain, sex of origin, genetic modification status.	Yes	Materials and Methods
Report if the cell lines were recently **authenticated** (e.g., by STR profiling) and tested for mycoplasma contamination.	Yes	Materials and Methods

Experimental animals	Information included in the manuscript?	In which section is the information available? (Reagents and Tools Table, Materials and Methods, Figures, Data Availability Section)
**Laboratory animals or Model organisms:** Provide species, strain, sex, age, genetic modification status. Provide accession number in repository **OR** supplier name, catalog number, clone number, **OR** RRID.	Yes	Materials and Methods, Materials and Methods
**Animal observed in or captured from the field:** Provide species, sex, and age where possible.	Not Applicable	
Please detail **housing and husbandry conditions**.	Yes	Materials and Methods

Plants and microbes	Information included in the manuscript?	In which section is the information available? (Reagents and Tools Table, Materials and Methods, Figures, Data Availability Section)
**Plants:** provide species and strain, ecotype and cultivar where relevant, unique accession number if available, and source (including location for collected wild specimens).	Not Applicable	
**Microbes:** provide species and strain, unique accession number if available, and source.	Not Applicable	

Human research participants	Information included in the manuscript?	In which section is the information available? (Reagents and Tools Table, Materials and Methods, Figures, Data Availability Section)
If collected and within the bounds of privacy constraints report on age, sex and gender or ethnicity for all study participants.	Yes	Appendix, Materials and Methods

Core facilities	Information included in the manuscript?	In which section is the information available? (Reagents and Tools Table, Materials and Methods, Figures, Data Availability Section)
If your work benefited from core facilities, was their service mentioned in the acknowledgments section?	Yes	Materials and Methods

Design

Study protocol	Information included in the manuscript?	In which section is the information available? (Reagents and Tools Table, Materials and Methods, Figures, Data Availability Section)
If study protocol has been **pre-registered, provide DOI in the manuscript**. For clinical trials, provide the trial registration number **OR** cite DOI.	Not Applicable	
Report the **clinical trial registration number** (at ClinicalTrials.gov or equivalent), where applicable.	Not Applicable	

Laboratory protocol	Information included in the manuscript?	In which section is the information available? (Reagents and Tools Table, Materials and Methods, Figures, Data Availability Section)
Provide DOI OR other citation details if **external detailed step-by-step protocols** are available.	Yes	Materials and Methods

Experimental study design and statistics	Information included in the manuscript?	In which section is the information available? (Reagents and Tools Table, Materials and Methods, Figures, Data Availability Section)
Include a statement about **sample size** estimate even if no statistical methods were used.	Yes	Materials and Methods
Were any steps taken to minimize the effects of subjective bias when allocating animals/samples to treatment (e.g. **randomization procedure**)? If yes, have they been described?	Yes	Materials and Methods
Include a statement about **blinding** even if no blinding was done.	Not Applicable	
Describe **inclusion/exclusion criteria** if samples or animals were excluded from the analysis. Were the criteria pre-established?<br><br>If sample or data points were omitted from analysis, report if this was due to attrition or intentional exclusion and provide justification.	Not Applicable	
For every figure, are **statistical tests** justified as appropriate? Do the data meet the assumptions of the tests (e.g., normal distribution)? Describe any methods used to assess it. Is there an estimate of variation within each group of data? Is the variance similar between the groups that are being statistically compared?	Yes	Figures, Materials and Methods

Sample definition and in-laboratory replication	Information included in the manuscript?	In which section is the information available? (Reagents and Tools Table, Materials and Methods, Figures, Data Availability Section)
In the figure legends: state number of times the experiment was **replicated** in laboratory.	Yes	Figures
In the figure legends: define whether data describe **technical or biological replicates**.	Yes	Figures

Ethics

Ethics	Information included in the manuscript?	In which section is the information available? (Reagents and Tools Table, Materials and Methods, Figures, Data Availability Section)
Studies involving **human participants**: State details of **authority granting ethics approval** (IRB or equivalent committee(s), provide reference number for approval.	Yes	Materials and Methods
Studies involving **human participants**: Include a statement confirming that **informed consent** was obtained from all subjects and that the experiments conformed to the principles set out in the WMA Declaration of Helsinki and the Department of Health and Human Services Belmont Report.	Yes	Materials and Methods
Studies involving **human participants:** For publication of **patient photos**, include a statement confirming that consent to publish was obtained.	Not Applicable	
Studies involving experimental **animals**: State details of **authority granting ethics approval** (IRB or equivalent committee(s), provide reference number for approval. Include a statement of compliance with ethical regulations.	Yes	Materials and Methods
Studies involving **specimen and field samples:** State if relevant **permits** obtained, provide details of authority approving study; if none were required, explain why.	Not Applicable	

Dual Use Research of Concern (DURC)	Information included in the manuscript?	In which section is the information available? (Reagents and Tools Table, Materials and Methods, Figures, Data Availability Section)
Could your study fall under dual use research restrictions? Please check biosecurity documents and list of **select agents and toxins** (CDC): https://www.selectagents.gov/sat/list.htm	Not Applicable	
If you used a select agent, is the security level of the lab appropriate and reported in the manuscript?	Not Applicable	
If a study is subject to dual use research of concern regulations, is the name of the **authority granting approval and reference number** for the regulatory approval provided in the manuscript?	Not Applicable	

Reporting

The MDAR framework recommends adoption of discipline-specific guidelines, established and endorsed through community initiatives. Journals have their own policy about requiring specific guidelines and recommendations to complement MDAR.

Adherence to community standards	Information included in the manuscript?	In which section is the information available? (Reagents and Tools Table, Materials and Methods, Figures, Data Availability Section)
State if relevant guidelines or checklists (e.g., **ICMJE, MIBBI, ARRIVE, PRISMA**) have been followed or provided.	Yes	Materials and Methods
For **tumor marker prognostic studies,** we recommend that you follow the **REMARK** reporting guidelines (see link list at top right). See author guidelines, under 'Reporting Guidelines'. Please confirm you have followed these guidelines.	Not Applicable	
For **phase II and III randomized controlled trials**, please refer to the **CONSORT** flow diagram (see link list at top right) and submit the CONSORT checklist (see link list at top right) with your submission. See author guidelines, under 'Reporting Guidelines'. Please confirm you have submitted this list.	Not Applicable	

Data Availability

Data availability	Information included in the manuscript?	In which section is the information available? (Reagents and Tools Table, Materials and Methods, Figures, Data Availability Section)
Have **primary datasets** been deposited according to the journal's guidelines (see 'Data Deposition' section) and the respective accession numbers provided in the Data Availability Section?	Yes	Data Availability Section
Were **human clinical and genomic datasets** deposited in a public access-controlled repository in accordance to ethical obligations to the patients and to the applicable consent agreement?	Not Applicable	
Are **computational models** that are central and integral to a study available without restrictions in a machine-readable form? Were the relevant accession numbers or links provided?	Not Applicable	
If publicly available data were reused, provide the respective **data citations in the reference list**.	Yes	Reference
